# Decoupling of Neogene seawater lithium isotopes from uplift-driven weathering

**DOI:** 10.1038/s41467-026-71407-x

**Published:** 2026-05-15

**Authors:** Yibo Yang, Yudong Liu, Philip A. E. Pogge von Strandmann, Zhangdong Jin, Albert Galy, Chengcheng Ye, Mebrahtu F. Weldeghebriel, Zhongyi Yan, Jiajun He, Long-Fei Gou, Li Deng, Weilin Zhang, Andreas Koutsodendris, Jörg Pross, Xiaomin Fang

**Affiliations:** 1 State Key Laboratory of Tibetan Plateau Earth System, Environment and Resources (TPESER), Institute of Tibetan Plateau Research, Chinese Academy of Sciences, Beijing, China; 2 Mainz Isotope and Geochemistry Centre (MIGHTY), Institute of Geosciences, Johannes Gutenberg University, Mainz, Germany; 3 University of Chinese Academy of Sciences, Beijing, China; 4 State Key Laboratory of Loess Science, Institute of Earth Environment, Chinese Academy of Sciences, Xi’an, China; 5 Centre de Recherches Pétrographiques et Géochimiques, UMR7358, CNRS, Université de Lorraine, Nancy, France; 6 School of Geographical Sciences, East China Normal University, Shanghai, China; 7 Department of Geosciences, Princeton University, Princeton, NJ USA; 8 State Key Laboratory of Loess Science, Department of Geography, Chang’an University, Xi’an, China; 9 Institute of Earth Sciences and Cluster of Excellence GreenRobust, Heidelberg University, Heidelberg, Germany

**Keywords:** Palaeoclimate, Carbon cycle, Element cycles

## Abstract

The 9‰ increase in seawater lithium isotope (δ^7^Li) during the Cenozoic is widely regarded as evidence for uplift-driven enhancement of continental silicate weathering. Yet, the absence of long-term direct riverine δ^7^Li records has left this hypothesis largely untested. Given that the Tibetan Plateau contributes 16% of the modern global riverine Li flux, we present δ^7^Li records of paleowater spanning the past 15 million years covering the southern humid and northern arid Tibetan Plateau. Paleowater δ^7^Li reveals long-term decreases for the northern plateau, and low values for the southern plateau, reflecting a decrease in silicate weathering intensity in response to climatic cooling and high exhumations. Integrating our δ^7^Li record into a global Li cycle model, our study indicates that continental silicate weathering from tectonically active mountains is unlikely to have accounted for the observed rise in seawater δ^7^Li. These findings urge reconsideration of how tectonic uplift affects the chemistry of the ocean and carbon cycle.

## Abstract (web summary)

Neogene lithium isotope records from the Tibetan Plateau suggest that silicate weathering in mountain ranges may not persistently contribute sufficient lithium into the ocean to regulate seawater lithium isotope evolution

## Introduction

Chemical weathering of continental silicate rocks has played a critical role in maintaining Earth’s habitability via the regulation of atmospheric CO_2_ levels over geological timescales^1–3^. The lithium (Li) isotopes have become an effective tool for tracing and quantifying silicate weathering throughout Earth’s geological history owing to its insusceptibility to carbonate weathering and biological or redox-mediated fractionation^4–6^. The long-term increase in seawater δ^7^Li values over the Cenozoic is often considered as strong evidence for the uplift-weathering hypothesis^7^, which proposes that enhanced silicate weathering driven by the uplift of major mountain ranges (such as the Tibetan Plateau and the Andes) played a key role in atmospheric CO_2_ drawdown and global cooling during the Cenozoic^8,9^. This interpretation is largely based on modern observations identifying continental riverine input as the primary source of heavy δ^7^Li values in the ocean^4,7,10^. However, subsequent studies of the terrestrial Li cycle across both modern and orbital timescales suggest alternative mechanisms to explain the long-term increase in seawater δ^7^Li^6,11–17^. Concurrently, growing evidence from the marine Li cycle suggests that oceanic processes such as submarine hydrothermal input^18,19^, off-axis crustal alteration^20,21^, and reverse weathering^11,22^ have played a significant, and potentially dominant, role in driving the observed changes in seawater δ^7^Li during the Cenozoic.

**Fig. 1:**
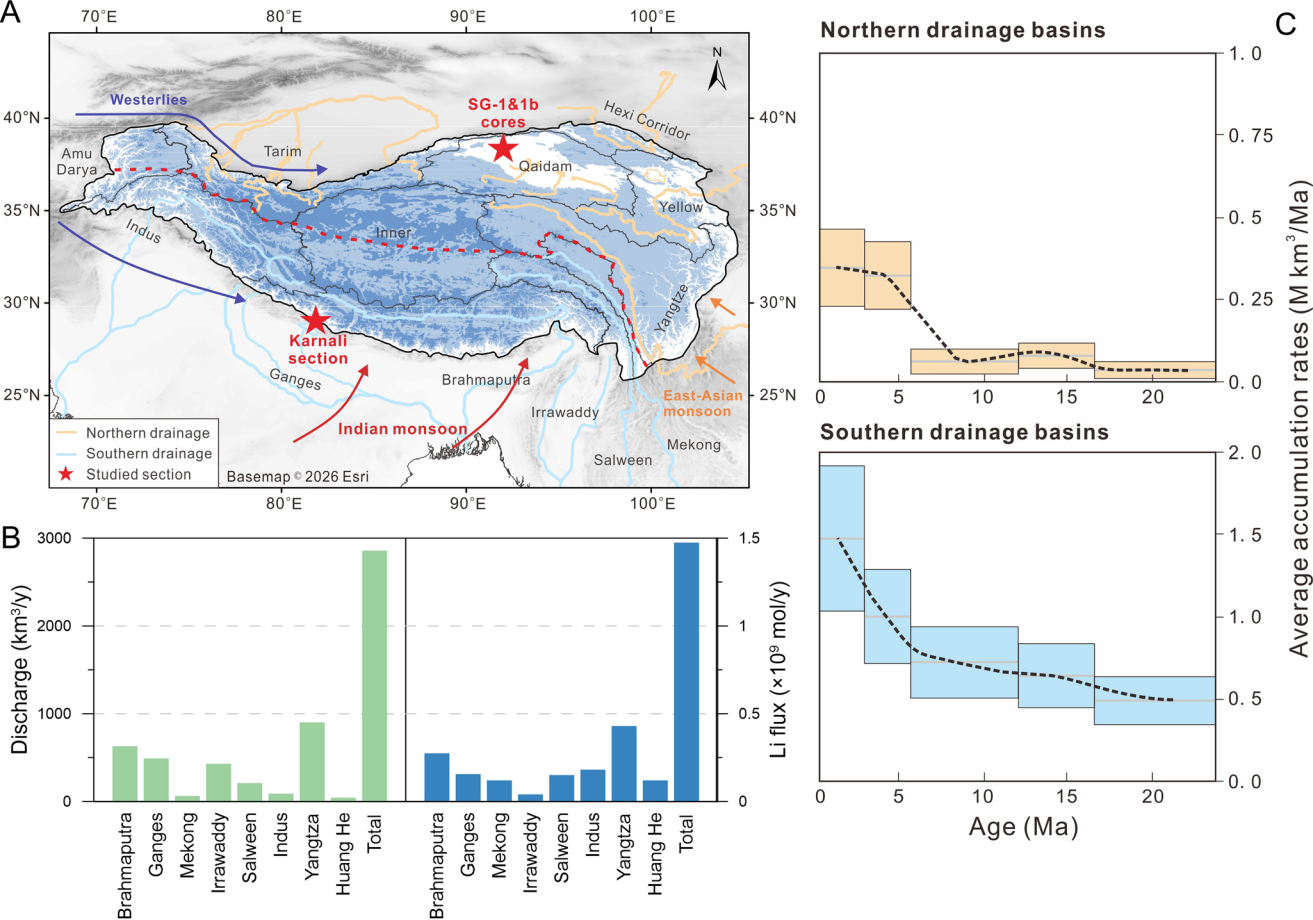
Riverine Li fluxes from the Tibetan Plateau. **A** Geographical map of the Tibetan Plateau with major rivers and the study sites (red stars). The red dashed line marked the boundary of the northern (rivers shown by yellow lines) and southern (rivers shown by light blue lines) drainage basins of the Tibetan Plateau. The base map is generated using ArcGIS and sourced from Esri (© 2026 Esri) with data from USGS and NOAA. **B** Discharge and dissolved Li flux of major rivers derived from the Tibetan Plateau (see Table 1 for details). **C** Sediment accumulation rates sourced from southern drainage basins (integrated from the submarine fans and marginal seas in south and SE Asia) and northern drainage basins (integrated from Central Asia and East China Sea Platform) (data from ref. ^64^).

Obtaining long-term Li isotope records directly from tectonically active regions, especially the Tibetan Plateau, is essential for understanding the influence of continental weathering on the oceanic Li cycle. While the Tibetan Plateau accounts for 1.5% of the global land area, it delivers ~10% of the global discharge and ~16% of the global river Li flux to the ocean (Fig. 1, Table 1). Although insights derived from modern and orbital-scale observations have increased our understanding of terrestrial Li cycling, many of the proposed mechanisms remain contradictory and fail to explain the contribution of riverine Li input over tectonic (million-year) timescales. To date, long-term riverine Li isotope records from orogenic regions are scarce due to several challenges. First, continuous sedimentary archives with reliable chronological constraints are rare in mountain ranges, largely because sedimentary environments are highly variable through time and space. Second, reconstructing the δ^7^Li evolution of ancient river water directly from terrestrial sediments is inherently difficult. Unlike marine deposits, continental sediments generally lack sufficient authigenic or biogenic carbonates, which are the best-understood archive for determining the Li isotopic composition of paleowaters. Third, pronounced contrasts in climate, lithology and erosion between the northern and southern Tibetan Plateau further complicate interpretations of reconstructed records, as records from any single locality may not capture the characteristics of the plateau as a whole. As a result, inferring the evolution of riverine δ^7^Li from the Tibetan Plateau over geologic timescales remains a significant challenge.

**Table 1:** Li and δ^7^Li fluxes from modern rivers draining the Tibetan Plateau

Rivers	Area^a^	Discharge^a^	Li concentration	Li flux	δ^7^Li	Location	Ref.
	10^6 ^km^2^	km^3^/yr	nM	10^9^ mol/yr	‰		
Chang Jiang (Yangtze)	1.80	900	478.0	0.430	21.3	Mouth	^86^
Brahmaputra	0.67	630	436.0	0.275	19.6	Mouth	^4^
Ganges	0.98	490	318.6	0.156	20.9	Mouth	^15^
Lancang (Mekong)	0.80	64	1880.0	0.120	13.6	Lower reaches	^87^
Huang He (Yellow)	0.75	43	2800.3	0.120	18.9	Middle reaches	^88^
Irrawaddy	0.43	430	94.1	0.040	–	Mouth	^89^
Salween	0.27	210	718.5	0.151	–	Mouth	^89^
Indus	0.98	90	2014.3	0.181	–	Upper reaches	^90,91^
Total^b^	6.68	2857		1.47	19.7		
Pearl	0.49	260	86.5	0.022	22.7	Mouth	^92^
Hong He (Red)	0.16	120	257.1	0.031	–	Lower reaches	^93^
Total^c^	7.33	3227		1.53	19.8		

^a^The area and discharge of rivers are sourced from a previous study^94^.

^b^The sum of data of modern rivers originating from the Tibetan Plateau (without Pearl River and Hong He).

^c^The sum of data of rivers originating from the Tibetan Plateau (with Pearl River and Hong He), taking into account that the formation and evolution of the Pearl River and Hong He are closely linked to the uplift of the Tibetan Plateau.

To constrain the contribution of riverine Li input from the Tibetan Plateau to the ocean over tectonic timescales, we established Neogene paleowater δ^7^Li records from both the southern and northern parts of the Tibetan Plateau, given the distinct climate and denudation regimes within the plateau. These records are based on a 3560-m-thick fluvial sequence (15–5 Ma) from the Siwalik foreland basin in the foothills of the Nepal Himalaya (Figure S1A) and a 1700-m-deep drill core (7.3–0.1 Ma) from the Qaidam Basin on the northern Tibetan Plateau (Figure S1B). The Siwalik Basin and Qaidam Basin lie within the southern Asian monsoon humid region and the mid-latitude westerlies arid region, respectively, and both are bordered by mountain ranges that experienced intensified tectonic uplift and climatic cooling during the Neogene^23–25^. Sediments in these basins are therefore ideal archives that typify change in silicate weathering associated with the Neogene uplift of the Tibetan Plateau. We provide the systematic reconstruction of Li isotopic variations across the northern and southern plateaus during the Neogene. It establishes a critical basis for evaluating how erosion and climate change jointly influence continental weathering and Li isotope behaviour, and provides key constraints on the contribution of continental weathering to the rise of seawater δ^7^Li.

## Results

**Fig. 2:**
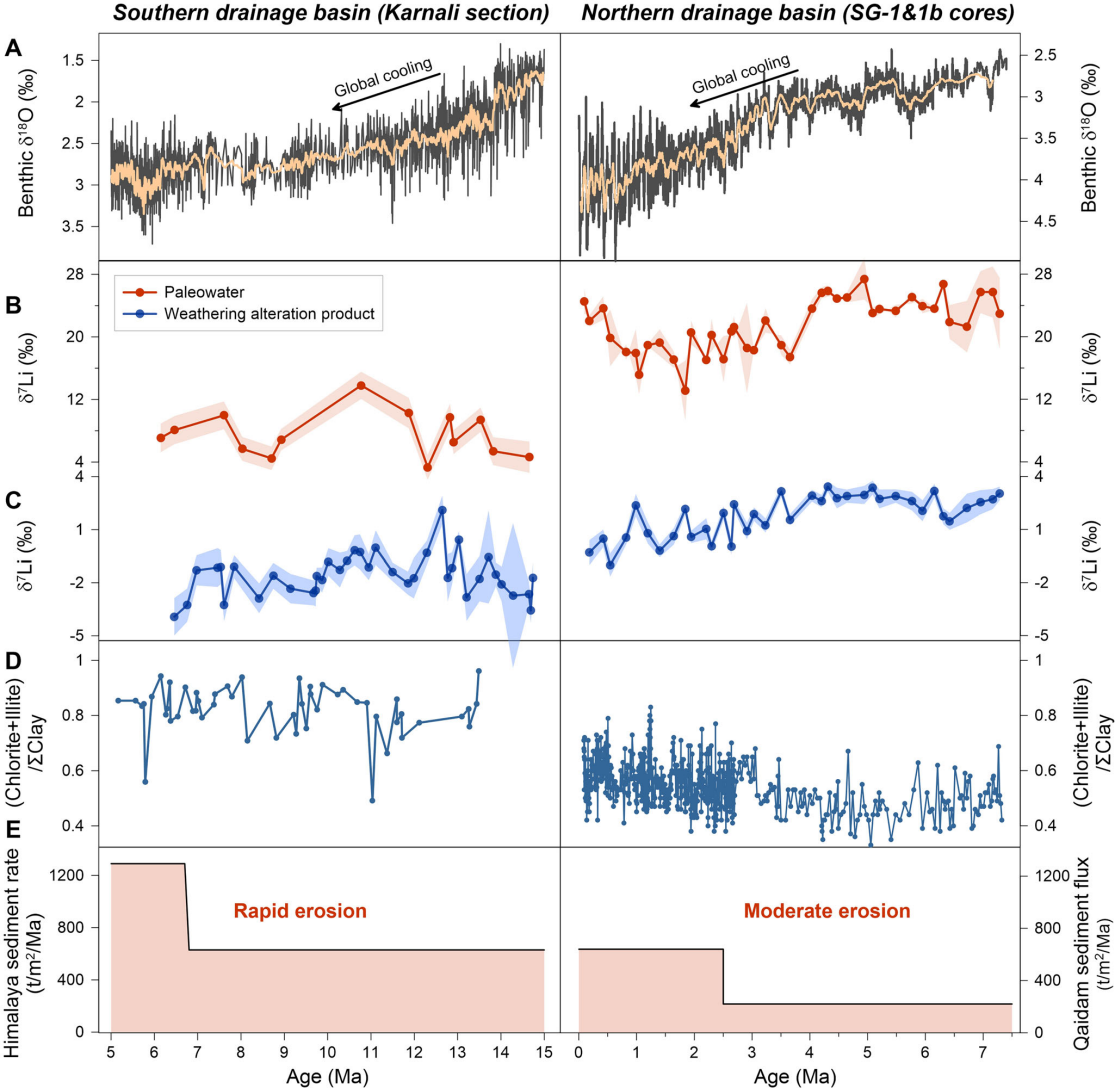
Multiphase Li isotope records of the southern and northern Tibetan Plateau compared with other climate, weathering and denudation records. **A** Global marine benthic foraminiferal δ^18^O record^52^, **B** paleowater δ^7^Li records based on carbonate and soluble salt fraction from the Nepal Himalaya and Qaidam Basin (pink shading represents the two standard errors (2SE) of the reconstructed values), **C** weathering alteration product δ^7^Li records corrected from clay fraction from the Nepal Himalaya and Qaidam Basin, **D** (chlorite+illite)/Σclay ratio of SG-1 and SG-1b cores as well as Bengal Fan sediment^95–97^, **E** sediment accumulation rates of Himalaya region and Qaidam Basin (data from refs. ^65,98^).

We analysed δ^7^Li values in carbonates, soluble salts, and the clay-size fraction in the sediments to reconstruct the Li isotopic compositions of both paleowater and their associated weathering alteration products (Fig. 2 and S2, Data S1 and S2). We first evaluate potential diagenetic effects and non-carbonate and detrital contamination during pretreatment. We then reconstruct the Li isotope composition of paleowater based on the measurements of carbonate fractions (which here refers to a mixture of calcite, aragonite and dolomite) and soluble salt fractions (here a mixture of halite and other chloride or sulfate salts) from the bulk sediments. Finally, we reconstruct δ^7^Li records of weathering alteration products based on clay-sized (<2 μm) fractions with the correction of unweathered rock fragments.

### Diagenetic effects, non-carbonate and detrital inputs

Diagenetic processes can modify the original Li isotopic signature of carbonate and clay minerals in sediments^26–28^. However, multiple lines of evidence suggest that diagenetic effects are minimal in this study (Figures S3–S4). There is no significant correlation between the carbonate δ^7^Li and Mn/Sr from both basins, suggesting weak meteoric diagenetic influence^29^ (Figure S3). The Li/(Ca+Mg) ratios of the carbonate fraction (Figure S3) are higher than those observed in meteorically altered low-Mg calcite^26^. The absence of correlation between Li/(Ca+Mg) and δ^7^Li further supports limited diagenetic influence. The higher Li/(Ca+Mg) ratios of the Qaidam Basin samples may result from evaporative concentration of salty lake water (the paleo-environment of the Qaidam Basin), a common feature in other lacustrine sediments^30^. Moreover, the water-soluble and carbonate phases show distinct δ^7^Li values but yield similar reconstructed evolution (Figure S2), implying that variations in mineralogy and porewater chemistry have not affected the Li isotope records in the Qaidam Basin. The aragonite-dominated carbonate mineralogy also indicates weak early diagenesis (Data S1). Regarding burial diagenesis, neither the δ^7^Li values of carbonate nor those of the <2 μm clay-sized fraction from the Qaidam Basin (SG‑1 and SG‑1b cores) or the Nepal Himalaya (Karnali section) show any decrease trend with depth (Figure S2). This indicates that diagenetic processes exerted only minor control over the Li isotopic composition. The clay minerals, which are exceptionally Li‑rich, did not experience significant compositional alteration due to burial diagenesis. Likewise, no notable increase in Li content or evidence of substantial modification was observed in the carbonate phases. Moreover, in the SG‑1 and SG‑1b cores, the proportion of smectite layers in illite‑smectite mixed layers (S%) increases with depth—a trend that contrasts with typical diagenetic progression patterns^31,32^ (Figure S4). Collectively, these observations provide compelling evidence that the analysed samples have retained their primary geochemical signatures with minimal diagenetic modification.

Paleowater δ^7^Li signals reconstructed from carbonates or soluble salt in fluvio-lacustrine environments are readily affected by non-carbonate inputs and detrital contamination during sample digestion^33,34^. To minimize this, first we used ultrapure water (18.2 MΩ*cm) to extract the water-soluble salts. Then, we used 1 M ammonium acetate (NH_4_Ac) to leach exchangeable lithium from clays and to dissolve remaining gypsum, if any, as a pre-cleaning step prior to carbonate digestion with 1 M acetic acid (HOAc)^34^. In all the 1 M acetic acid and water leachates of the bulk samples, the low Al/Ca, K/Ca, Mn/Ca, Al/(Cl+SO_4_), Rb/(Cl+SO_4_), and Si/(Cl+SO_4_) ratios and lack of correlation between δ^7^Li and those ratios suggest minimal influence from non-carbonate fractions (e.g., silicate fraction and Fe-Mn oxyhydroxides) in the leaching process (Figure S3). We minimized the detrital influence on δ^7^Li of soluble and carbonate fractions by selecting samples with high carbonate proportions ( > 3%, with an average of ~15%) through rigorous microscopic analysis of the samples. Meanwhile, carbonate δ^13^C and δ^18^O values of the Qaidam core sediments and bedrock samples from the surrounding source regions reveal little contamination from detrital carbonates (Figure S4). The δ^7^Li values of the soluble salt, carbonate, and exchangeable fractions of the sediments also show similar long-term trends in the Qaidam Basin (Figure S2), suggesting that the Li isotopic compositions of these phases likely recorded the same underlying processes. Microscopic observation revealed that in our measured samples with high carbonate content in the Nepal Himalaya sediments, authigenic carbonate was predominant (Figure S5), suggesting that the detrital influence is limited.

### Reconstructed δ^7^Li records of paleowater

To reconstruct the lithium isotopic composition of paleowater, we use fractionation-factor-corrected δ^7^Li values of carbonates and soluble salts, which are two main authigenic mineral phases preserved in our samples. The fractionation factors between carbonate or salt and water depend on the mineralogical composition^35–39^. The fractionation factors between various inorganic carbonate minerals and fluids are relatively well constrained ($-$10.7 ± 0.5‰ for aragonite, $-$8.5 ± 2‰ for inorganic calcite, and ~0 ± 1‰ for dolomite^26,36,39^). The stable Mg/Ca ratios of carbonate leachates for most samples suggest little variation in carbonate mineralogy throughout the sections (Figure S3). We therefore apply a weighted average of these fractionation factors, which is based on the relative proportions of the carbonate minerals, to reconstruct paleowater δ^7^Li values (Figure S6 and Data S4). Given that gypsum is relatively less soluble in water, the relatively constant and low Ca/Na and SO_4_/Cl ratios of water leachates indicate that most of the soluble salts are chloride salts (most likely halite). We compiled the available measurements from evaporation experiments, and we adopt an average fractionation factor between soluble salt and water, defined as ∆^7^Li_salt-water_ (δ^7^Li_salt_$-$δ^7^Li_water_), of ~0 ± 1‰^40–42^. This low fractionation factor is likely related to the majority of Li in halite being stored in non-fractionated fluid inclusions. We apply this fractionation factor to reconstruct paleowater δ^7^Li values from the soluble salts. The δ^7^Li values of paleowater reconstructed from both soluble salt and carbonate phases show similar trends and values, supporting the reliability and internal consistency of our approach (Figure S6). Intense rainfall in the Nepal Himalayas prevents the formation of evaporites in the sediments, so only carbonates serve for the reconstruction of the δ^7^Li values of paleowater in the Himalayan region. The paleowater δ^7^Li evolution curve is, therefore, based on the average of the two records (soluble salts and carbonates) for Qaidam Basin (i.e., northern Tibetan Plateau) and based on the carbonates only for Nepal Himalaya (i.e., southern Tibetan Plateau) (Figure S6). The paleowater δ^7^Li of the Nepal Himalaya shows low values (~10‰) with no temporal trend since ~15 Ma, while that of the Qaidam Basin shows a long-term decline from 24‰ ~7.3 Ma to 15‰ at 0.5 Ma, followed by a rapid increase from 0.5 Ma onwards (Fig. 2).

### Reconstructed δ^7^Li records of weathering alteration products

Weathering alteration products (i.e., clay minerals) provide an independent perspective on catchment weathering processes^28,30,34^. However, the Li isotopic compositions are susceptible to several confounding factors, including grain size, unweathered lithic fragments, recycled sedimentary rocks, and temperature-dependent fractionation^11,43,44^. We tried to minimize these potential biases. First, the influence of grain size is restricted by using the <2 μm fraction of sediments. Second, we use the relationship between the Weathering Index of Parker (WIP) and the Chemical Index of Alteration (CIA), which serves as a useful proxy for tracing shale recycling^45^, to evaluate the potential contribution of shale and the lithological characteristics of the source area. Shales represent lithified ancient weathering residues, which can lead to partial overlap in isotopic composition. The WIP-CIA relationship suggests that the impact of recycled sedimentary materials is minor (Figure S7). Focusing on the clay-sized fraction also maximises the enrichment in weathering products. Finally, we employ a correction method based on the mineralogical composition to account for the influence of unweathered debris^34^. X-Ray diffraction analysis of the <2 μm fraction of the core sediments reveals that unweathered minerals, primarily quartz, plagioclase, and K-feldspar, constitute a relatively stable and high proportion ( ~ 40%) of the samples (Figure S8). We use the known Li contents and δ^7^Li values of these unweathered minerals (ref. ^34^, Data S5) to reconstruct changes in the δ^7^Li values of weathering alteration products in the Nepal Himalaya and Qaidam Basin during the Neogene (Figure S6). After correcting for the influences of unweathered minerals, the corrected δ^7^Li values of weathering alteration products also exhibit no temporal trend for the Nepal Himalaya and a decreasing trend for the Qaidam Basin during the Neogene (Fig. 2). This trend aligns closely with the long-term changes in the paleowater δ^7^Li trend.

## Discussion

### Evaluating the impact of provenance change and crystallization fractionation

Although Li isotope fractionation is mainly linked to the formation of secondary minerals during weathering and is relatively insensitive to the composition of source rocks, the lithology of the source region can influence the types and abundances of clay minerals formed, thereby indirectly affecting Li isotope fractionation. To assess potential changes in sediment provenance, we utilized immobile element ratios such as Ti/Al, Th/Sc, and (La/Yb)_N_ as provenance indicators (Figure S9). These elements are largely unaffected by chemical weathering and thus preserve the geochemical signatures of the source rocks^46^. These provenance indicators in the <2 μm fraction of sediments show minimal temporal changes in the Qaidam Basin and Nepal Himalaya (Figure S9), thus indicating little change in provenance. In the Nepal Himalaya, we also compiled Nd isotopes, an effective tracer for distinguishing Himalayan geological units, to track the potential provenance change in the Siwalik sediments. The Nd isotope record indicates a shift in sediment provenance at ~8 Ma, potentially linked to the Lesser Himalayan exhumation^47–50^. Despite this change in source, the Li isotope compositions – recorded in both paleowater and weathering alteration product – remained constant. It appears that the Li isotope system is robust and largely unaffected by variations in provenance in this setting. Based on these observations, we conclude that the influence of sediment source on Li isotopic composition can be largely excluded both in the southern and northern Tibetan Plateau.

Fractionation associated with the precipitation of authigenic minerals has the potential to modify the isotopic evolution of Li in lake water, particularly under conditions of extensive mineral crystallization^51^. During mineral precipitation, light Li isotopes (^6^Li) preferentially partition into solid phases such as carbonates and evaporitic minerals, leading to an enrichment of ^7^Li in the residual water. This effect can be quantified using mineral–fluid fractionation factors (∆^7^Li_carb-fluid_ and ∆^7^Li_salt-fluid_). To assess the maximum possible influence of crystallization-driven fractionation on lake-water δ^7^Li, we applied a Rayleigh fractionation model to simulate δ^7^Li evolution under a range of fractionation factors. Model results indicate that if up to ~90% of dissolved Li is removed from the lake water and incorporated into authigenic minerals, representing the end-member scenario characteristic of a terminal, desiccating saline pan, the δ^7^Li of residual water would increase by 2.3‰ (*α* = 0.999), 6.9‰ (*α* = 0.997), 11.5‰ (*α* = 0.995), and 18.4‰ (*α* = 0.992, Figure S10A). Such extensive Li removal is unlikely during most stages of lake evolution because carbonates and evaporites are generally not Li-rich phases. However, in the final stage of paleolake desiccation, essentially all dissolved Li must ultimately be incorporated into newly precipitated minerals, making this scenario plausible for the late Quaternary (the last 0.5 Myr) evolution of the Qaidam Basin. The sedimentary facies of SG-1 and SG-1b cores support this interpretation. No substantial evaporite deposits are observed in either the Karnali section or the SG-1b core, suggesting that crystallization-driven Li isotope fractionation played a negligible role during earlier periods. In contrast, thick salt layers occur in the uppermost part of the SG-1 core (Figure S1), marking the onset of extensive evaporite formation. Correspondingly, Li concentrations in both water leachates and 1 M HOAc leachates increase sharply after ~0.5 Ma, indicating a substantial transfer of Li from lake water into newly precipitated authigenic minerals (Figure S10D). Most samples show no positive correlation between δ^7^Li and Li/Na (for soluble salts) or Li/Ca (for carbonates), except for the upper part of the SG-1 core younger than ~0.5 Ma, where such correlations emerge (Figure S10B, C). Although the Li isotope fractionation factor of halite is close to 0‰, gypsum and carbonate maybe still as the dominant driver of isotopic fractionation during this interval. Li concentrations in carbonates from the Qaidam record are an order of magnitude higher than those in water-soluble salts, supporting the interpretation that both are major hosts. Together, these lines of evidence suggest that crystallization-driven fractionation associated primarily with carbonate precipitation could have exerted a first-order control on paleowater δ^7^Li during the terminal stage of lake desiccation since ~0.5 Ma, while having little influence during earlier phases of lake evolution.

In addition, we evaluate the effect of temperature on Li isotope fractionation^11^. Based on the global and regional temperature drop ( ~ 6–10 °C) over the past 15 Myrs^52–54^, Li isotope fractionation during uptake by clays, which depends solely on temperature changes, would have been reduced by only ~1.5–1.9‰. Hence, the temperature effect is not sufficient to explain the changes in our records.

### Covariation of Li isotope records and silicate weathering

**Fig. 3:**
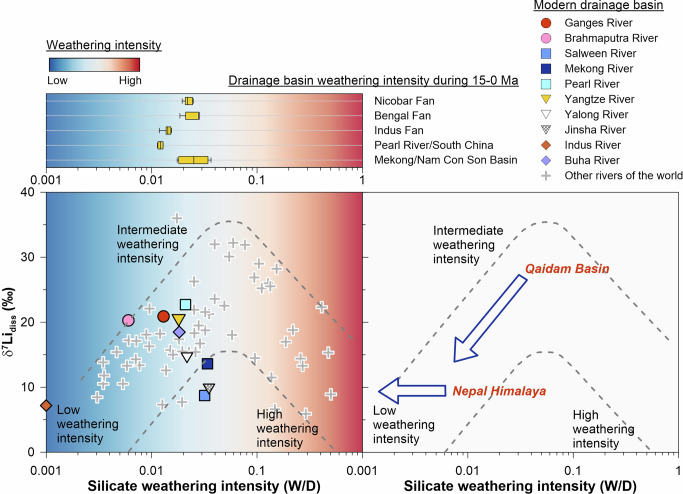
Weathering intensity of major basins surrounding the Tibetan Plateau, including modern and geological history (15–0 Ma). The silicate weathering intensity (W/D) is calculated as the silicate weathering rate/(chemical weathering rate + physical erosion rate). These findings indicate that the major drainage basins around the Tibetan Plateau are characterized by moderate to low weathering intensity from 15 Ma to the present. The box plots display the distribution of weathering intensity values, where the box spans the interquartile range (IQR, from 25th to 75th percentile), and the central line indicates the median. Data sources: Ganges River, Brahmaputra River, Salween River, Mekong River, Pearl River, and Yangtze River^6,80^; Yalong River and Jinsha River^62^; Indus River^90^; Buha River^17,99^; pattern of W/D with δ^7^Li and other rivers worldwide^6^; and estimated basin-scale weathering intensity in South and Southeast Asia from 15–0 Ma from ref. ^65^. The W/D ratios during 15–0 Ma are based on the silicate weathering flux (W) calculated from CO_2_ consumption flux and the reconstructed erosion flux (D).

The δ^7^Li of both paleowater and weathering alteration products is primarily controlled by the interplay between primary mineral dissolution and secondary mineral formation^6,55,56^. Given the established relationship between dissolved δ^7^Li and silicate weathering intensity (W/D) in modern rivers^6^, the observed trends in paleowater δ^7^Li values can be attributed to reduced weathering intensity under low to middle W/D ranges (Fig. 3; Figures S11 and S12). In the northern Tibetan Plateau, global cooling and regional aridification have led to medium-to-low silicate weathering intensity. This interpretation is supported by sediment geochemistry and clay mineral assemblages from the region (Figure S11 and S12). Regional weathering trends in the Qaidam Basin inferred from δ^7^Li records, together with elemental and mineralogical proxies, exhibit a consistent temporal pattern with global^52,53^ and regional^25^ climate indicators during 7.3–0.5 Ma (Figures S12 and S13). This synchronicity suggests that progressive regional aridification, ultimately driven by global cooling, was the dominant factor controlling Li isotope variations. The Qaidam Basin has always been outside the influence of the East Asian monsoon^25^, and global cooling, which reduced moisture transport by mid-latitude westerlies, likely suppressed the formation of secondary minerals such as clays and consequently diminished Li isotope fractionation. The decreasing isotopic offset between lake water and weathering products further reflects diminished fractionation under decreasing catchment weathering intensity (Figure S12).

In the Nepal Himalaya, although the regional climate is much humid under the influence of the South Asian monsoon, the Li isotope record shows little response to these climatic variations (Fig. 2). Instead, the persistently low and stable Li isotopic composition in both paleowater and secondary minerals indicates stable weathering conditions and low weathering intensity during the Neogene. This likely reflects the sustained high-erosion rates in the Himalayan region since the Neogene. Intense tectonic uplift combined with heavy monsoon rainfall has maintained rapid surface erosion, resulting in extremely short water–rock interaction times (Figure S14). This sustained rapid erosion regime during the Neogene is witnessed by exhumation records derived from ^10^Be, Sr-Nd isotope, and apatite fission-track lag-times during the Neogene^57–59^, as well as much clay mineralogy with a much higher content of illite and chlorite than those in the Qaidam Basin (Fig. 2). Such conditions promote primary mineral dissolution but limit the formation of secondary clays, thereby producing long-term low δ^7^Li values in river water. The reconstructed paleowater δ^7^Li values are broadly consistent with those of modern Himalayan rivers^15^, suggesting that this erosional regime has persisted for at least 15 million years. In addition, δ^7^Li of bulk sediments shows a stable and nearly ~0 value, which further confirms this^60^.

Although the Li isotope records from the southern and northern Tibetan Plateau show distinct long-term patterns, they can both be interpreted within the framework of silicate weathering intensity (W/D) and its associated processes of primary mineral dissolution and secondary mineral formation (Fig. 3). The apparent differences primarily reflect variations in regional erosion rates, which place their weathering conditions in different regimes. In the northern plateau, despite relatively active tectonics, the overall erosion flux remains moderate (Fig. 3). As a result, the effect of climatic cooling is amplified. In contrast, the Himalayas experience extremely high-erosion fluxes. When denudation (D) is extremely high, climate fluctuations become insufficient to drive detectable changes in the W/D ratio. The obtained records from the southern and northern Tibetan Plateau capture variations in paleowater δ^7^Li within the drainage areas of mountain ranges, reflecting the combined effect of Neogene tectonic uplift and global cooling.

### Global implications of riverine Li isotope changes from mountain ranges into the ocean

The reconstructed weathering Li isotope records across different exhumation and climate regimes of the Tibetan Plateau provide crucial evidence for assessing the relationship between plateau uplift-driven continental weathering and the seawater Li isotope evolution, both in terms of the Tibetan Plateau directly, but also as an analogue of uplift-driven weathering elsewhere. To examine the implications of our findings for seawater δ^7^Li changes, here we apply a dynamic model of the global Li cycle over the past 15 Myrs^61^. The model considers major Li sources, including continental rivers (*F*_riv_) and high-temperature hydrothermal fluids (*F*_MOR_), and major Li sinks (*F*_sed_), corresponding to alteration of the oceanic crust and uptake into marine sediments in addition to continental weathering. To assess the contribution of Tibetan Plateau rivers, the continental riverine flux was partitioned into two components: the riverine flux from the Tibetan Plateau (*F*_riv_TP_) and the riverine flux from other regions (*F*_riv_other_).

The model was constructed from a basic mass balance equation as follows:

1 $$\frac{{dN}}{{dt}}={F}_{{riv}{{\_}}{TP}}+{F}_{{riv}{{\_}}{ot}h{er}}+{F}_{{MOR}}-{F}_{{sed}}$$

The isotope balance equation is as follows:

2 $$N\frac{d{\delta {}^{7}{Li}}_{{sw}}}{{dt}}=	 {F}_{{riv}{{\_}}{TP}}\left({\delta {}^{7}{Li}}_{{riv}{{\_}}{TP}}-{\delta {}^{7}{Li}}_{{sw}}\right)+{F}_{{riv}{{\_}}{ot}h{er}}\left({\delta {}^{7}{Li}}_{{riv}{{\_}}{ot}h{er}}-{\delta {}^{7}{Li}}_{{sw}}\right) \\ 	+{F}_{{MOR}}\left({\delta {}^{7}{Li}}_{{MOR}}-{\delta {}^{7}{Li}}_{{sw}}\right)-{F}_{{sed}}\left({\delta {}^{7}{Li}}_{{sed}}-{\delta {}^{7}{Li}}_{{sw}}\right)$$

The calculation of the Li sink from seawater was based on the assumption that its partitioning into the sink occurred due to a constant partition coefficient k:

3 $${F}_{{sed}}=k\times N$$

where N is the seawater Li reservoir, F_x_ represents the input and output fluxes, and δ^7^Li_x_ is the isotope ratio of the various fluxes. The dynamic model of Li was modelled in 10,000-year time steps. The model parameters are described in Table 2 and the Methods section.

**Table 2:** Description of the parameters in the dynamic model of the Li cycle

Symbol	Description	Value	References and notes
F_riv_	Continental river Li flux	9.36 × 10^9^ mol/yr	^83^
δ^7^Li_riv_	Continental river δ^7^Li	18.8‰	^83–85^
F_riv_TP_	River Li flux of the Tibetan Plateau	1.47 × 10^9^ mol/yr	Table 1
δ^7^Li_riv_TP_	River δ^7^Li of the Tibetan Plateau	19.7‰	Table 1
F_riv_other_	River Li flux of the other regions of the world	7.89 × 10^9^ mol/yr	Calculated based on F_riv_ and F_riv_TP_
δ^7^Li_riv_other_	River δ^7^Li of the other regions of the world	18.6‰	Calculated based on F_riv_, δ^7^Li_riv_, F_riv_TP_, and δ^7^Li_riv_TP_
F_MOR_	High-temperature MOR hydrothermal Li flux	5.18 × 10^9^ mol/yr	^7,81–83^
δ^7^Li_MOR_	High-temperature MOR hydrothermal δ^7^Li	7.1‰	^61,83^
F_sed_	Li flux related to low-temperature altered oceanic crust and uptake onto marine sediments	~14.5 × 10^9^ mol/yr	^61^
Δ_sink_	Isotopic fractionation related to low-temperature altered oceanic crust and uptake onto marine sediments	~15‰	^7,83^
N	Seawater Li reservoir	3.65 × 10^16^ mol	^7^

**Fig. 4:**
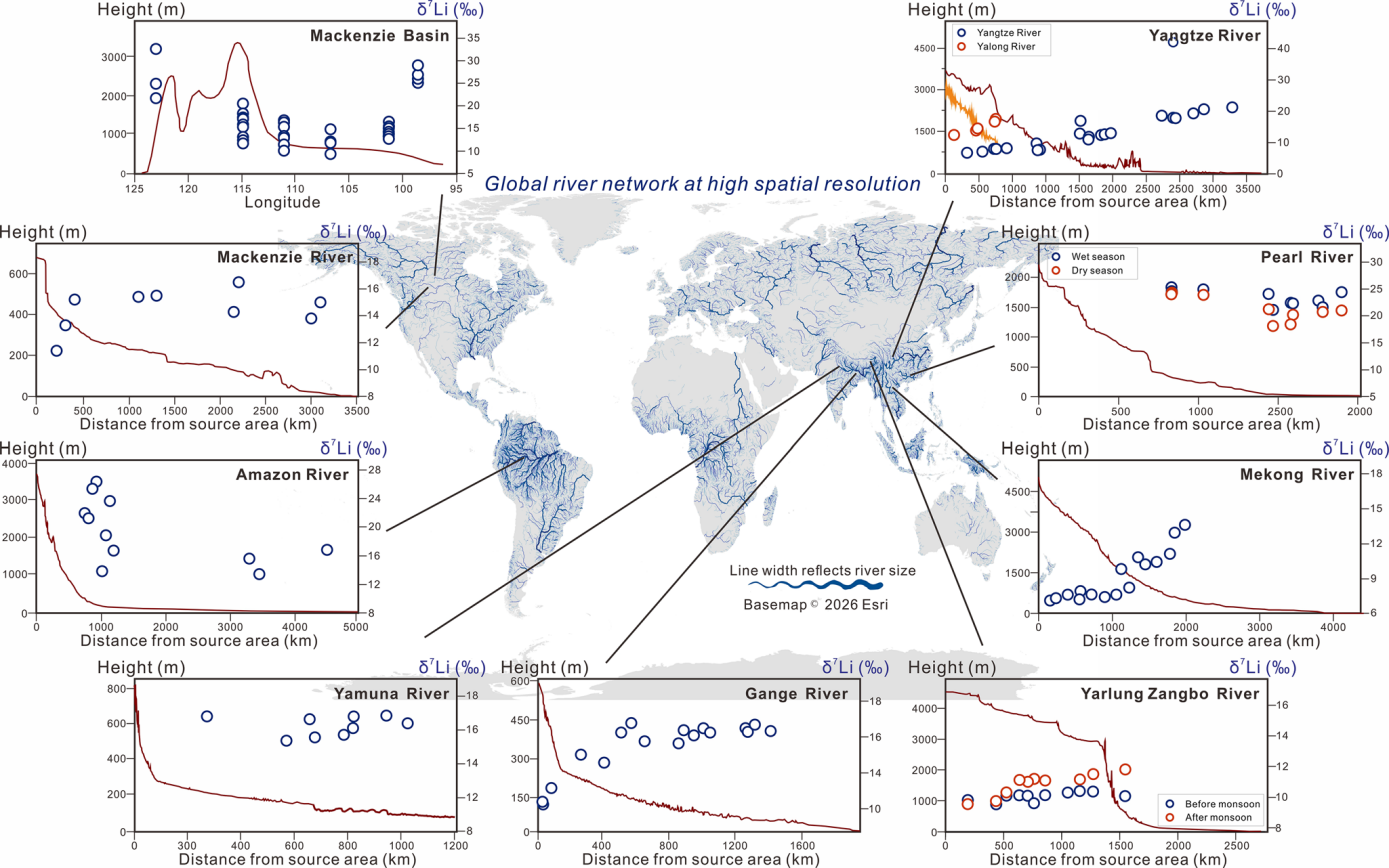
Assessment of the effects of weathering in floodplains on riverine Li isotopes. Globally, the dissolved δ^7^Li in rivers increases by approximately 6–10‰ from mountainous areas to floodplains, but soon stabilizes and ceases to rise further with increasing distance across the floodplain. The global riverine δ^7^Li values are from previous studies^6,15,62,70,86,87,92,100^. The central basemap is generated using ArcGIS and sourced from Esri (© 2026 Esri). River data are from the HydroRivers dataset (HydroSHEDS).

Before evaluating the impact of the riverine Li flux from the Tibetan Plateau on seawater, the potential influences of floodplain areas on weathering and Li isotopes must be considered^14,15,62^. A synthesis of global riverine δ^7^Li studies has revealed that riverine δ^7^Li values increase from the mountains to floodplains by approximately 6-10‰, as shown by the Ganges, Yangtze, Yarlung Zangbo, Mekong, and Pearl Rivers (Fig. 4). However, this increase soon reaches an equilibrium state and does not continue with increasing distance from the floodplain (Fig. 4). Although δ^7^Li tendency in floodplains can be complex (e.g., plateauing or even decreasing downstream in some modern cases^63^ or probably varying influences of floodplains in the past periods), our compilation, based on data from major world rivers, provides a representative overview of the general trends. These results suggest that paleowater δ^7^Li records from mountain ranges (e.g., the Tibetan Plateau) can still capture the stable or decreasing trends of riverine δ^7^Li exported from tectonically active areas to the oceans.

**Fig. 5:**
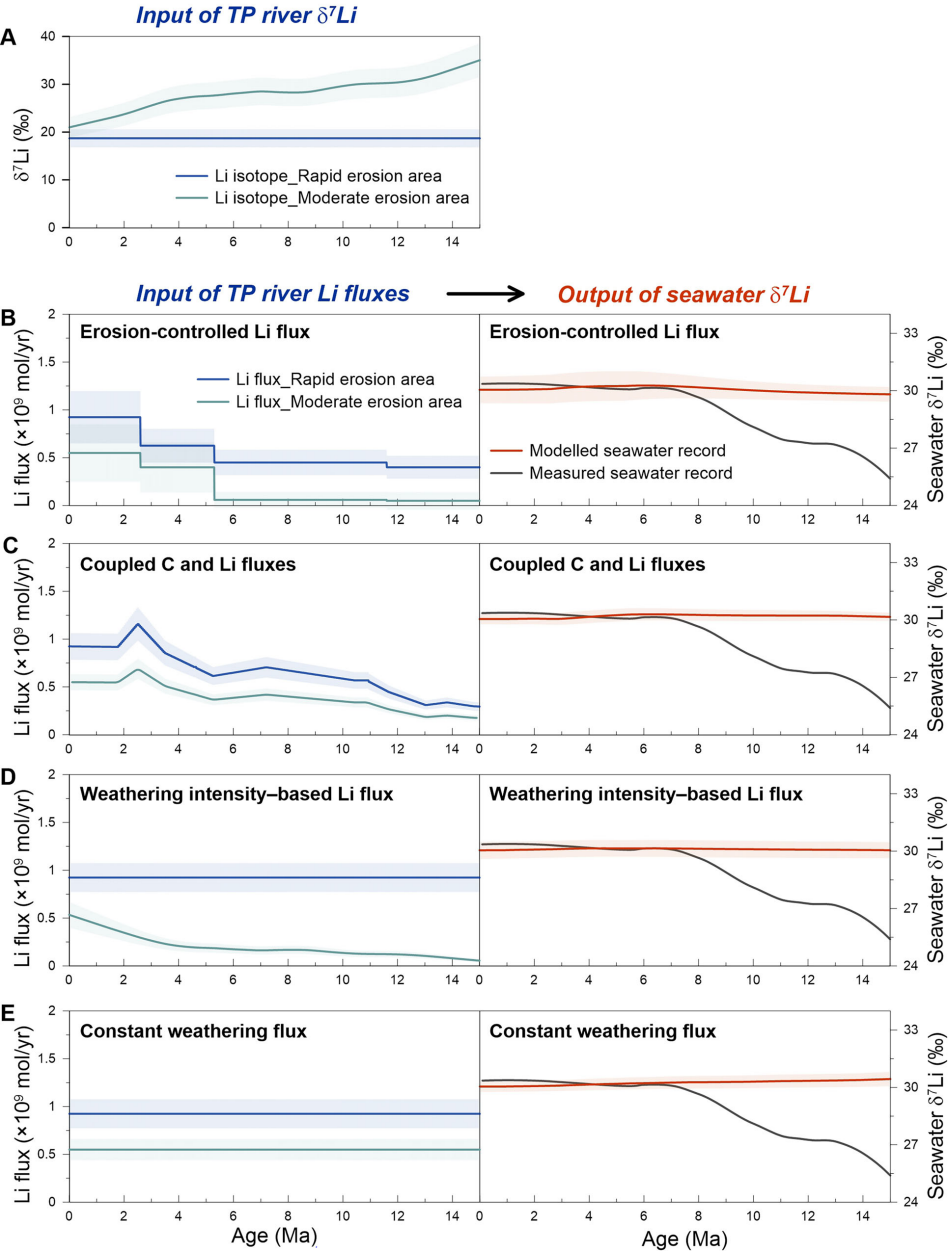
Dynamic simulation of seawater Li isotope changes under reconstructed Li fluxes and δ^7^Li values from the Tibetan Plateau (TP). **A** The evolution of δ^7^Li_riv_ of the rapid erosion area (blue line) and moderate erosion area (green line) of the Tibetan Plateau. **B**–**E** The evolution of input river Li fluxes and model result of seawater δ^7^Li, **B** based on sediment accumulation rates of surrounding basins^64^ (Scenario 1), **C** coupled with C and Li fluxes^13,65^ (Scenario 2), **D** based on correlation between Li yield, δ^7^Li, and W/D^66^ (Scenario 3), **E** based on constant weathering flux (based on Be isotope records)^67^ (Scenario 4). The measured seawater δ^7^Li curve is from ref. ^7^. See methods for details of the four scenarios. None of the four scenarios can reproduce the rise in seawater δ^7^Li since 15 Ma. Shading areas represent calculation uncertainty (2SE).

It is essential to constrain both the Li flux and isotopic composition input from the Tibetan Plateau. Reconstructing past variations in riverine Li flux from the Tibetan Plateau remains highly challenging due to the absence of direct reconstructions of ancient river discharge and Li concentrations. We first quantified the modern riverine Li fluxes across the plateau through statistical analyses (Table 1). The results indicate that major rivers originating from the Tibetan Plateau collectively export a Li flux of approximately ~1.5×10^9 ^mol/yr to the ocean (accounting for ~16% of global river input) (Table 1). We then employed four scenarios to constrain the Li flux evolution of the Tibetan Plateau over the past 15 Myrs (see Methods). First, the past Li flux varied proportionally with the Neogene erosion flux of the northern and southern plateau (Fig. 1B)^64^. Second, the atmospheric CO_2_ consumption flux by silicate weathering of the Tibetan Plateau^65^ constrain the dissolved Li flux based on the C-Li coupling assumption that Li is congruently released, similar to the way base cations (Na, K, Ca and Mg) are depleted during silicate weathering^13^ (Fig. 5C). Third, the Li flux through time is constrained by the empirically derived relationship with δ^7^Li, the weathering-to-denudation (W/D) ratio from modern rivers^66^, and our measured δ^7^Li signatures (Fig. 5D). Fourth, temporal changes in Li flux is linked to the constant exhumation and erosion rates of the Tibetan Plateau^47,58,67^ (Fig. 5E). Subsequently, we tested the effect of these four different scenarios separately in our Li dynamic model and these four scenarios essentially encompass the current understanding of the weathering-induced lithium flux on the Tibetan Plateau during the Neogene period.

Using our reconstructed record, we can further constrain the variations in overall lithium isotope composition related to plateau weathering. To differentiate between regional weathering patterns, we used the sediment flux from Central Asia and the East China Sea platform to estimate the dissolved Li export flux in moderate erosion regions, and used sediment flux from the sub-marine fans and marginal seas in South and Southeast Asia to estimate that in high-erosion regions (Fig. 1). Based on our δ^7^Li records, we assumed that the Li isotope composition in high-erosion regions (similar to the Nepal Himalaya) remained constant since 15 Ma, while in moderate erosion regions (similar to the Qaidam Basin), it gradually decreased with global climate (here, we set δ^7^Li to correlate with benthic δ^18^O). Although there might be cases of river basin reorganization during this period, the large-scale drainage reorganization that have been reported, such as the Yellow River and Irrawaddy River^68,69^, are completely occurred within the high and moderate erosion regions delineated in our study. These drainage reorganizations would not alter the total sediment and Li fluxes of the high and moderate erosion regions. Therefore, we can derive a comprehensive Li isotope composition for the entire plateau output by weighting the regional isotope values according to their respective Li flux contributions. Our model results suggest that, regardless of increasing or constant riverine Li flux from the Tibetan Plateau over the past 15 Myrs, the estimated Li weathering flux from the Tibetan Plateau can only cause the tendency of seawater δ^7^Li to be relatively flat (Fig. 5). These calculations demonstrate that riverine input from the Tibetan Plateau, driven by rapid erosion and global cooling, is unlikely to account for the observed rise in seawater δ^7^Li at least over the past 15 Myrs.

Collectively, the results of the modelling that takes into account our records suggest that Neogene seawater δ^7^Li records are unlikely to reflect enhanced silicate weathering driven by mountain uplift, due to the mixing of other source and sink fluxes. Our results do not imply that weathering of the Tibetan Plateau is incapable of influencing Earth’s carbon cycle. Rather, they indicate that the Li flux supplied by silicate weathering of tectonically active regions alone may be insufficient to measurably alter the marine Li cycle. This implies that changes in the alteration of the oceanic crust, uptake of Li into marine sediments, and continental weathering outside active mountain belts have to be taken into account to explain the δ^7^Li seawater record in the last 15 Ma. Indeed, the oceanic Li budget is strongly modulated by seafloor hydrothermal inputs and submarine reverse weathering^11,18,19^, which may outweigh the continental contribution. In addition, Li export from continents can be substantially influenced by non-weathering processes, including hydrological variability and aridity^16,17^, as well as inputs from geothermal waters^70^. By examining the Cenozoic uplift of the Tibetan Plateau as a natural experiment, our study demonstrates the limitations of using marine Li isotopes alone to infer long-term weathering-driven CO_2_ consumption. We therefore emphasize the importance of reconstructing silicate weathering histories in major orogenic regions, such as the Tibetan Plateau and the Andes, which provide the most direct geological archives of tectonically driven carbon cycling. Our Li isotope records and modelled results underscore that the Li cycle associated with hydrothermal systems and submarine reverse weathering is needed to explain the observed Neogene seawater δ^7^Li records, and that it is important to better constrain these complex marine source-sink processes.

## Methods

### Studied sections and chronology

The Karnali section is situated in the western Nepal Himalaya region on the southern Tibetan Plateau and contains about 3560-m-thick fluvial sequence from the Lower to the Upper Siwalik^71^. The sequence is characterized by a typical fluvial coarsening upward succession, from fine-grained meandering river sandstones alternating with mudstones, siltstones and oxidised calcareous paleosols in the Lower Siwalik to the thick channel sandstones and drab-coloured histosols in the Middle Siwalik^71^. The age framework of the 3560-m-thick sediments in the Karnali section has been well constrained by magnetostratigraphy results with an age range between 15.8 Ma and 5.2 Ma^71^.

The SG-1 and SG-1b cores, situated in the western Qaidam Basin on the northeastern Tibetan Plateau, were obtained as part of the Sino-German International Cooperation Program for the Tibetan Plateau (TiP-TORP). The SG-1 borehole extends to a depth of 938.5 m, with an average recovery rate of approximately 95%, while the SG-1b borehole reaches a depth of 723 m, with an average recovery rate of approximately 93%. The recovery rates for salt layers (predominantly at the upper SG-1 core) exceed 90%, and for clastic layers, they generally exceed 98%, ensuring nearly continuous stratigraphic records throughout the cores^72,73^. The core samples predominantly comprise blocky mudstones interbedded with siltstones or sandstones, which are rich in carbonates. The upper sections of the SG-1 core are particularly rich in evaporite minerals (e.g., halite and gypsum) (Figure S1). High-resolution magnetostratigraphic analyses of both cores, along with optically stimulated luminescence dating of the uppermost strata, have established the ages of the SG-1 core to be from 2.7 to 0.1 Ma and those of the SG-1b core to be approximately 7.3–1.6 Ma (Fig. 1)^72,73^. The combination of these two cores provides a continuous record of fine-grained lacustrine sedimentation spanning the last 7.3 Myrs.

### Samples and sequential leaching

The sediments from the Karnali section and SG-1 and SG-1b cores were used for multiphase extraction and elemental, mineralogical, and isotopic analyses. The samples were dried in an oven at 40 °C and ground into a fine powder (below 200 mesh size). Approximately 0.5 g of each sample was weighed and transferred into a 50-mL centrifuge tube. Sequential leaching was performed using ultrapure water (18.2 MΩ*cm) to extract soluble salt fractions, 1 mol/L ammonium acetate (pH~7) to remove exchangeable Li fractions from clay, and 1 mol/L acetic acid to digest the carbonate fractions, respectively, based on the sequential leaching method^34^. After centrifugation, the supernatant was filtered through a 0.22-μm filter membrane for subsequent analysis. The residual material was further treated with 10% acetic acid to ensure complete removal of carbonates, leaving an acid-insoluble residue^34^. The <2 μm fraction of samples was isolated via gravitational sedimentation based on Stokes’ law following the removal of organic matter and carbonate under the addition of 25% H_2_O_2_ and 10% acetic acid^74^. Both the acid-insoluble residues and the extracted <2 μm fractions were heated at 600 °C in a muffle furnace for 10 hours to remove organic matter. Then, digestion was conducted using nitric acid (HNO_3_) and hydrofluoric acid (HF), following the treatment^34,75^. The separation of the <2 μm fraction was performed at the Micro Structure Analytical Laboratory, Peking University, Beijing. The sequential leaching and digestion procedures of samples from the Karnali section and SG-1 and SG-1b cores were carried out in the laboratory of the Institute of Tibetan Plateau Research, Chinese Academy of Sciences, Beijing and the Institute of Earth Environment, Chinese Academy of Sciences, Xi’an, separately.

### Elemental, mineral and C-O isotopes analyses

The major and trace element concentrations in all leachates were analysed via inductively coupled plasma-optical emission spectrometer (ICP-OES) and inductively coupled plasma-mass spectrometer (ICP-MS) at the Institute of Tibetan Plateau Research, Chinese Academy of Sciences, Beijing. Replicate analyses revealed a relative standard deviation of <5%, ensuring the precision and reliability of the measurements.

The mineral compositions of both the whole rock and the <2 μm fraction of sediment were determined using a Bruker D8+ diffractometer at the Institute of Tibetan Plateau, Chinese Academy of Sciences, Beijing. The instrument was operated under the following conditions: Cu Ka, 1.5406 Å, 40 kV, 100 mA, 3–30°, 0.02° step increment, and 10°/min.

The carbon and oxygen isotope values of the samples were measured using a GasBench II gas preparation device online with a Delta V Plus isotope ratio mass spectrometer (Thermo Finnigan) at the Institute of Geology and Geophysics, Chinese Academy of Sciences, Beijing. The precision of the carbon and oxygen isotope measurements exceeded 0.06‰ and 0.1‰, respectively. All the carbon and oxygen isotope values were reported as per mil (‰) relative to Vienna Pee Dee Belemnite (VPDB).

### Li isotope analysis

A total of 179 leachates derived from three phases (water-leachate, 1 mol/L acetic acid-leachate, and <2 μm fraction) were measured for Li isotopes. The SG-1 and SG-1b cores samples were purified for Li using cation exchange resin (Bio-Rad®AG50W X-12) with 0.5 mol/L HNO_3_ as the eluent following the methods of ref. ^76^ at the State Key Laboratory of Loess and Quaternary Geology, Institute of Earth Environment, Chinese Academy of Sciences. The Karnali section samples were purified for Li using cation exchange resin (Bio-Rad®AG50W X-12) with 0.2 mol/L HCl as the eluent following the methods of ref. ^65^ at the Laboratory of the Centre for Research and Education on Biological Evolution and Environment, Nanjing University. To ensure analytical accuracy, the water leachates (i.e., soluble salt fraction) underwent column separation twice to achieve a Na/Li ratio lower than 1 ppm/ppm. The complete recovery of Li was assessed by analyzing solution splits collected before and after bracketing for the lithium content. The Li recovery rates of the samples all exceeded 99.9%.

Li isotopes of SG-1 and SG-1b core samples were measured by a multicollector inductively coupled plasma mass spectrometer (MC-ICP-MS, Neptune plus) at the Institute of Earth Environment, Chinese Academy of Sciences. Each sample was analyzed in triplicate to obtain average values and standard deviations (s.d.). The Li isotopes of Karnali section samples were measured by a multicollector inductively coupled plasma mass spectrometer (MC-ICP-MS) at the Lab of Centre for Research and Education on Biological Evolution and Environment, Nanjing University. The sample-standard-bracketing (SSB) method was used to correct instrument mass bias. δ^7^Li was reported using the traditional

delta notation:

4 $${{{\rm{\delta }}}}{}^{7}{Li}\left({\%_{0}}\right)=\left(\frac{{\left(\,7{Li}/\,6{Li}\right)}_{{sample}}}{{\left(\,7{Li}/\,6{Li}\right)}_{L-{SVEC}}}-1\right)\times 1000$$

To validate the accuracy of the measurements, two rock reference materials (AGV-2 and BHVO-2) and a seawater reference material (NASS-6) were processed using the same purification procedure and analyzed repeatedly, yielding δ^7^Li values of 6.3 ± 0.1‰ (*n* = 4), 3.6 ± 0.1‰ (*n* = 3), and 30.7 ± 0.4‰ (*n* = 10), respectively. These findings were all consistent with published data^66,77–79^.

### Correction of the Li isotopes of the clay fraction (<2 μm)

A correction method based on mineral compositions^34^ was applied to correct for nonclay minerals (unweathered debris) in the sediments, which can be expressed as follows:

5 $$\delta {}^{7}{Li}_{{clay}}=\frac{\left(\delta {}^{7}{Li}_{ < 2\mu m}\times {C}_{ < 2\mu m}^{{Li}}-\delta {{}^{7}{Li}}_{{Qtz}}{\times C}_{{Qtz}}^{{Li}}\times {f}_{{Qtz}}-\delta {{}^{7}{Li}}_{{Kfs}}\times {C}_{{Kfs}}^{{Li}}\times -\\ \delta {{}^{7}{Li}}_{{Pl}}\times {C}_{{Pl}}^{{Li}}\times {f}_{{Pl}}\right)}{{C}_{{clay}}^{{Li}}}$$

where ${{{{\rm{C}}}}}_{{{{\rm{x}}}}}^{{{{\rm{Li}}}}}$ refers to the Li content of a certain component or mineral end-member x; ${{{{\rm{f}}}}}_{{{{\rm{x}}}}}$ represents the content proportion of a certain mineral x in the <2 μm fraction; and Qtz, Kfs, Pl, and clay are quartz, K-feldspar, plagioclase, and total clay minerals, respectively. The contents of unweathered minerals of the <2 μm fraction of sediments, primarily quartz, plagioclase, and K-feldspar, were obtained by X-ray diffraction analysis (Figure S8 and Data S1-S2). The Li content and isotopic composition of these unweathered minerals were summarized in Data S5.

### The determination of parameters in the dynamic model

To quantify both the Li flux and the Li isotopic composition (δ^7^Li) of riverine outputs from the Tibetan Plateau, we partitioned the plateau into two endmember weathering regimes—high-erosion areas and moderate-erosion areas—based on our study and sediment flux patterns. The total riverine Li export was thus calculated as the sum of contributions from these two regions.

Firstly, we determine the evolution of δ^7^Li in high- and moderate-erosion regions. For high-erosion regions (e.g., Himalaya), we assume that δ^7^Li_riv_highE_ has remained constant over the past 15 Myrs. This value is constrained using modern river measurements and reflects persistent high weathering congruency through time. For moderate-erosion regions, the temporal evolution of δ^7^Li_riv_moderateE_ cannot be treated as constant. Instead, we infer the variations by interpolating δ^7^Li values from the empirical positive relationship between benthic δ^18^O and paleowater δ^7^Li (Figure S13). This approach links global cooling and weakened weathering intensity to long-term changes in dissolved Li isotopic signatures. The δ^7^Li trajectory for this region therefore gradually decreases toward the present, following the climatic trend recorded by the benthic δ^18^O stack. After establishing δ^7^Li curves for both regions (Fig. 5A), we calculate the plateau-wide isotopic composition of riverine Li input by flux-weighting the two δ^7^Li values according to their respective Li flux contributions. This yields the final time-dependent δ^7^Li curve used as the riverine boundary condition in the global Li cycle model.

Secondly, we determine the evolution of Li flux from the Tibetan Plateau. The total Li flux exported by rivers from the plateau through time was evaluated under  four independent scenarios. Scenario 1: Erosion-controlled Li flux reconstruction. Based on the global correlation between erosion rate and silicate weathering rate^80^, we use the reconstructed Neogene erosion flux history of the Tibetan Plateau^64^ to derive its corresponding Li flux evolution. This scenario assumes that erosion is the primary driver of long-term Li delivery. Scenario 2: Coupling carbon and lithium fluxes. During the silicate weathering process, the leaching of base cations is the primary mechanism responsible for the weathering-derived carbon sink. As base cations are leached, Li is simultaneously released; thus, the Tibetan Plateau weathering-derived carbon sink varies directly as changes in the dissolved Li flux from silicate weathering^13^. Recent findings by Clift et al. ^65^ provide key constraints on the evolution of silicate weathering-induced carbon consumption fluxes in the southern Tibetan Plateau. Building on the reconstruction^65^, we assume that Li fluxes scale proportionally with CO_2_ consumption fluxes by silicate weathering, thereby enabling reconstruction of a Li flux record intrinsically linked to silicate weathering. Nevertheless, quantitative records of such fluxes across other regions of the plateau remain sparse, which results in a possible bias in our calculation. This scenario allows Li flux to respond to the carbon cycle. Scenario 3: Weathering intensity-based Li flux estimation. We apply modern empirically derived relationships among Li flux, δ^7^Li, and the silicate weathering intensity (W/D)^66^. Under this framework, variations in δ^7^Li directly reflect shifts in weathering congruency, which in turn can be translated into time-varying Li fluxes. This scenario allows Li flux to be inferred solely from isotope systematics. Scenario 4: Constant weathering flux constrained by global marine and Himalayan ^10^Be records^58,67^. Under this assumption, the Li flux from the Tibetan Plateau remains constant through time, which is according with the stable erosion flux during the past 6 Myrs^58^ and steady state of erosion regimes during the past 13 Myrs^57,59^, in the Himalaya. Each of these three Li-flux histories is tested independently in the global Li cycle model to examine its influence on seawater δ^7^Li evolution.

To better distinguish spatial patterns of continental weathering, sediment flux data from adjacent basins (^64^, Fig. 1) are used to assess whether their export signals represent northern or southern plateau sources. Basins draining toward Central Asia and the East China Sea platform are categorized as moderate-erosion regions, whereas those draining toward the Indian subcontinent and Indochina correspond to high-erosion regions. Modern Li flux endmembers are assigned accordingly: moderate-erosion regions take the Yellow and Yangtze Rivers as analogues, whereas high-erosion regions use the Ganges, Brahmaputra, Indus, Mekong, Salween, and Irrawaddy Rivers. Combining the region-specific δ^7^Li histories and the three alternative Li-flux scenarios, we obtain a time-resolved, flux-weighted δ^7^Li signal representing the total Li exported from the plateau.

The ${{{{\rm{F}}}}}_{{{{\rm{MOR}}}}}$ was set to 5.18 × 10^9 ^mol/yr with ${{{{\rm{\delta }}}}{}^{7}{{{\rm{Li}}}}}_{{{{\rm{MOR}}}}}$ = 7.1‰^61,81–83^. ${{{{\rm{F}}}}}_{{{{\rm{riv}}}}\_{{{\rm{rivTP}}}}}$ was calculated to be 1.47 × 10^9 ^mol/yr, with an initial ${{{{\rm{\delta }}}}^{7}{{{\rm{Li}}}}}_{{{{\rm{riv}}}}\_{{{\rm{rivTP}}}}}$ value of 19.7‰, based on river discharge, Li concentrations, and Li isotopic compositions of modern rivers (Table 1). Incorporating recent findings from global studies on major rivers like the Amazon and the Yangtze, the estimated average Li isotopic compositions of continental rivers have been updated^83–85^. Here, we adopt the average value of these revised isotopic values with 18.8‰ as the continental river endmember. Given the updated continental riverine Li flux and δ^7^Li of 9.36 × 10^9 ^mol/yr with δ^7^Li = 18.8‰, ${{{{\rm{F}}}}}_{{{{\rm{riv}}}}\_{{{\rm{other}}}}}$ could be calculated to be 7.89 × 10^9 ^mol/yr, with a ${{{{\rm{\delta }}}}^{7}{{{\rm{Li}}}}}_{{{{\rm{riv}}}}\_{{{\rm{other}}}}}$ of 18.6‰. ${{{{\rm{\delta }}}}{}^{7}{{{\rm{Li}}}}}_{{{{\rm{sink}}}}}$ was given by ${\triangle }_{{{{\rm{sink}}}}}={{{{\rm{\delta }}}}{}^{7}{{{\rm{Li}}}}}_{{{{\rm{sw}}}}}-{{{{\rm{\delta }}}}{}^{7}{{{\rm{Li}}}}}_{{{{\rm{sink}}}}}$ (i.e., isotopic fractionation during reverse weathering), where ${\triangle }_{{{{\rm{sink}}}}}$ = 15‰^7,83^. During the model runs, *F*_riv_other_, δ^7^Li_riv_other_, and δ^7^Li_MOR_ were considered as long-term invariants.

## Supplementary information

Supplementary Information

Description of Additional Supplementary Files

Dateset S1-S5

Transparent Peer Review file

## Data Availability

All data are available in the Supplementary Materials and Supplementary Data S1 to S5.
