# Supplementary material for: Decoupling of Neogene seawater lithium isotopes from uplift-driven weathering
Source: Nat Commun. 2026 May 15;17:6524. doi: 10.1038/s41467-026-71407-x (PMC13376809; doi:10.1038/s41467-026-71407-x)
Supplement: Supplementary file 1 — Supplementary Information [file 41467_2026_71407_MOESM1_ESM.pdf]

Supplementary Materials for

Decoupling of seawater lithium isotopes from uplift-driven weathering

Yibo Yang†*, Yudong Liu†*, Philip A.E. Pogge von Strandmann, Zhangdong Jin*, Albert Galy, Chengcheng Ye, Mebrahtu F. Weldeghebriel, Zhongyi Yan, Jiajun He, Long-Fei Gou, Li Deng, Weilin Zhang, Andreas Koutsodendris, Jörg Pross, Xiaomin Fang

†These authors contribute equally
*Corresponding author. Email: yangyibo@itpcas.ac.cn, liuyudong@itpcas.ac.cn, and zhdjin@ieecas.cn

This PDF file includes:

Figs. S1 to S14

Other Supplementary Material for this manuscript includes the following:

Data S1 to S5

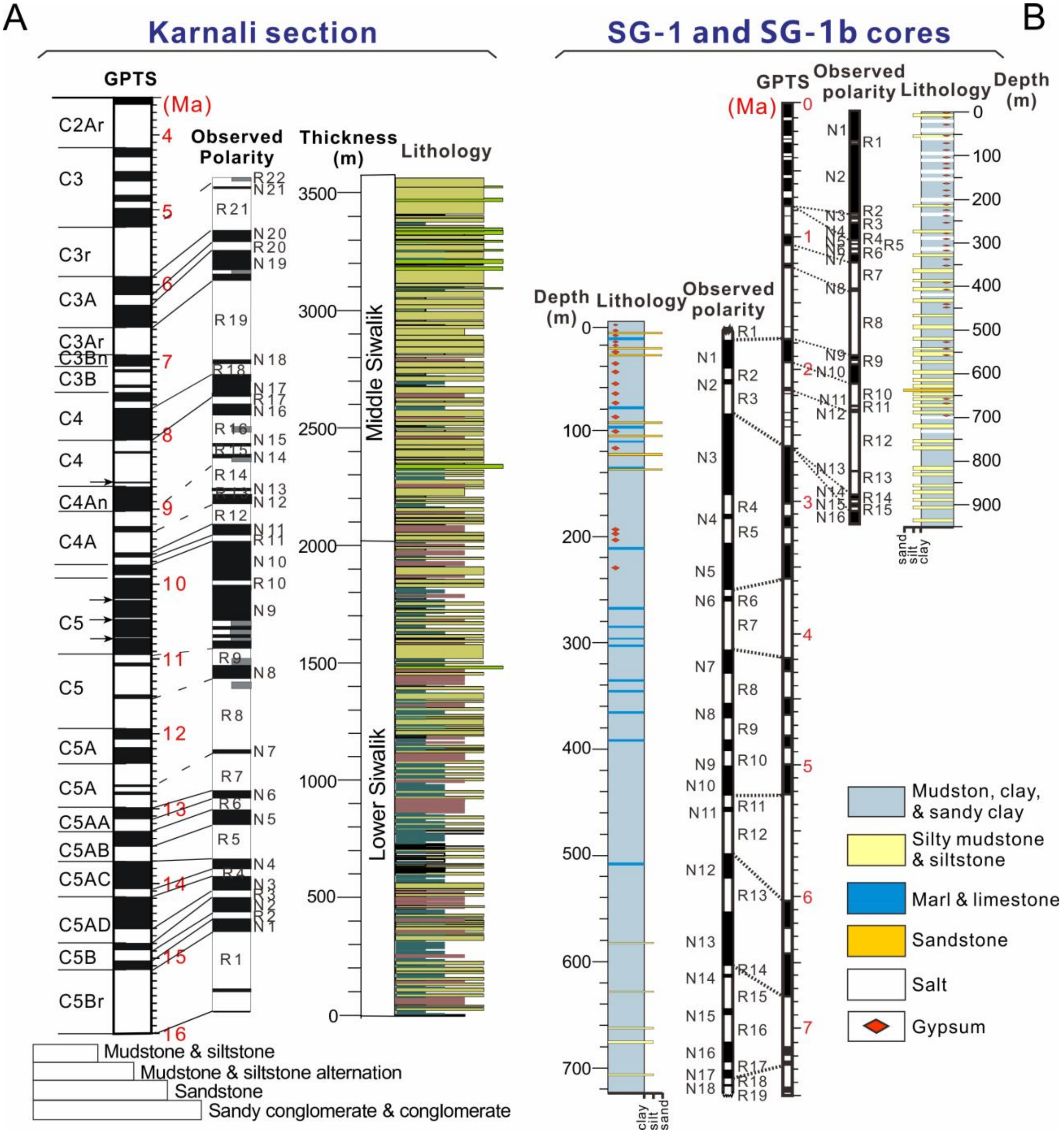

Fig. S1.

The lithologies and magnetostratigraphy of the Karnali section (**A**) and the SG-1 and SG-1b cores (**B**) (modified after refs. *71-73*).

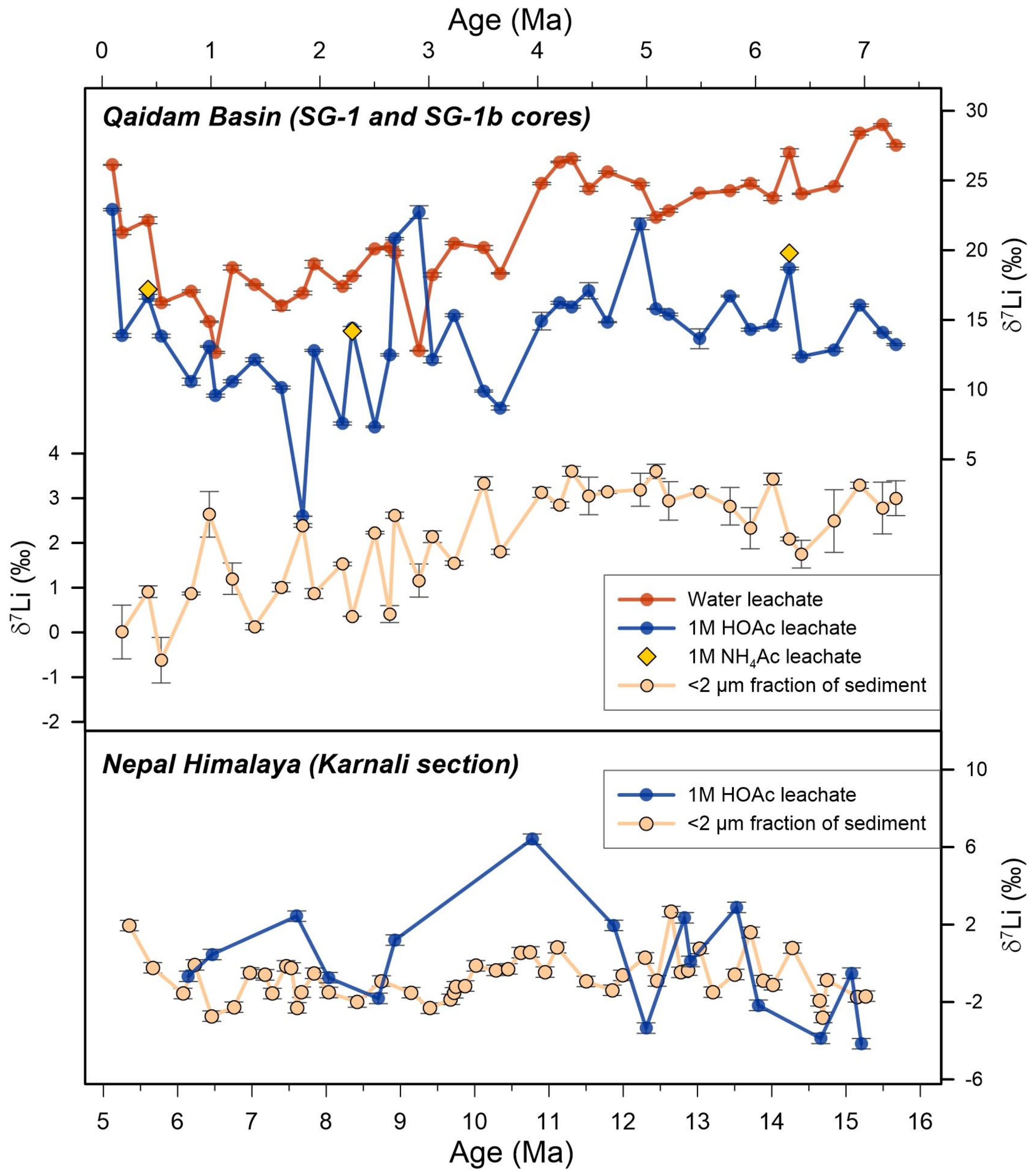

Fig. S2.

Multi-phase Li isotope records of the Nepal Himalaya and Qaidam Basin, including water leachate, 1M acetic acid (HOAc) leachate, and <2 μm fraction of sediment. The data of 1M ammonium acetate (NH_4Ac) leachate are from ref. (*34*). Error bars represent analytical error (2SE).

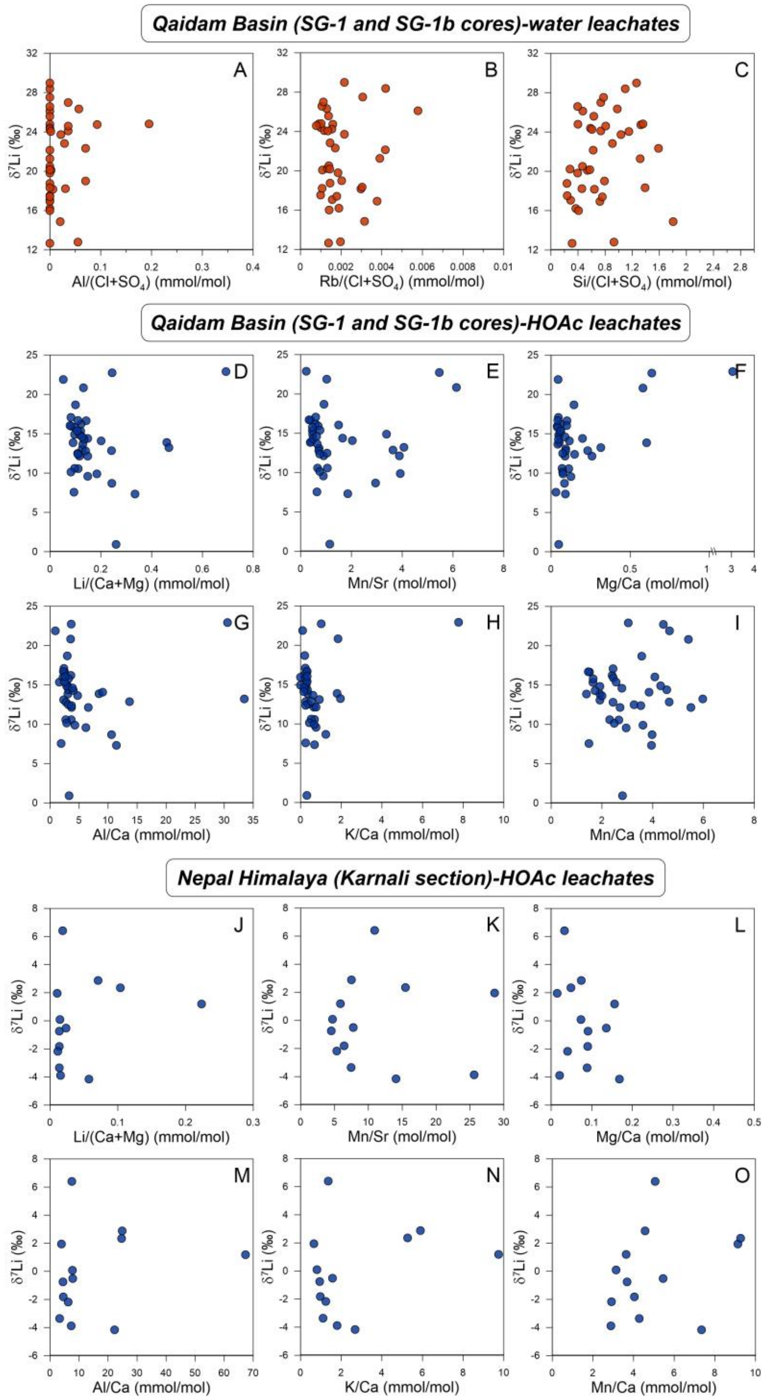

Fig. S3.

Correlation of δ^7Li and elemental ratios in water leachates (soluble salt fractions) and 1M HOAc leachates (carbonate fractions) of sediments. (**A-C**) Relationships between δ^7Li and Al/(Cl+SO_4), Rb/(Cl+SO_4), and Si/(Cl+SO_4) in water leachates from the SG-1 and SG-1b cores, (**D-I**) relationships between δ^7Li and Li/(Ca+Mg), Mn/Sr, Mg/Ca, Al/Ca, K/Ca, and Mn/Ca in HOAc leachates from the SG-1 and SG-1b cores, (**J–O**) relationships between δ^7Li and Li/(Ca+Mg), Mn/Sr, Mg/Ca, Al/Ca, K/Ca, and Mn/Ca in HOAc leachates from the Karnali section.

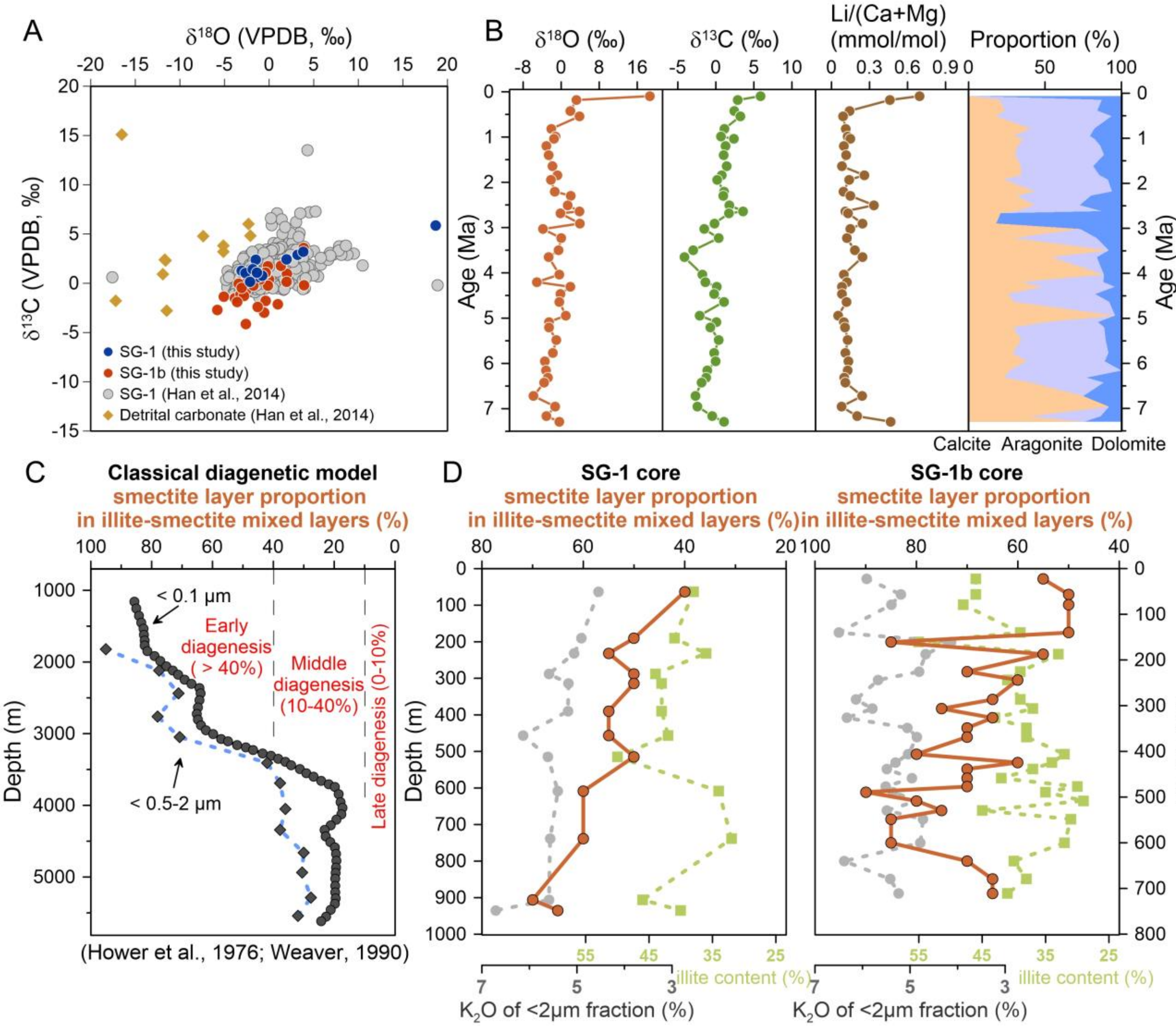

Fig. S4.

Evaluation of diagenetic effects and detrital inputs of the SG-1 and SG-1b cores. **(A)** Correlation between $\delta^{13}C$ and $\delta^{18}O$ of sediment carbonate from the SG-1 and SG-1b cores and detrital carbonate from the surrounding area, **(B)** variations of $\delta^{13}C$, $\delta^{18}O$, Li/(Ca+Mg), and carbonate minerals contents, **(C)** classical diagenetic model, **(D)** diagenetic indicators records of the SG-1 and SG-1b cores.

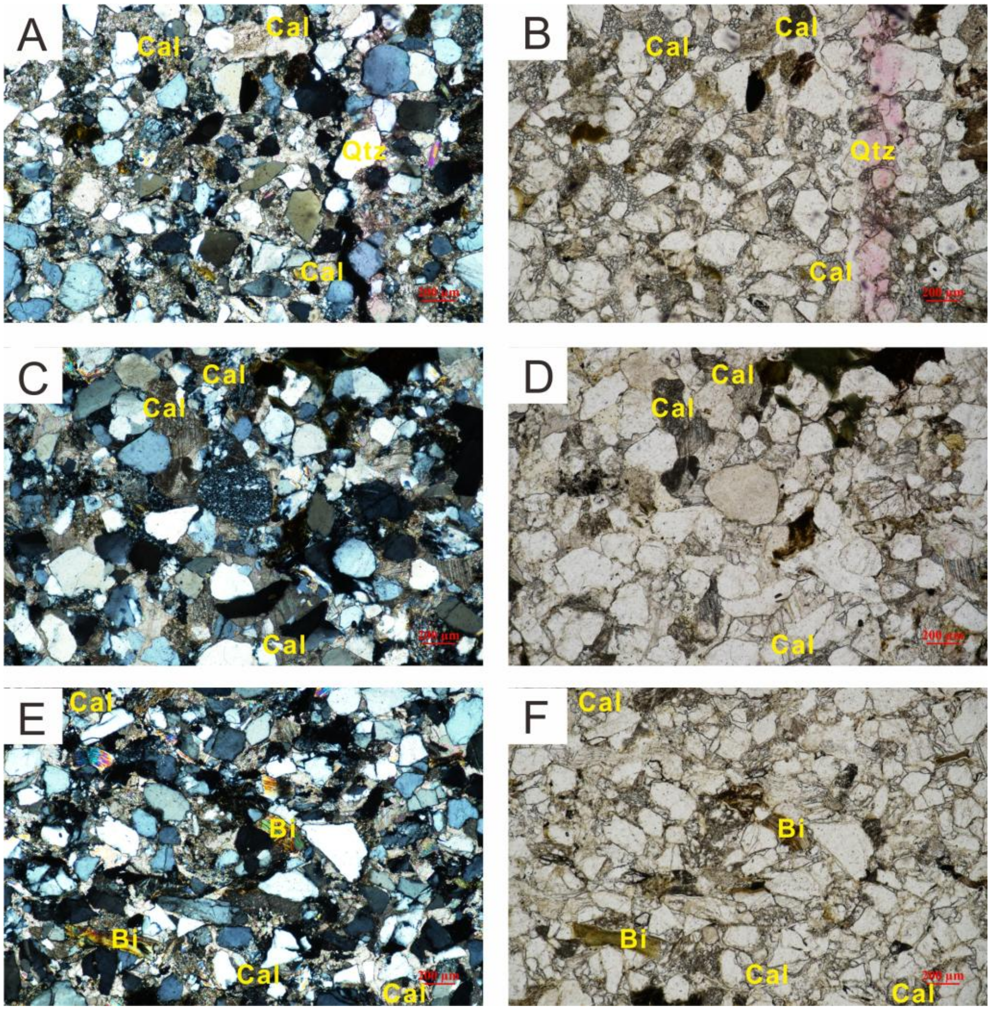

Fig. S5.

Photomicrographs of representative samples of the Karnali section. The texture and mineral assemblage of samples in crosspolarized light (XPL) **(A, C, E)** and plane-polarized light (PPL) **(B, D, F)** showing predominantly authigenic carbonate cements. Cal: carbonate, Qtz: quartz, Bi: biotite, Lv: lithic volcanic.

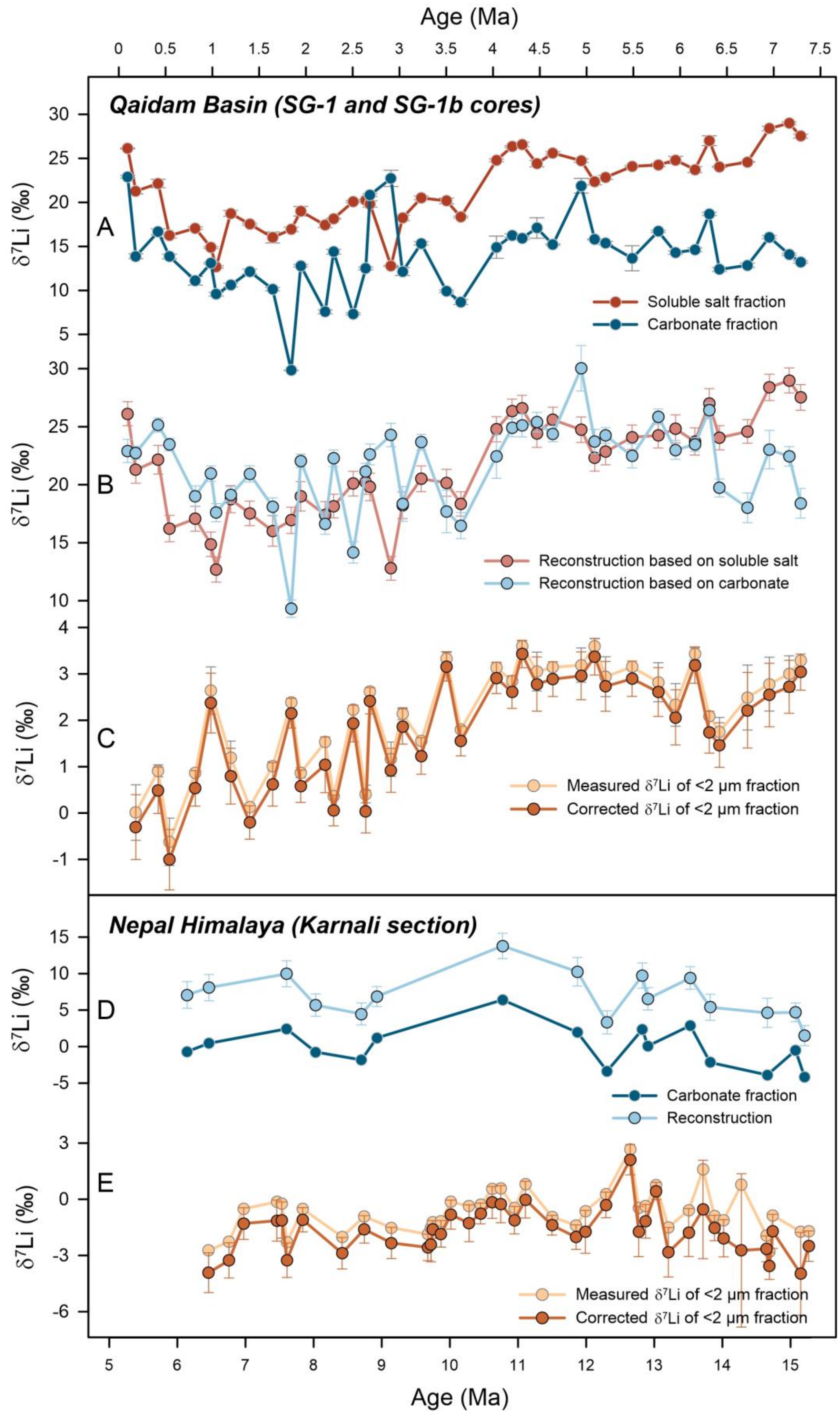
Age (Ma)
Qaidam Basin (SG-1 and SG-1b cores)
δ7Li (‰)
A
B
C
D
E
Soluble salt fraction
Carbonate fraction
Reconstruction based on soluble salt
Reconstruction based on carbonate
Measured δ7Li of <2 μm fraction
Corrected δ7Li of <2 μm fraction
Nepal Himalaya (Karnali section)
Carbonate fraction
Reconstruction
Measured δ7Li of <2 μm fraction
Corrected δ7Li of <2 μm fraction
Age (Ma)

Fig. S6.

Reconstructed δ^7Li records of paleowater and weathering alteration product from the Tibetan Plateau. **(A)** Measured δ^7Li records of soluble salt and carbonate of sediments from the Qaidam Basin (error bars represent analytical error, 2SE), **(B)** reconstructed δ^7Li records of paleowater in the Qaidam Basin based on soluble salt and carbonate phase (error bars represent the uncertainty in the fractionation factor, 2SE), **(C)** the measured and corrected δ^7Li records of clay fraction of sediments from the Qaidam Basin (error bars represent analytical error and error generated by corrected calculation, respectively, 2SE), **(D)** the measured and reconstructed δ^7Li records of paleowater based on carbonate fraction of sediments from the Nepal Himalaya (error bars represent analytical error, 2SE), **(E)** the measured and corrected δ^7Li records of clay fraction of sediments from the Nepal Himalaya (error bars represent analytical error and error generated by corrected calculation, respectively, 2SE).

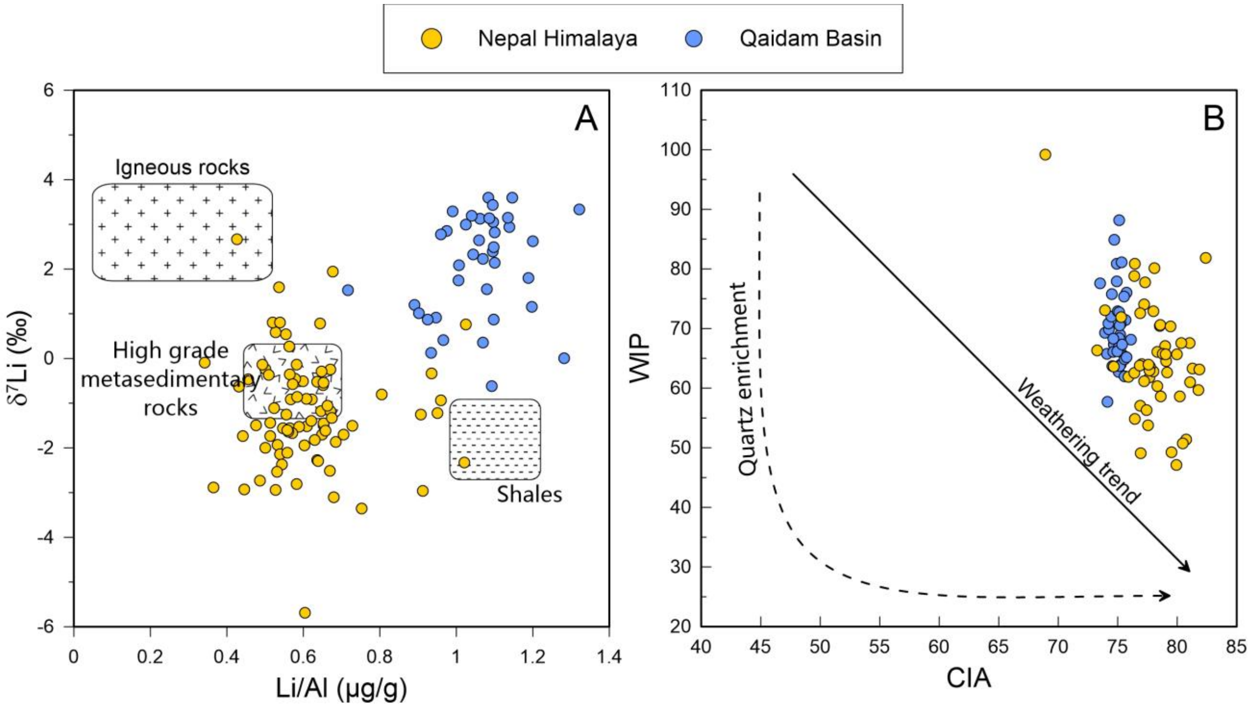

Fig. S7.
The plot of δ^7Li vs. Li/Al **(A)**, and WIP vs. CIA **(B)**. The endmembers of igneous rocks, shales, high grade metasedimentary rocks are based on ref. *43*.

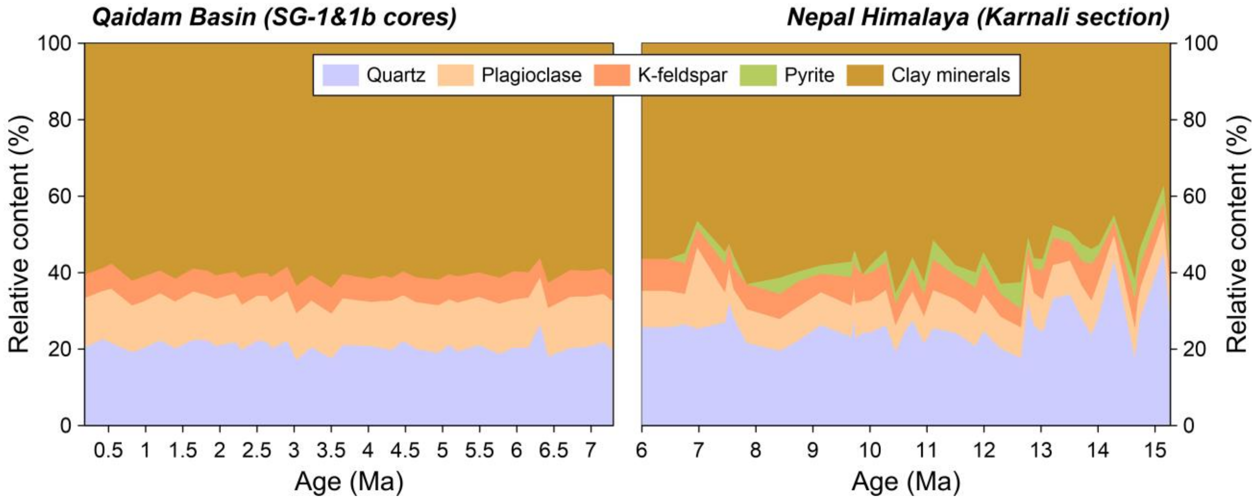

Fig. S8.

XRD diffractograms of <2μm fraction of sediment from the SG-1 and SG-1b cores and the Karnali section. The results show that non-clay minerals account for >40% in the <2μm fraction.

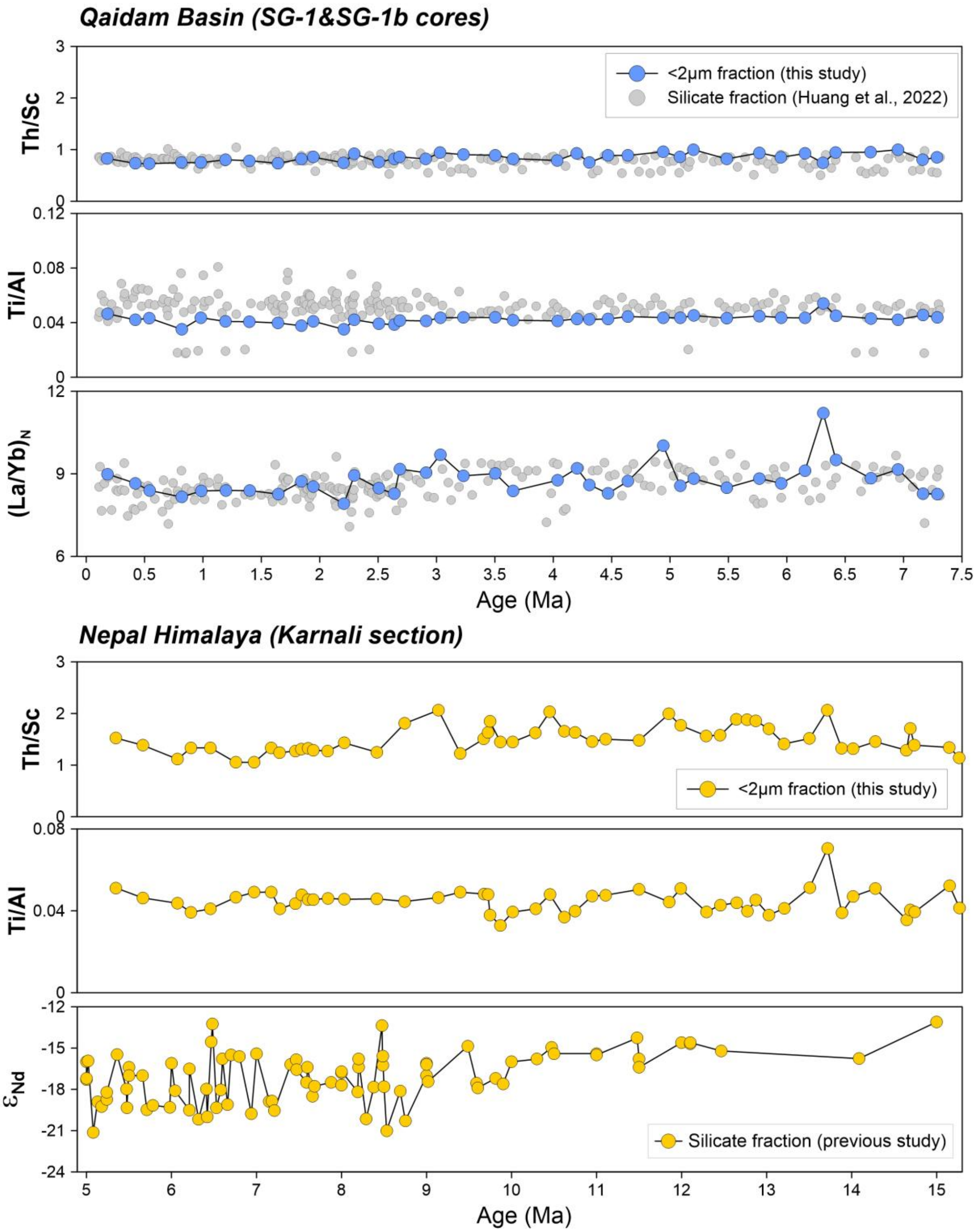

Fig. S9.

Provenance change in the Qaidam Basin and Nepal Himalaya. The data of silicate fraction of the SG-1 and SG-1b core sediments are from ref. *100*. The data of Nd isotopes are from refs. *47-50*.

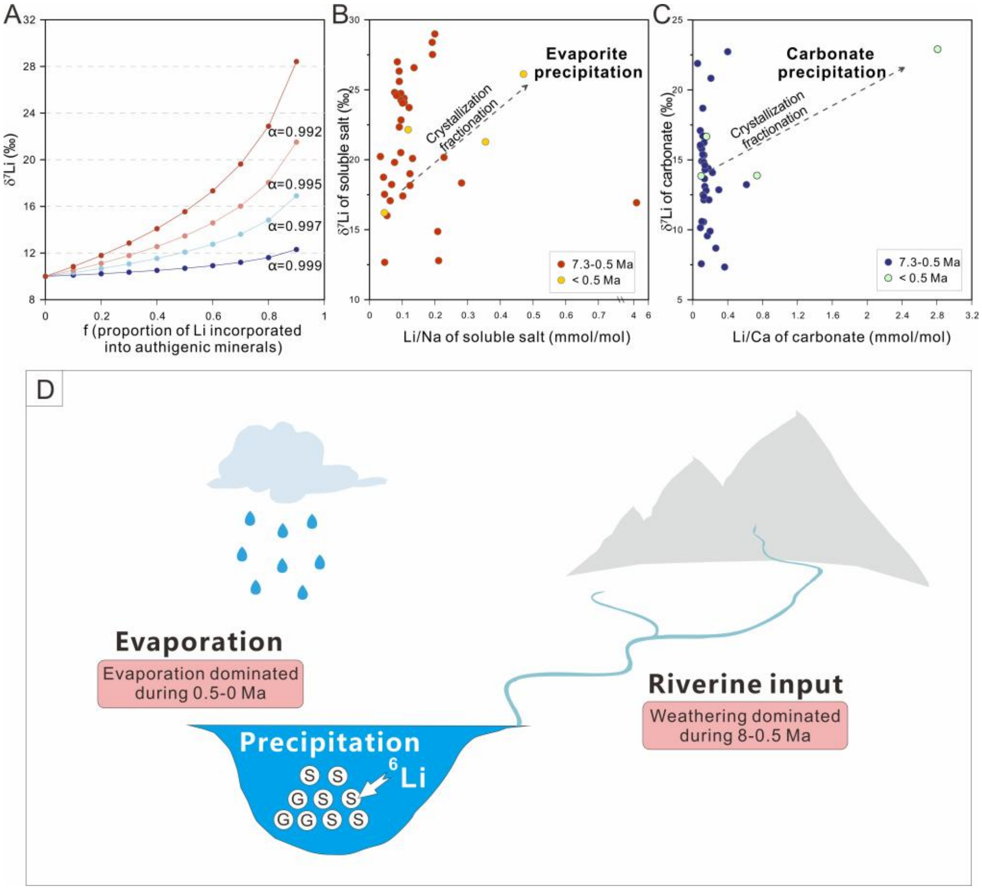

Fig. S10.

The paleo lake water δ^7Li record regulated by lake evaporation-driven fractionation during 0.5-0 Ma and catchment silicate weathering during 7.3–0.5 Ma. **(A)** Modeling the δ^7Li evolution with proportion of Li incorporated into authigenic minerals under different fractionation factors (α), **(B)** δ^7Li values vs. Li/Na ratios of soluble salt phase in the SG-1 and SG-1b cores, **(C)** δ^7Li values vs. Li/Ca ratios of carbonate phase in the SG-1 and SG-1b cores, **(D)** Schematic diagram of source to sink process of lithium isotope in lake at a catchment scale. Different fractionation factors were determined according to the fractionation values of water and minerals observed in the evaporation experiment (*35-41*), and the middle value was selected to simulate more comprehensively.

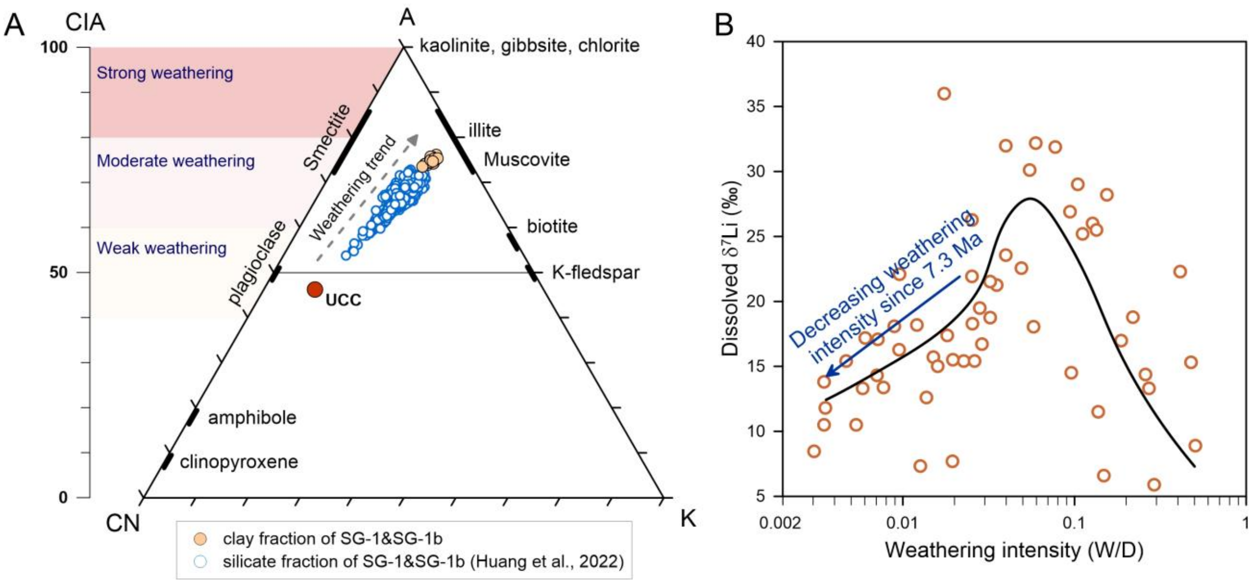

Fig. S11.

A-CN-K ternary diagrams of the SG-1 and SG-1b cores sediments **(A)** and correlation between dissolved Li isotopes and weathering intensity (*6*) **(B)**.

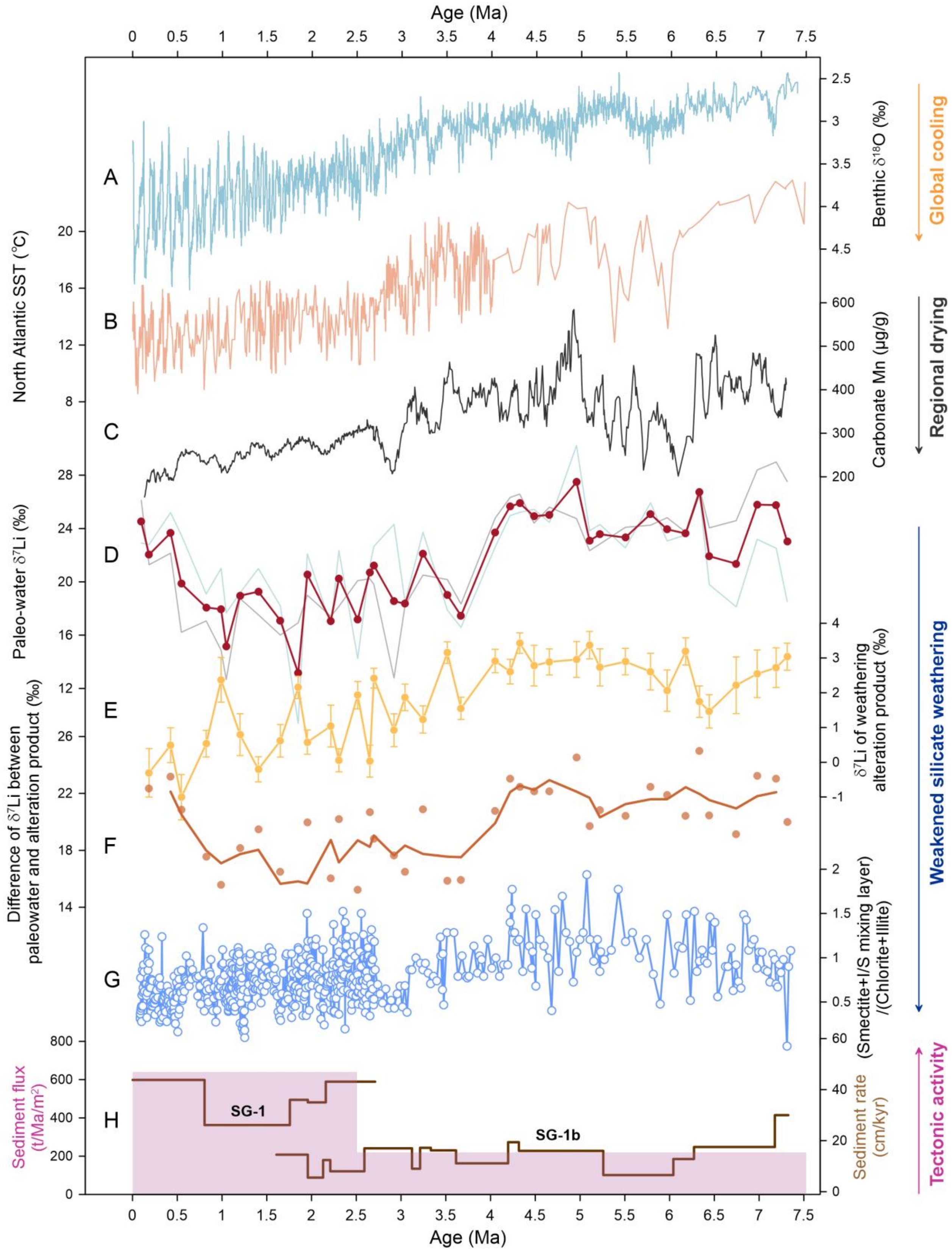
Age (Ma)
A
B
C
D
E
F
G
H
North Atlantic SST (°C)
Paleo-water δ7Li (‰)
Difference of δ7Li between paleowater and alteration product (‰)
Sediment flux (t/Ma/m2)
Benthic δ18O (‰)
Carbonate Mn (μg/g)
δ7Li of weathering alteration product (‰)
(Smectite+I/S mixing layer) /(Chlorite+Illite)
Sediment rate (cm/kyr)
Global cooling
Regional drying
Weakened silicate weathering
Tectonic activity
SG-1
SG-1b

Fig. S12.

Mult-phase Li isotopic records from the Qaidam Basin, compared with other records. **(A)** Global marine benthic foraminiferal $\delta^{18}O$ record (*52*), **(B)** North Atlantic surface sea temperature (SST) (*53*), **(C)** regional drying of the Qaidam Basin based on the carbonate Mn content of the SG-1 and SG-1b cores (*25*), **(D)** paleo-water $\delta^{7}Li$ records over the past 7.3 Myr in the Qaidam Basin based on average value of carbonate and soluble salt fraction, **(E)** $\delta^{7}Li$ record of weathering alteration product (yellow line) over the past 7.3 Myr in the Qaidam Basin with the uncertainty of reconstruction (2SE), **(F)** the $\delta^{7}Li$ difference between paleo-water and weathering alteration product, **(G)** silicate weathering intensity, (Smectite+I/S mixing layer)/(Chlorite+Illite) ratios, of the Qaidam Basin based on clay mineral (*95-96*), **(H)** sediment flux of the Qaidam Basin (purple shade) (*97*) and sediment rate of the SG-1 and SG-1b cores (brown lines) (*72-73*).

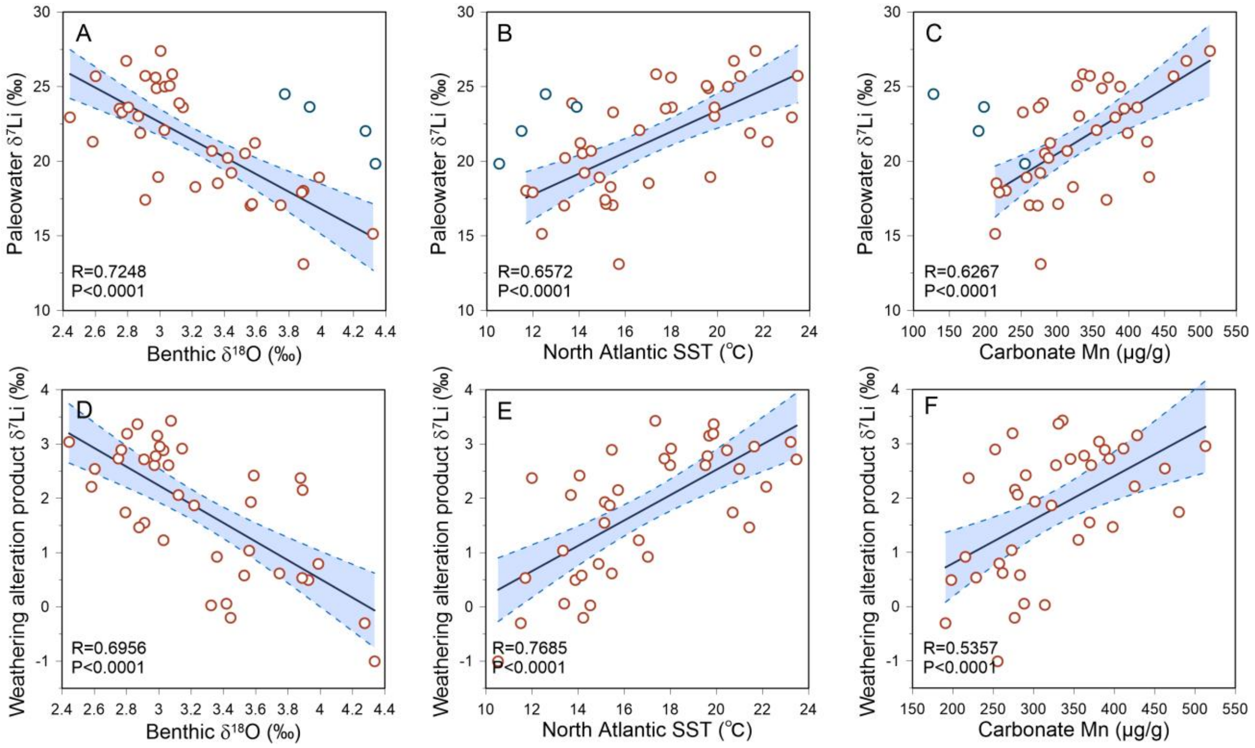

Fig. S13.

Correlation of δ^7Li of paleowater and weathering alteration product with global/regional climate records at 7.3–0.5 Ma. Relationships between paleowater δ^7Li and benthic $\delta^{18}O$ (**A**), North Atlantic SST (**B**), and carbonate Mn content (**C**) from the Qaidam Basin. Relationships between weathering alteration product δ^7Li and benthic $\delta^{18}O$ (**D**), North Atlantic SST (**E**), and carbonate Mn content (**F**) from the Qaidam Basin. The shaded area represents the 95% confidence interval of the linear fit. The corresponding datapoints for global/regional climate records are obtained by linear interpolation.

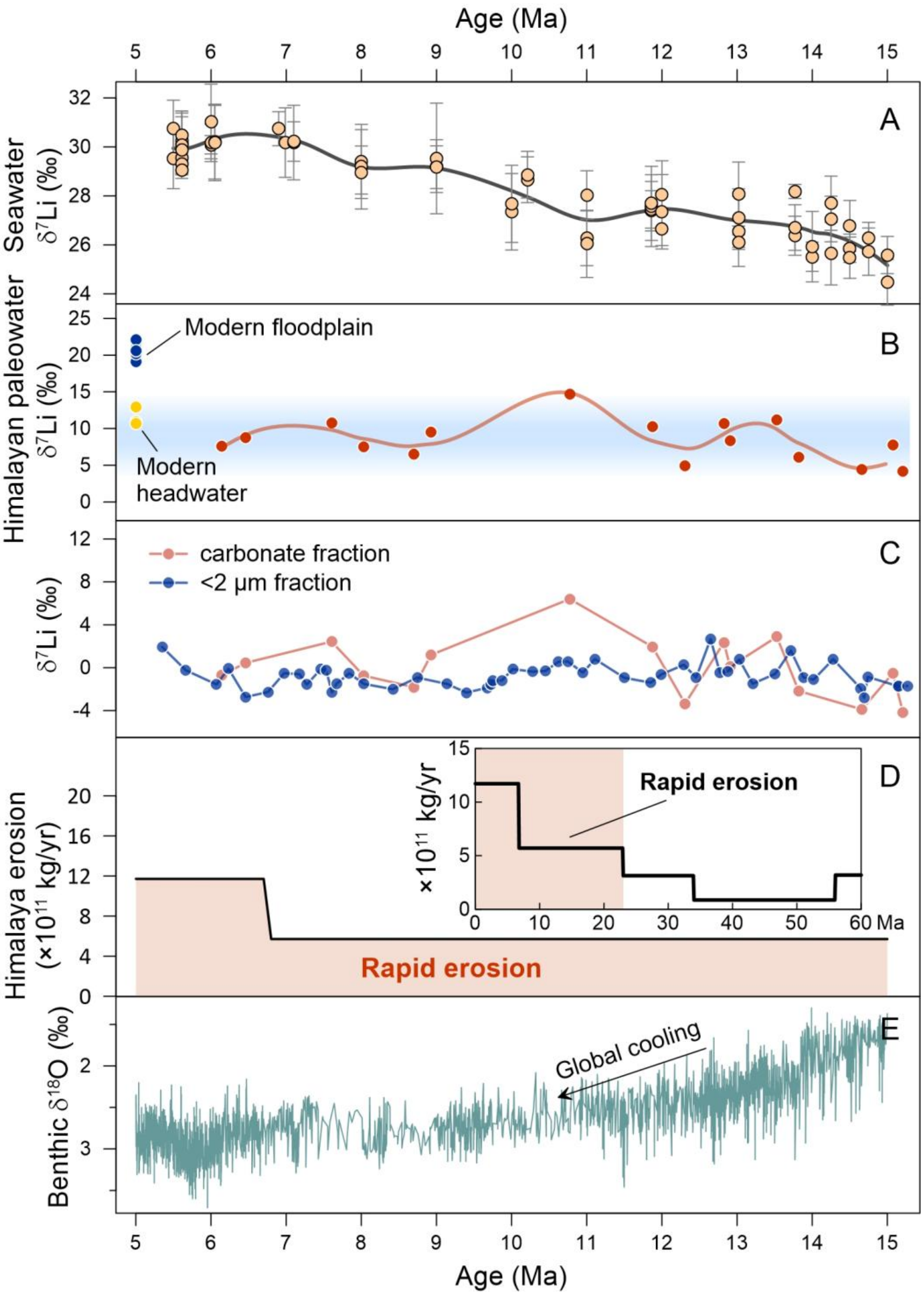

Fig. S14.

Mult-phase Li isotopic records from the Nepal Himalaya, compared with other records. **(A)** Seawater δ^7Li record with two standard errors (2SE) (*7*), **(B)** paleowater δ^7Li records in the Nepal Himalaya based on carbonate fraction, **(C)** δ^7Li record of carbonate and <2 μm fraction in the Nepal Himalaya, **(D)** sediment flux of the Bengal Fan (*65*), (E) global marine benthic foraminiferal δ^{18}O record (*52*).
