# Supplementary material for: Decoupling of Neogene seawater lithium isotopes from uplift-driven weathering
Source: Nat Commun. 2026 May 15;17:6524. doi: 10.1038/s41467-026-71407-x (PMC13376809; doi:10.1038/s41467-026-71407-x)
Supplement: Supplementary file 2 — Description of Additional Supplementary Files [file 41467_2026_71407_MOESM2_ESM.pdf]

Description of Additional Supplementary Files:

Supplementary Data 1: Element concentrations and Li isotopes of different phases and mineral compositions of SG-1 and SG-1b core sediments from the Qaidam Basin.

Supplementary Data 2: Element concentrations and Li isotopes of different phases and mineral compositions of Karnali section sediments from the Nepal Himalaya.

Supplementary Data 3: Comparison between δ7Li value of the standard sample in this study and the reference value reported in the literature.

Supplementary Data 4: Reconstructed Li isotopes of paleo water and weathering alteration product in the Qaidam Basin and Nepal Himalaya.

Supplementary Data 5: Statistical values of Li content and δ7Li of different minerals
