# Supplementary material for: Decoupling of Neogene seawater lithium isotopes from uplift-driven weathering
Source: Nat Commun. 2026 May 15;17:6524. doi: 10.1038/s41467-026-71407-x (PMC13376809; doi:10.1038/s41467-026-71407-x)
Supplement: Supplementary file 4 — Transparent Peer Review file [file 41467_2026_71407_MOESM4_ESM.pdf]

nature portfolio

Peer Review File

Decoupling of Neogene Seawater Lithium Isotopes from Uplift-driven Weathering

Corresponding Author: Dr Yibo Yang

This file contains all reviewer reports in order by version, followed by all author rebuttals in order by version.

Version 0:

Reviewer comments:

Reviewer #1

(Remarks to the Author)
This is an interesting contribution relative to the evolution of continental weathering in the Tibetan plateau area over the last 7 million years. To do so, the authors rely on several isotopic records measured in the sediment accumulated in the Qaidam basin, the most important being the lithium isotopic composition. The innovative aspect of the study is the reconstruction of the change in the d7Li of the rivers for the Tibetan plateau as a function of time, using the lithium isotopic composition of carbonates precipitated in the basin. Inferring the river d7Li from continental proxies is a difficult task, given the amount of processes at play and able to hide the true riverine signal. I'm not a specialist of the sediment analysis, but it seems to me that this work has been done with care.

Then, Yang et al. utilize a numerical model (in fact 2 differential equations, one for the lithium content of the global ocean, and one for its lithium isotopic composition). These mass and isotopic budget equations are partitioned between two components: the Himalayan/Tibetan contribution and the rest of the world. From the present day budget, assign the isotopic signature they calculated from their sedimentary data to the Himalayan/Tibetan contribution. Several tests are performed by fixing or varying the other parameters.

I'm not sure that I understood the outcome of the modeling results. This model section is confusing and not clearly written. Their main conclusion is that if the Li isotopic composition of the rivers from the Tibetan/Himalayan plateau declines through time, then the seawater isotopic composition will also decline, all other factors being held at a constant value. Given that the seawater isotopic composition is rising, Yang et al. concludes the silicate weathering of the Tibetan/Himalayan region cannot be the cause of the global cooling since 8 Ma. I think that this is wrong. The error lies in the use of an isotopic model alone, with no coupling with the carbon cycle. Vigier and Goddéris (2014) have show that it is possible to make rising the Li isotopic composition of seawater with a rise in thre silicate weathering flux, even if the global riverine isotopic composition decreases. And this is made possible by accounting for the coupling with the carbon cycle. All the fluxes in the Li budget are not independent, and some of them are co-varying because of the existence of feedback loops in the carbon cycle.

Overall, the data set presented here is crucial for our understanding of the climate evolution over the end of the Cenozoic. However, I think that the conclusions are not supported by their model analysis. I would send this paper to a specialized journal.

I hope that my comments will help.

Reviewer #2

(Remarks to the Author)
The manuscript entitled "Limited Contribution of Continental Weathering to Late Cenozoic Seawater Li Isotope Evolution" presents a systematic investigation of Li isotopic compositions in drill cores from the Qaidam Basin, northeastern Tibetan Plateau. Based on the water-soluble and carbonate phases, the authors reconstructed paleowater δ7Li values and observed a decrease of ~10‰ over the past ~7 Myr. This trend contrasts with the slight increase in seawater δ7Li values during the same period. Using a box model, the authors conclude that the evolution of seawater Li isotopes during the late Cenozoic cannot be primarily attributed to riverine input from the Tibetan Plateau. The findings of this study may contribute to

improving our understanding of Cenozoic weathering. The manuscript is well-written, but I believe the authors could further refine their interpretations. I would recommend a decision of moderate revision.

My major concerns lie in sample representativeness and data interpretations:

1, The authors appear to have overestimated the riverine Li flux from the Tibetan Plateau (Line 26). The Pearl River and Hong He do not originate from the Tibetan Plateau. The dissolved Li concentration in the Mekong River was cited from its upper stream (referred to as the Lancang River; Li et al., 2023). However, the water discharge of the Lancang River is only about 64 km3/yr, compared to 550 km3/yr for the entire Mekong River. A more conservative calculation would be preferable. Furthermore, the drill cores are located in a hydrologically closed lake (Line 75), meaning the lake water does not flow into the ocean. How can these samples be used to investigate the influence of Tibetan Plateau weathering on seawater Li isotopic evolution (Lines 309-311)? Is it possible that the Li isotopic variation simply reflects lake evolution (e.g., changes in the sedimentary environment) rather than continental weathering?

2, Evidences for excluding contamination and diagenetic effects are insufficient. For instance, in lines 83 and 114, the term "non-carbonate and detrital contamination" was used. The authors only examined the impacts of detrital contamination. In my opinion, the potential release of Li from Fe-Mn oxyhydroxides should also be considered; otherwise, the terms "non-carbonate" and "detrital" are repeated. Please also evaluate the potential influence of gypsum on the Li isotopic composition of the carbonate phases, considering that acetic acid (HAc) used in extraction might dissolve gypsum and release its Li. Regarding the diagenetic effects, in lines 94–96, a higher content of aragonite relative to calcite and dolomite is not sufficient evidence to exclude diagenetic effects. How can the higher-than-normal Li/(Mg+Ca) ratios (52–693 μmol/mol) in the carbonate fraction be explained? In my view, the potential influence of diagenesis on both the water-soluble phase and carbonate was not thoroughly investigated. Changes in the sedimentary environment may affect pore water evolution, which could indirectly alter the Li isotopic compositions of halite and carbonate.

3, Weathering intensity and weathering rate are distinct concepts, and they are influenced differently by climate and tectonic factors. I believe the authors have sometimes misused these terms. For instance, in tectonically active regions, weathering rate is often sensitive to climate changes (i.e., weathering-limited regimes). Generally, Li isotopic composition reflects weathering intensity. In this context, I disagree with the explanation in Lines 261-264, which suggests a negligible influence of tectonic activities. Additionally, the sedimentation rates of SG-1 and SG-1b may not accurately represent the erosion rate of the entire Tibetan Plateau, as they are from different sedimentary environments (depositional center vs. Jianshan Anticline). It would be preferable to include regional erosion rate data. Similarly, was the cited temperature drop of 5–8 °C (Line 239) derived from terrestrial or deep-sea records? I would prefer to see regional temperature reconstructions. Moreover, the global mean riverine Li isotopic composition has been updated recently. Please consult the reference by Läuchli et al. (2025). The value of δ7Liriv_other in Table 2 is 25.6‰, which may be unreasonably high and could affect your model. Fourth, how was the W/D (weathering flux/denudation flux) ratio over the past 8 Ma derived (Figure 3)? The riverine Li flux from the Tibetan Plateau is important for explaining the slight increase in seawater δ7Li. Perhaps an increase in this flux offset the decrease in riverine δ7Li. Fifth, how was Friv_other set during the modelling? Global weathering flux is proposed to have remained constant over the Neogene (Caves Rugenstein et al., 2019). If you did not adopt this assumption, please provide justifications.

Other comments:

Lines 90–91, references 29–30 only discuss the influence of diagenetic processes on carbonate minerals. Please add appropriate references that address silicate minerals.

Line 132, the scale in Figure S4 is too large to discern details of the Mg/Ca variations.

Line 134, please carefully verify the fractionation factor of "-8.5±2‰ for inorganic calcite". This value is not commonly used in the literature.

Line 140, during halite precipitation, light 6Li is preferentially incorporated into the crystal structure. However, the majority of Li in halite is stored in non-fractionated fluid inclusions, which is the rationale for effectively setting the fractionation factor to 0‰. The authors should clarify this point to avoid confusion with the general statement in Line 205 that "light Li isotopes (6Li) preferentially partition into precipitating carbonate and evaporite minerals."

Line 152, the 87Sr/86Sr ratios of the carbonate phase were not measured. Could the authors explain why? This data seems important for a comprehensive interpretation. Please seperatively display the 87Sr/86Sr ratios somewhere as what you did in Figure S7.

Line 156, please consider adding relevant references that discuss Li isotopes in clay-sized fractions to support the methodology or interpretation here.

Line 171, the claim that "the impact of recycled sedimentary materials is minor" is unclear. In Figure S10A, the data points for clay-sized samples predominantly fall within the range of the shale endmember, which seems to suggest a significant influence. Could this be explained further?

Line 192, please provide a brief explanation of why these specified proxies are effective for distinguishing between different sources. Is the distinction primarily based on grain size, geochemical composition, or other factors?

Line 203, this section requires clarification. First, I agree with the statement that "light Li isotopes (6Li) preferentially partition into precipitating carbonate and evaporite minerals" (Line 205). However, carbonate and evaporite are not Li-rich minerals. The resulting flux is likely negligible compared to the total Li reservoir in the lake, making an observable increase in the water's δ7Li value improbable. Therefore, it seems unrealistic that 90% of Li could be incorporated into authigenic minerals if these are carbonate and evaporite. Second, the choice of fractionation factors (0.999, 0.997, 0.995, 0.992) needs justification. Which specific minerals do these factors represent? Third, the salt layer is noted above ~414 m (~1.2 Ma), yet 0.5 Ma is used as a boundary. What is the reason for this discrepancy? Could the anomalous values be related to gypsum? Generally, δ7Li values of gypsum are higher than contemporaneous halite.

Line 384: The description "the fine-grained lacustrine sediments… were selected" is ambiguous. Does this indicate that bulk sediments were manually chosen based on their fine-grained nature, or were they mechanically sieved to a specific grain size range? Please clarify the selection process.

Line 433: The reported δ7Li value for the BHVO-2 reference material appears lower than the commonly accepted consensus value in the literature. Please verify this measurement or provide a justification for the discrepancy.

Reviewer #3

(Remarks to the Author)
Yang et al present a new d7Li record from the late Neogene Qaidam Basin that shows declining d7Li at the same time at the marine record of d7Li increases slightly or is stable. They use scaling relationships to scale this record to the entire Himalayan-Tibetan Plateau system and argue that the declining d7Li indicates a decrease in weathering from tectonically active areas and that therefore these areas could not have driven Cenozoic cooling.

This was a fascinating paper to read, and I think the presentation of the paleo-d7Li record is outstanding. The authors do a very thorough job of ruling out diagenesis and contamination, and I'm impressed that this decline in d7Li is visible in two different archives—salt and carbonates. This makes this record especially robust.

With that said, I don't think these findings are as exciting as the authors claim. I'm very skeptical of the estimates that scale this result to the entire Tibetan Plateau-Himalayan system, and the decline in d7Li is the expected response for tectonically active areas that are driving cooling in response to a driver such as uplift. So, to me, there is no need to rethink anything… this is simply confirming the response on a local-scale. I don't know if this is a fixable problem, and I leave it to the editor to determine if this is major revisions or simply a rejection of the paper.

The scaling of this finding to the entire Tibetan Plateau-Himalayan system is fairly suspect. The Qaidam Basin has been a closed basin for much of the Neogene (if not longer; Carroll et al. (2010)) and certainly for the entire length of the core presented. Almost by definition, a closed basin receives little moisture; in contrast, most of the weathering-action in the Himalaya-Tibetan Plateau (HTP) system takes place in areas that are today some of the wettest places on Earth and are influenced by the two big Asian monsoonal systems. Thus, climatically, the Qaidam Basin is not at all representative of much of the Himalayan-Tibetan Plateau system. How then do these very different climate regimes influence the d7Li signature of the various rivers draining the HTP? There is very little evidence presented to suggest that the Qaidam Basin should even serve as a representative of the rest of the HTP. While the record that Yang et al. present is undoubtedly very interesting, it seems unlikely to be anything more than a local record in a fairly arid location.

Further, the authors present this record as a surprising result that should cause us to question the role of tectonically active ranges in the global weathering engine and in global cooling. I agree with the authors that a decrease in d7Li indicates a decline in weathering intensity, but this does not mean a decrease in the weathering flux as the authors often imply. There isn't any way that the authors can deduce this except through other methods not presented in the paper by, for example, using Be isotopes or estimating erosion rates to back-out the weathering flux implied by the weathering intensity. Instead, the decline in weathering intensity (and decrease in d7Li, given the position of these catchments relative to the Dellinger et al. (2015) curve (Figure 3)) is what is predicted from a global carbon cycle-climate driven by an increase in erosion. That is, the global weathering flux is determined by mass balance to equal the volcanic flux. As long as this isn't changing—and if erosion/uplift is increasing—then weathering intensity has to go done. How much it goes down (and if it goes down at all) in tectonically active areas is probably a function of lithology, rate of uplift increase, climate, etc., but this is poorly known. Regardless, all else being equal, most places on the planet will likely see a decrease in weathering intensity. So, while this is a very neat and robust record, I'm not convinced that it upends our understanding of the role of mountain ranges in weathering and global cooling.

References cited in review

Carroll, A.R., Graham, S.A., Smith, M.E., 2010. Walled sedimentary basins of China. Basin Research 22, 17–32. https://doi.org/10.1111/j.1365-2117.2009.00458.x
Dellinger, M., Gaillardet, J., Bouchez, J., Calmels, D., Louvat, P., Dosseto, A., Gorge, C., Alanoca, L., Maurice, L., 2015. Riverine Li isotope fractionation in the Amazon River basin controlled by the weathering regimes. Geochimica et Cosmochimica Acta 164, 71–93. https://doi.org/10.1016/j.gca.2015.04.042

Version 1:

Reviewer comments:

Reviewer #1

(Remarks to the Author)
The authors have undertaken a monumental revision. Even the title and the author list have changed. Entire sections of the original text were removed and replaced with a new analysis, particularly regarding the modeling component. I must admit, I am somewhat perplexed; the revision is so extensive that we are essentially looking at a different paper. I would jokingly suggest that this level of rewriting almost warrants including the reviewers in the author list. All kidding aside, it does raise questions about the value of the initially submitted study, since—data aside—nearly everything else was incorrect.

That said, in their response to the reviewers, the authors mention they will be using the lithium-carbon coupled model from Vigier and Goddéris (2014). The primary contribution of V&G was the inclusion of the carbon cycle. I should note that the authors addressed my comments on this modeling point by integrating a discussion on the coupling of Li cycles and its isotopes with the carbon cycle. Overall, the manuscript has been significantly improved. I have no specific further comments; I believe this work, based on high-quality data and a simple yet coherent modeling approach, deserves publication. However, the complete overhaul of the text remains striking. I naturally leave the final decision to the Editor.

Reviewer #2

(Remarks to the Author)
This revised manuscript has undergone substantial modifications that have effectively addressed my concerns. I think one additional minor issue is still the modeling.

For Scenarios 1 (erosion flux-based Li flux) and 3 (weathering intensity-based Li flux), the authors should consider whether the drainage patterns of the northern and southern Tibetan Plateau remained consistent throughout the Neogene. Any changes in these patterns could alter the division between the northern and southern regions. To my knowledge, significant drainage reorganizations occurred during this period. For instance, the Yellow River formed relatively recently (e.g., ~1.25 Ma; Wang et al., 2022), and the modern Irrawaddy River originated in the late Miocene–Pliocene (~7 Ma; Jonell et al., 2022).

The modern riverine Li flux has been updated recently. That means the value of 23‰ in Table 2 is no longer accurate. Please refer to the references Läuchli et al. (2025) and Yang et al. (2025). Also, please correct the typo in Table 2 (3.65 × 16 mol for seawater Li reservoir)

Reviewer #3

(Remarks to the Author)
In this revised manuscript, Yang and co-authors add an additional record from the Siwaliks to estimate d7Li across a longer timeframe. They find that the Siwalik record does not change much over the Neogene (or, more precisely, there is a fair amount of variability, but no directional change) and the reconstructed river values are themselves fairly low. They conclude that weathering in the Himalayas cannot be a source of rising seawater d7Li and that other fluxes (either from the seafloor or from other continental areas) must be responsible for the rise of seawater d7Li.

I found this version of the manuscript to be substantially better than the previous version and addresses the two central critiques of my previous review. First, I was skeptical that the d7Li record from the Qaidam Basin could be scaled to the entire Himalaya-Tibetan Plateau system. In some sense, this concern was correct, but the new record from the Siwaliks is fascinating and also novel. I know of few other types of these records anyway, particularly from such an active orogen.

My second critique regarded the authors interpretation of how declining d7Li in the Qaidam Basin should be interpreted in terms of weathering flux and weathering intensity. Again, here, the authors have substantially revised their interpretation and now focus their interpretation on what these two records mean for weathering intensity in the Tibetan Plateau-Himalaya region and how this impacts the seawater d7Li budget. I think this is a reasonable way of interpreting the data, and it certainly sets up a number of questions that other researchers can follow up on.

My only comment regards the discussion of floodplain d7Li. The authors have put together an interesting compilation of floodplain d7Li, but then argue that, though d7Li tends to increase across floodplains, this cannot, in a sense, cancel out the signal of declining or constant d7Li that they find in their sections. I disagree, and I think it's hard for the authors to know whether this 6-10‰ increase in d7Li downstream has been constant or has, actually, increased through time. If the relationship of d7Li downstream has changed through time, then it could be the changing role of floodplain weathering that partially mutes any decreasing (or constant) d7Li signal recorded in these sections.

Open Access This Peer Review File is licensed under a Creative Commons Attribution 4.0 International License, which permits use, sharing, adaptation, distribution and reproduction in any medium or format, as long as you give appropriate credit to the original author(s) and the source, provide a link to the Creative Commons license, and indicate if changes were made.
In cases where reviewers are anonymous, credit should be given to 'Anonymous Referee' and the source.
The images or other third party material in this Peer Review File are included in the article’s Creative Commons license, unless indicated otherwise in a credit line to the material. If material is not included in the article’s Creative Commons license and your intended use is not permitted by statutory regulation or exceeds the permitted use, you will need to obtain permission directly from the copyright holder.
To view a copy of this license, visit https://creativecommons.org/licenses/by/4.0/

We would like to extend our sincere thanks to the three reviewers for their thorough and constructive comments, which have been invaluable in improving our manuscript. We have carefully addressed all the points raised during the revision process. Specifically, in response to the comments concerning the upscaling, the broader implications for our records, and the impact of riverine lithium flux variations, we have incorporated a completely new Neogene lithium isotope record from the southern Tibetan Plateau and supplemented four scenario simulations constraining variations in riverine lithium fluxes in this model. We believe these additions effectively address the issues highlighted by the reviewers. Below, these comments are in black and our replies are in blue. Line numbers refer to **the revised CLEAN manuscript** because the tracked version includes too many modifications to be recognized. The underlined text refers to that in the Main text.

Response to Reviewer #1:

Reviewer #1 (Remarks to the Author):

This is an interesting contribution relative to the evolution of continental weathering in the Tibetan plateau area over the last 7 million years. To do so, the authors rely on several isotopic records measured in the sediment accumulated in the Qaidam basin, the most important being the lithium isotopic composition. The innovative aspect of the study is the reconstruction of the change in the d7Li of the rivers for the Tibetan plateau as a function of time, using the lithium isotopic composition of carbonates precipitated in the basin. Inferring the river d7Li from continental proxies is a difficult task, given the amount of processes at play and able to hide the true riverine signal. I'm not a specialist of the sediment analysis, but it seems to me that this work has been done with care.

Reply: We really appreciate the reviewer's recognition of our data and constructive comments on our manuscript. We have carefully considered the suggestions and amended the relevant parts in the manuscript. The review comments prompted us to further seriously consider the impact of Li flux changes and how to effectively constrain such changes, which significantly strengthened this paper.

Then, Yang et al. utilize a numerical model (in fact 2 differential equations, one for the lithium content of the global ocean, and one for its lithium isotopic composition). These mass and isotopic budget equations are partitioned between two components: the Himalayan/Tibetan contribution and the rest of the world. From the present day budget, assign the isotopic signature they calculated from their sedimentary data to the Himalayan/Tibetan contribution. Several tests are performed by fixing or varying the other parameters.

I'm not sure that I understood the outcome of the modeling results. This model section is confusing and not clearly written. Their main conclusion is that if the Li isotopic composition of the rivers from the Tibetan/Himalayan plateau declines through time, then the seawater isotopic composition will also decline, all other factors being held at a constant value. Given that the seawater isotopic composition is rising, Yang et al. concludes the silicate weathering of the Tibetan/Himalayan region cannot be the cause of the global cooling since 8 Ma. I think that this is wrong. The error lies in the use of an isotopic model alone, with no coupling with the carbon cycle. Vigier and Goddéris (2014)

have show that it is possible to make rising the Li isotopic composition of seawater with a rise in the silicate weathering flux, even if the global riverine isotopic composition decreases. And this is made possible by accounting for the coupling with the carbon cycle. All the fluxes in the Li budget are not independent, and some of them are co-varying because of the existence of feedback loops in the carbon cycle.

Reply: We appreciate the reviewer's constructive comments on our manuscript. We have carefully considered all suggestions and revised the relevant sections accordingly. The reviewer's insights have substantially strengthened the paper and prompted us to further refine the clarity and robustness of several key arguments. In particular, the model framework (Vigier and Goddéris, 2014) offer a valuable perspective on coupling Li fluxes with carbon cycle processes, which we explicitly adopted in the revised manuscript.

As noted by the reviewer, the potential contribution of an increasing Li flux to the rise in seawater δ^7Li—especially when considered within the context of carbon cycle dynamics—warrants a more rigorous and quantitative assessment. This is now feasible in the revised version, as we have imposed more refined and diversified constraints on Li flux inputs in the model while explicitly accounting for their coupling with carbon consumption during silicate weathering. To better constrain the evolution of Li export from the Tibetan Plateau, we employed four complementary and independent approaches.

1. Erosion-based scaling: We assumed that past Li export varied proportionally with Neogene changes in the northern and southern plateau erosion flux (Métivier et al., 1999) (Fig. 5B).
2. Silicate weathering reconstruction: We coupled the reconstructed carbon consumption fluxes by silicate weathering (Clift et al., 2024) based on the assumption that the simultaneous loss of major cation (e.g., Ca, Na, Mg and K) and Li during silicate weathering rates, which is the proposed approach of Vigier and Goddéris (2014), as mentioned by the reviewer (Fig. 5C).
3. W/D–Li flux relationship: We applied empirically derived relationships among Li flux, δ^7Li, and the weathering-to-denudation (W/D) ratio from modern river systems (Pogge von Strandmann et al., 2021) to infer past Li flux from isotopic signatures (Fig. 5D).
4. Constant weathering scenario: We incorporated constant weathering fluxes constrained by Be isotope records (Willenbring & von Blanckenburg, 2010) as well as the stable ^{10}Be-derived erosion flux during the past 6 Myr (Lenard et al., 2020) and steady state of erosion regimes during the past 13 Myr (Galy et al., 2010; Huyghe et al., 2020), in the Himalay (Fig. 5E).

We tested these four scenarios independently. Collectively, they encompass the full range of current scientific understanding regarding Neogene weathering-driven Li fluxes on the Tibetan Plateau. Our model results show that, regardless of whether the riverine Li flux from the Tibetan Plateau increased or remained constant over the past 15 Myr, the resulting plateau-derived Li input to the ocean produces only a relatively flat trend in seawater δ^7Li (Fig. 5). These findings indicate that riverine inputs from the Tibetan Plateau-despite rapid erosion and global cooling—are unlikely to account for the observed increase in seawater δ^7Li over at least the past 15 Myr.

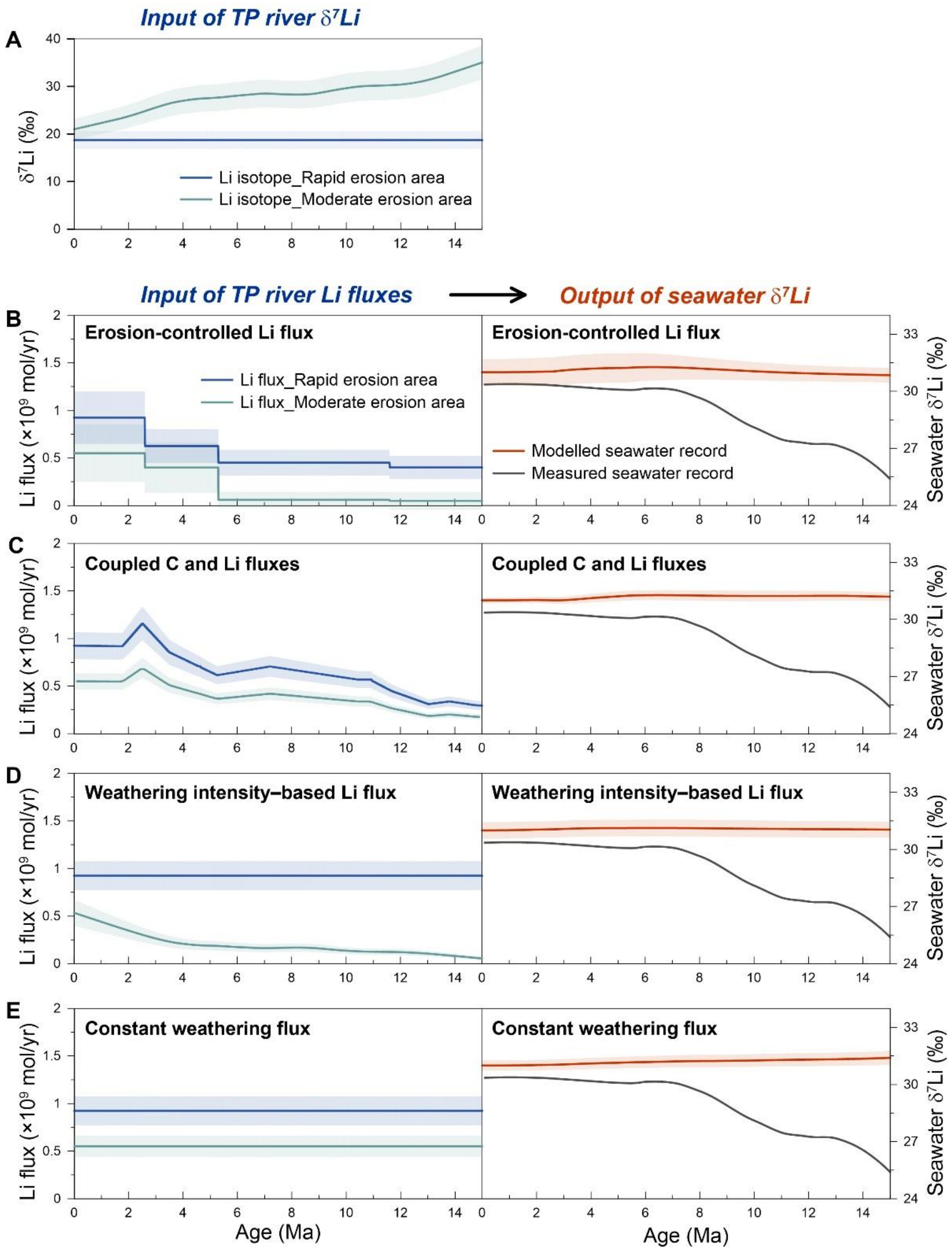

Fig. 5. Dynamic simulation of seawater Li isotope changes under reconstructed Li fluxes and δ^7Li values from the Tibetan Plateau (TP). (A) The evolution of δ^7Liriv of the rapid erosion area (blue line) and moderate erosion area (green line) of the Tibetan Plateau. (B–E) The evolution of input river Li fluxes and model result of seawater δ^7Li, (B) based on sediment accumulation rates of surrounding basins (63) (Scenario 1), (C) coupled with C and Li fluxes (63) (Scenario 2), (D) based on correlation between Li yield, δ^7Li, and W/D (64) (Scenario 3), (E) based on constant weathering flux (based on Be isotope records) (65) (Scenario 4). The measured seawater δ^7Li curve is from ref. (7). See methods for details of the four scenarios. None of the four scenarios can reproduce the rise in seawater δ^7Li since 15 Ma.

We further redraw a new figure 5 (shown in the above) and elaborate this issue in Lines 300-350 and as "It is essential to constrain both the Li flux and isotopic composition input from the Tibetan Plateau. Reconstructing past variations in riverine Li flux from the Tibetan Plateau remains highly challenging due to the absence of direct reconstructions of ancient river discharge and Li concentrations. We first quantified the modern riverine Li fluxes across the plateau through statistical analyses (Table 1). The results indicate that major rivers originating from the Tibetan Plateau collectively export a Li flux of approximately ~1.5×10^9 mol/yr to the ocean (accounting for ~18% of global river input) (Table 1). We then employed four scenarios to constrain the Li flux evolution of the Tibetan Plateau over the past 15 Myr (see Methods). First, the past Li flux varied proportionally with variations in the Neogene erosion flux of the northern and southern plateau (Fig. 1B) (*63*). Second, the atmospheric CO_2 consumption flux by silicate weathering of the Tibetan Plateau (*79*) constrain the dissolved Li flux based on the C-Li coupling assumption that Li is congruently released, similar to the way base cations (Na, K, Ca and Mg) are depleted during silicate weathering (*13*) (Fig. 5C). Third, the Li flux through time is constrained by the empirically-derived relationship with δ^7Li, the weathering-to-denudation (W/D) ratio from modern rivers (*64*), and our measured δ^7Li signatures (Fig. 5D). Fourth, temporal changes in Li flux is linked to the constant Tibetan Plateau erosion flux based on based on constant exhumation and erosion rates (*47, 58, 65*) (Fig. 5E). Subsequently, we tested the effect of these four different scenarios separately in our Li mass balance model and these four scenarios essentially encompass the current understanding of the weathering-induced lithium flux on the Tibetan Plateau during the Neogene period.

Using our reconstructed record, we can further constrain the variations in overall lithium isotope composition related to plateau weathering. To differentiate between regional weathering patterns, we used the sediment flux from Central Asia and the East China Sea platform to estimate the dissolved Li export flux in moderate erosion regions, and used sediment flux from the sub-marine fans and marginal seas in south and SE Asia to estimate that in high erosion regions (Fig. 1). Based our δ^7Li records, we assumed that the Li isotope composition in high erosion regions (similar to the Nepal Himalaya) remained constant since 15 Ma, while in moderate erosion regions (similar to the Qaidam Basin), it gradually decreased with global climate (here, we set δ^7Li to correlate with benthic δ^{18}O). Therefore, we can derive a comprehensive Li isotope composition for the entire plateau output by weighting the regional isotope values according to their respective Li flux contributions. Our model results suggest that, regardless of increasing or constant riverine Li flux from the Tibetan Plateau over the past 15 Myr, the estimated Li weathering flux from the Tibetan Plateau can only cause the tendency of seawater δ^7Li to be relatively flat (Fig. 5). These calculations demonstrate that riverine input from the Tibetan Plateau, driven by rapid erosion and global cooling, is unlikely to account for the observed rise in seawater δ^7Li at least over the past 15 Myr.

Collectively, the results of the modelling that takes into account our records suggest that Neogene seawater δ^7Li records are unlikely to reflect enhanced silicate weathering driven by mountain uplift, due to the mixing of other source and sink fluxes. Our results do not imply that weathering of the Tibetan Plateau is incapable of influencing Earth's carbon cycle. Rather, they

indicate that the Li flux supplied by silicate weathering of tectonically active regions alone may be insufficient to measurably alter the marine Li cycle. This implies that changes in the alteration of the oceanic crust, uptake of Li into marine sediments and continental weathering outside active mountain belts have to be taken into account to explain the δ^7Li seawater record in the last 15 Ma. Indeed, the oceanic Li budget is strongly modulated by seafloor hydrothermal inputs and submarine reverse weathering (*18-19*), which may outweigh the continental contribution. In addition, Li export from continents can be substantially influenced by non-weathering processes, including hydrological variability and aridity (*16-17*), as well as inputs from geothermal waters (*83*). By examining the Cenozoic uplift of the Tibetan Plateau as a natural experiment, our study demonstrates the limitations of using marine Li isotopes alone to infer long-term weathering-driven CO_2 consumption. We therefore emphasize the importance of reconstructing silicate weathering histories in major orogenic regions, such as the Tibetan Plateau and the Andes, which provide the most direct geological archives of tectonically driven carbon cycling. Our Li isotope records and modelled results underscore that the Li cycle associated with hydrothermal systems and submarine reverse weathering is needed to explain the observed Neogene seawater δ^7Li records, and that it is important to better constrain these complex marine source-sink processes."

Overall, the data set presented here is crucial for our understanding of the climate evolution over the end of the Cenozoic. However, I think that the conclusions are not supported by their model analysis. I would send this paper to a specialized journal.
I hope that my comments will help.

Reply: We sincerely thank the reviewer for recognizing the importance of our dataset for understanding late Cenozoic climate evolution. In the revised manuscript, we have substantially strengthened the evidential basis of our conclusions. Specifically, we have incorporated new Li isotope records from the southern Tibetan Plateau with high erosion flux —representing the first systematic presentation of continuous paleo-lake Li isotope records from both the northern and southern Tibetan Plateau (see Figs. 1, 2, 5).

In addition, we have rigorously constrained and tested the Li flux evolution using four likely approaches in our revised model framework, which also includes the C-Li coupling approach as mentioned by the reviewer.

We hope that these major additions and methodological improvements will allow the reviewer to reassess the robustness and broader significance of our findings.

Response to Reviewer #2:

Reviewer #2 (Remarks to the Author):

The manuscript entitled "Limited Contribution of Continental Weathering to Late Cenozoic Seawater Li Isotope Evolution" presents a systematic investigation of Li isotopic compositions in drill cores from the Qaidam Basin, northeastern Tibetan Plateau. Based on the water-soluble and carbonate phases, the authors reconstructed paleowater δ7Li values and observed a decrease of ~10‰ over the past ~7 Myr. This trend contrasts with the slight increase in seawater δ7Li values during the same period. Using a box model, the authors conclude that the evolution of seawater Li isotopes during the late Cenozoic cannot be primarily attributed to riverine input from the Tibetan Plateau. The findings of this study may contribute to improving our understanding of Cenozoic weathering. The manuscript is well-written, but I believe the authors could further refine their interpretations. I would recommend a decision of moderate revision.

Reply: We greatly appreciate the reviewer's recognition of our study. We have carefully considered the suggestion and modified the relevant parts in the manuscript.

My major concerns lie in sample representativeness and data interpretations:

1, The authors appear to have overestimated the riverine Li flux from the Tibetan Plateau (Line 26). The Pearl River and Hong He do not originate from the Tibetan Plateau. The dissolved Li concentration in the Mekong River was cited from its upper stream (referred to as the Lancang River; Li et al., 2023). However, the water discharge of the Lancang River is only about 64 km3/yr, compared to 550 km3/yr for the entire Mekong River. A more conservative calculation would be preferable. Furthermore, the drill cores are located in a hydrologically closed lake (Line 75), meaning the lake water does not flow into the ocean. How can these samples be used to investigate the influence of Tibetan Plateau weathering on seawater Li isotopic evolution (Lines 309-311)? Is it possible that the Li isotopic variation simply reflects lake evolution (e.g., changes in the sedimentary environment) rather than continental weathering?

Reply: We thank the reviewer for these constructive comments. Following the reviewer's suggestions, we have revised our calculation of the riverine Li flux from the Tibetan Plateau. Specifically, we updated the discharge of the Lancang River and calculated the two outcomes of removing the Pearl River and Hong He and including the Pearl River and Hong He. The revised estimate yields a total Tibetan Plateau riverine Li flux of 1.47×10^9 mol/yr and 1.53×10^9 mol/yr. It indicates that the entire plateau contributes approximately 18% of the global riverine Li flux. In this study, we used the more conservative date (1.47×10^9 mol/yr) as endmember for model calculation.

Regarding the concern about using drill-core records from the hydrologically closed Qaidam Basin to infer the weathering influence of the Tibetan Plateau on seawater δ^7Li evolution, we agree that such lakes do not directly contribute solutes to the ocean. However, a large closed-basin lakes do reliably archive the Li-isotope evolution of the rivers feeding them, and thus preserve information on continental weathering processes occurring in the uplifting mountain ranges. Given this

advantage, we believe that this type of archive is representative of mountain weathering. To further strengthen the representativeness and robustness of our interpretation, we have incorporated newly obtained Neogene Li-isotope records from the monsoonal southern Tibetan Plateau into the revised manuscript. We conducted systematic δ^7Li analyses of both carbonate and clay fractions from well-dated Siwalik Group sediments in the Nepal Himalaya (15–5 Ma). These new records reveal persistently low paleowater δ^7Li values and weak isotope fractionation in the southern Himalaya, which we attribute to intense erosion that shortens water–rock interaction times and limits clay formation—producing more congruent weathering. By integrating these new southern monsoonal data with our previous northern Tibetan Plateau record, the revised manuscript now presents a more spatially comprehensive and representative reconstruction of weathering evolution across the Plateau. The contrasting behaviors between the northern arid and southern monsoonal regions provide unexpected new insights into how climatic and geomorphic regimes shape Li-isotope signatures and silicate weathering processes.

In addition, we carefully evaluated this in our revision: the δ^7Li values remained unaffected by evaporation during the transition from semi-deep to shallow lake conditions, and only in the late-stage dry saline playa (after ~0.5 Ma) did lake processes begin to modify the isotopic composition. We therefore conclude that the long-term Qaidam Basin δ^7Li record primarily reflects continental weathering rather than lacustrine effects.

We further strengthened our discussion in Lines 69-82 as "To constrain the contribution of riverine Li input from the Tibetan Plateau to the ocean over tectonic timescales, we established Neogene paleowater δ^7Li records from both the southern and northern parts of the Tibetan Plateau, given the distinct climate and denudation regimes within the plateau. These records are based on a 3500-m-thick fluvial sequence (15–5 Ma) from the Siwalik foreland basin in the foothills of the Nepal Himalaya and a 1700-m-deep drill core (7.3–0.1 Ma) from the Qaidam Basin on the northern Tibetan Plateau (Figs. S1 and S2). The Siwalik Basin and Qaidam Basin lie within the southern Asian monsoon humid region and the mid-latitude westerlies arid region, respectively, and both are bordered by mountain ranges that experienced intensified tectonic uplift and climatic cooling during the Neogene (23-25). Sediments in these basins are therefore ideal archives that typify change in silicate weathering associated with the Neogene uplift of the Tibetan Plateau. We provide the first systematic reconstruction of Li isotopic variations across the northern and southern plateaus during the Neogene. It establishes a critical basis for evaluating how erosion and climate change jointly influence continental weathering and Li isotope behaviour, and provides key constraints on the contribution of continental weathering to the rise of seawater δ^7Li."

And in Lines 209-223 as "The sedimentary facies of SG-1 and SG-1b cores support this interpretation. No substantial evaporite deposits are observed in either the Karnali section or the SG-1b core, suggesting that crystallization-driven Li isotope fractionation played a negligible role during earlier periods. In contrast, thick salt layers occur in the uppermost part of the SG-1 core (Fig. S2), marking the onset of extensive evaporite formation.… Together, these lines of evidence suggest that crystallization-driven fractionation associated primarily with carbonate precipitation

could have exerted a first-order control on paleowater δ^7Li during the terminal stage of lake desiccation since ~0.5 Ma, while having little influence during earlier phases of lake evolution"

And in Lines 302-305 as "We first quantified the modern riverine Li fluxes across the plateau through statistical analyses (Table 1). The results indicate that major rivers originating from the Tibetan Plateau collectively export a Li flux of approximately ~1.5×10^9 mol/yr to the ocean (accounting for ~18% of global river input) (Table 1)."

2, Evidences for excluding contamination and diagenetic effects are insufficient. For instance, in lines 83 and 114, the term "non-carbonate and detrital contamination" was used. The authors only examined the impacts of detrital contamination. In my opinion, the potential release of Li from Fe-Mn oxyhydroxides should also be considered; otherwise, the terms "non-carbonate" and "detrital" are repeated. Please also evaluate the potential influence of gypsum on the Li isotopic composition of the carbonate phases, considering that acetic acid (HAc) used in extraction might dissolve gypsum and release its Li. Regarding the diagenetic effects, in lines 94–96, a higher content of aragonite relative to calcite and dolomite is not sufficient evidence to exclude diagenetic effects. How can the higher-than-normal Li/(Mg+Ca) ratios (52–693 μmol/mol) in the carbonate fraction be explained? In my view, the potential influence of diagenesis on both the water-soluble phase and carbonate was not thoroughly investigated. Changes in the sedimentary environment may affect pore water evolution, which could indirectly alter the Li isotopic compositions of halite and carbonate.

Reply: We sincerely thank the reviewer for raising these important points regarding contamination and diagenetic effects. In the revised manuscript, we have strengthened the evidence and expanded our discussion to address these concerns.

First, we now explicitly evaluate the potential release of Li from Fe-Mn oxyhydroxides by Mn/Ca ratios. Together with low Al/Ca, K/Ca, and Mn/Ca ratios in both HOAc and water leachates, and the absence of correlations between these ratios and δ^7Li, the results indicate minimal contribution from non-carbonate fractions, including Fe–Mn oxyhydroxides. We additionally assess the potential impact of gypsum dissolution during acetic-acid leaching. The XRD analysis shows that gypsum contents in the sediment are with average ~5%. We also supplied the anions contents (Cl^-, SO_4^{2-}) of the water leachate of sediments. The SO_4^{2-} contents of water leachates (~6328 ug/g) indicate that ~1g sediment contains a maximum of ~0.04g gypsum ($CaSO_4 \cdot 2H_2O$) assuming that sulfate is mainly composed of gypsum as shown by XRD analyses. Depending on the amount of water during water leaching and rinsing processes (40ml*4=120ml) and the solubility of the gypsum (~0.2-0.25g of gypsum is dissolved in 100ml water at 25°C), we can obtain that the 120ml H_2O could dissolve ~0.32g at least. Hence, the dissolved amount is much higher than the amount of gypsum contained in the sample. The influence of gypsum on ammonium acetate leaching is very weak. Our pretreatment using water and NH_4Ac effectively removes Li from exchangeable and gypsum, and the data show that gypsum-derived Li has negligible influence on carbonate δ^7Li.

Second, we enhanced the discussion of diagenetic effects on both carbonate and water-soluble phases. Carbonate δ^7Li shows no significant relationship with Mn/Sr, and the Li/(Ca+Mg) ratios—although relatively high in some Qaidam Basin samples—do not correlate with δ^7Li. These higher ratios likely reflect saline lake conditions rather than diagenesis. This is because this higher Li content than fluvial carbonate is also found in lacustrine carbonates in other regions (Chen et al., 2023 GRL). In addition, soluble salt and carbonate phases display distinct absolute δ^7Li values but yield consistent long-term reconstructed signals, indicating that porewater evolution did not alter the Li isotopic system. The presence of abundant aragonite further supports weak early diagenesis.

Collectively, these new analyses and expanded discussions demonstrate that the Li isotopic compositions of the carbonate, soluble salt, and exchangeable phases faithfully preserve primary paleoenvironmental signals, with minimal contamination or diagenetic modification.

We further modified the discussion on contamination and diagenetic effects in Lines 93-130 as "Diagenetic processes can modify the original Li isotopic signature of carbonate and clay minerals in sediments (26-28). However, multiple lines of evidence suggest that diagenetic effects are minimal in this study (Figs. S4-S5). There is no significant correlation between the carbonate δ^7Li and Mn/Sr from both basins, suggesting weak meteoric diagenetic influence (*29*) (Fig. S4). The Li/(Ca+Mg) ratios of the carbonate fraction (Fig. S4) are higher than those observed in meteorically altered low-Mg calcite (*26*). The absence of correlation between Li/(Ca+Mg) and δ^7Li further supports limited diagenetic influence. The higher Li/(Ca+Mg) ratios of the Qaidam Basin samples may result from evaporative concentration of salty lake water (the paleo-environment of the Qaidam Basin), a common feature in other lacustrine sediments (*30*). Moreover, the water-soluble and carbonate phases show distinct δ^7Li values but yield similar reconstructed evolution (Fig. S3), implying that variations in mineralogy and porewater chemistry have not affected the Li isotope records in the Qaidam Basin. The aragonite-dominated carbonate mineralogy also indicates weak early diagenesis (Data S1). Regarding burial diagenesis, neither the δ^7Li values of carbonate nor those of the <2 μm clay-sized fraction from the Qaidam Basin (SG-1 and SG-1b cores) or the Nepal Himalaya (Karnali section) show any decrease trend with depth (Fig. S3). This indicates that diagenetic processes exerted only minor control over the Li isotopic composition. The clay minerals, which are exceptionally Li-rich, did not experience significant compositional alteration due to burial diagenesis. Likewise, no notable increase in Li content or evidence of substantial modification was observed in the carbonate phases. Moreover, in the SG-1 and SG-1b cores, the proportion of smectite layers in illite-smectite mixed layers (S%) increases with depth—a trend that contrasts with typical diagenetic progression patterns (Fig. S5). Collectively, these observations provide compelling evidence that the analysed samples have retained their primary geochemical signatures with minimal diagenetic modification.

Paleowater δ^7Li signals reconstructed from carbonates or soluble salt in fluvio-lacustrine environments are readily affected by non-carbonate inputs and detrital contamination during sample digestion (*33-34*). To minimize this, first we used ultrapure water (18.2 MΩ*cm) to extract the water-soluble salts. Then, we used 1 M ammonium acetate (NH4Ac) to leach exchangeable lithium

from clays and to dissolve remaining gypsum, if any, as a pre-cleaning step prior to carbonate digestion with 1 M acetic acid (HOAc) (*34*). In all the 1 M acetic acid (HOAc) and water leachates of the bulk samples, the low Al/Ca, K/Ca, Si/Ca, Rb/Ca, and Mn/Ca ratios and lack of correlation between δ^7Li and those ratios suggest minimal influence from non-carbonate fractions (e.g., silicate fraction and Fe-Mn oxyhydroxides) in the leaching process (Fig. S4). We minimized the detrital influence on δ^7Li of soluble and carbonate fractions by selecting samples with high carbonate proportions (>3%, with an average of ~15%) through rigorous microscopic analysis of the samples. Meanwhile, carbonate δ^{13}C and δ^{18}O values of the Qaidam core sediments and bedrock samples from the surrounding source regions reveal little contamination from detrital carbonates (Fig. S5). The δ^7Li values of the soluble salt, carbonate, and exchangeable fractions of the sediments also show similar long-term trends in the Qaidam Basin (Fig. S3), suggesting that the Li isotopic compositions of these phases likely recorded the same underlying processes. Microscopic observation revealed that in our measured samples with high carbonate content in the Nepal Himalaya sediments, authigenic carbonate was predominant (Fig. S6), suggesting that the detrital influence is limited."

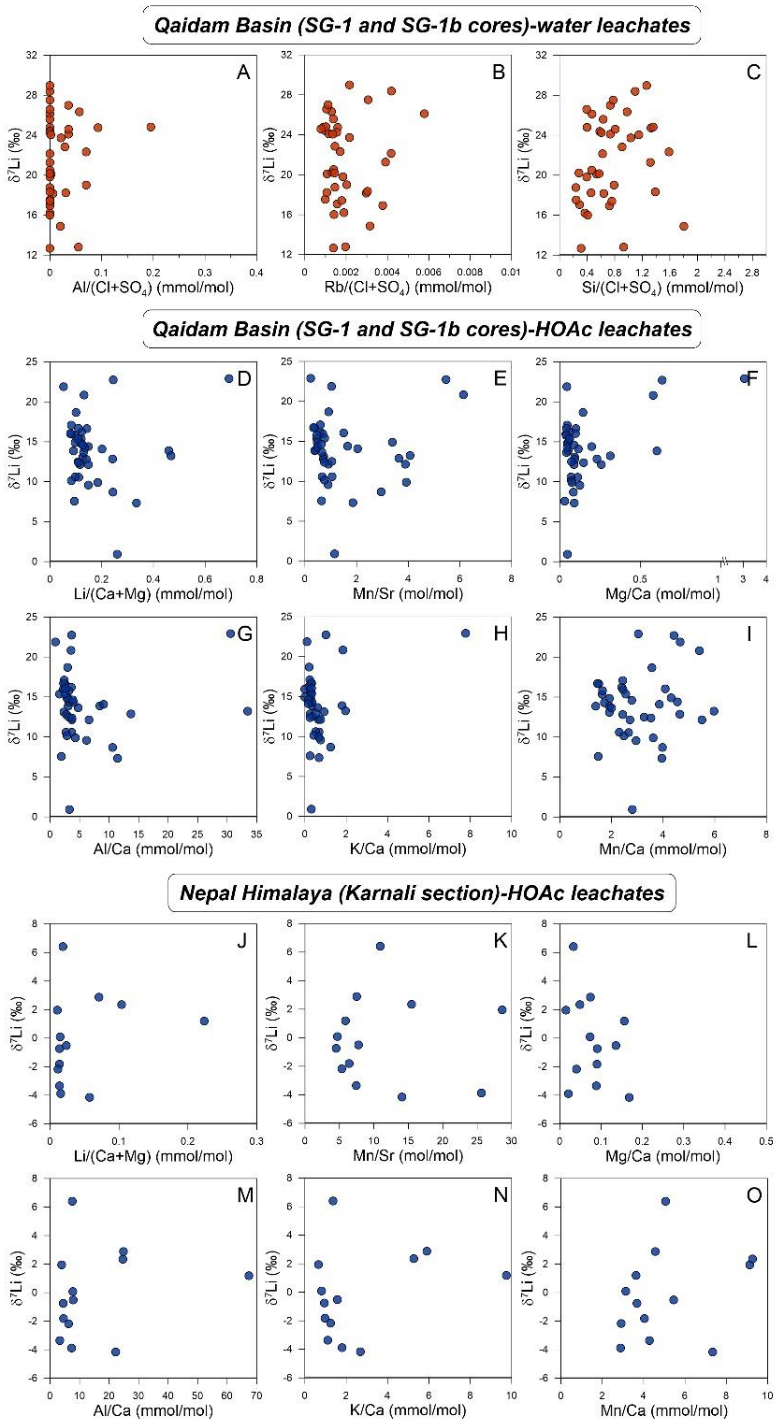

3, Weathering intensity and weathering rate are distinct concepts, and they are influenced differently by climate and tectonic factors. I believe the authors have sometimes misused these terms. For instance, in tectonically active regions, weathering rate is often sensitive to climate changes (i.e., weathering-limited regimes). Generally, Li isotopic composition reflects weathering intensity. In this context, I disagree with the explanation in Lines 261-264, which suggests a negligible influence of tectonic activities. Additionally, the sedimentation rates of SG-1 and SG-1b may not accurately represent the erosion rate of the entire Tibetan Plateau, as

they are from different sedimentary environments (depositional center vs. Jianshan Anticline). It would be preferable to include regional erosion rate data. Similarly, was the cited temperature drop of 5–8 °C (Line 239) derived from terrestrial or deep-sea records? I would prefer to see regional temperature reconstructions. Moreover, the global mean riverine Li isotopic composition has been updated recently. Please consult the reference by Läuchli et al. (2025). The value of δ7Liriv_other in Table 2 is 25.6‰, which may be unreasonably high and could affect your model. Fourth, how was the W/D (weathering flux/denudation flux) ratio over the past 8 Ma derived (Figure 3)? The riverine Li flux from the Tibetan Plateau is important for explaining the slight increase in seawater δ7Li. Perhaps an increase in this flux offset the decrease in riverine δ7Li. Fifth, how was Friv_other set during the modelling? Global weathering flux is proposed to have remained constant over the Neogene (Caves Rugenstein et al., 2019). If you did not adopt this assumption, please provide justifications.

Reply: We thank the reviewer for the constructive comments. When integrating both northern and southern Tibetan Plateau records, we indeed find that the intensity of tectonic activity and the magnitude of erosion strongly influence the Li isotopic signals and the inferred weathering intensity. Following the reviewer's suggestion, we have incorporated regional erosion rate data for comparison in Figure 2.

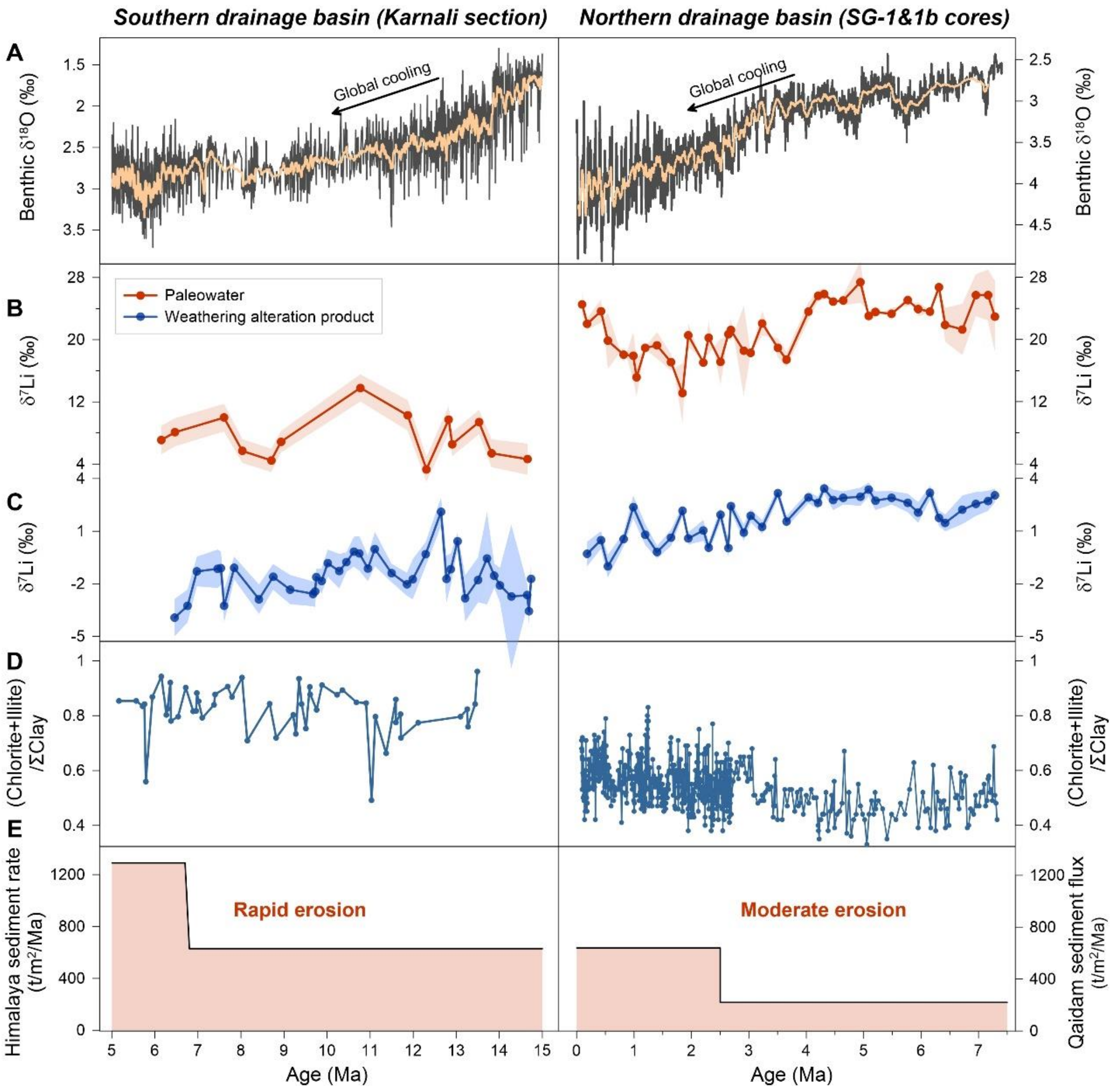

Fig. 2. Multiphase Li isotope records of the southern and northern Tibetan Plateau compared with other climate, weathering and denudation records. (A) Global marine benthic foraminiferal $\delta^{18}O$ record (*52*), (B) paleowater $\delta^{7}Li$ records based on carbonate and soluble salt fraction from the Nepal Himalaya and Qaidam Basin (pink shading represents the error of the reconstructed values), (C) weathering alteration product $\delta^{7}Li$ record corrected from clay fraction from the Nepal Himalaya and Qaidam Basin, (D) (chlorite+illite)/Σclay ratio of SG-1 and SG-1b cores as well as Bengal Fan sediment (94-96), (E) sediment accumulation rates of Himalaya region and Qaidam Basin (modified from refs. *79-80*).

The 5–8 °C temperature decrease cited in the original manuscript was derived from marine records. We have now added regional terrestrial temperature reconstructions in Lines 224-227, which similarly indicate a cooling of approximately 6.6 °C (Liang et al., 2025).

Y. Liang, H. Wang, D. B. Kemp, Z. R. Liu, W. Liu, H. Liu, C. Quan, X. Liu, Z. Liu, M.-F. Zhou, Terrestrial temperature history reveals Late Miocene rapid uplift of the northern Tibetan Plateau. Sci. Adv. 11 (2025).

The $\delta^7Li_{riv\_other}$ value in Table 2 represents the global mean riverine δ^7Li, calculated by excluding contributions from the Tibetan Plateau region. The new result is 23.8‰, which we think is more reasonable. The W/D ratios during 15–0 Ma are based on silicate weathering fluxes (W) derived from CO_2 consumption fluxes and reconstructed erosion fluxes (D). The CO_2 consumption fluxes were estimated based on the relative loss of elements such as Ca, Mg, Na, and K. Therefore, we reconstructed silicate weathering fluxes using the history of weathering-driven carbon consumption in the southern plateau as reconstructed by Clift et al. (2024).

Regarding Li flux evolution, we recognize its crucial role in the model. While an increase in Li flux could potentially offset decreases in riverine δ^7Li, we have now applied four complementary approaches to more rigorously constrain Li flux changes and examine the impact of flux increases: 1) Erosion-based scaling: Past Li export varied proportionally with Neogene plateau erosion flux changes (Métivier et al., 1999; Fig. 5B). 2) Silicate weathering reconstruction: Carbon consumption fluxes and major cation and Li loss were coupled to constrain Li flux from reconstructed silicate weathering rates (Clift et al., 2024; Fig. 5C). 3)W/D–Li flux relationship: Empirically derived relationships among Li flux, δ^7Li, and the weathering-to-denudation ratio from modern rivers were used to infer past Li flux from isotopic signatures (Pogge von Strandmann et al., 2021; Fig. 5D). 4) Constant weathering scenario: Li fluxes constrained by Be isotope records were held constant (Willenbring & von Blanckenburg, 2010; Fig. 5E). Our results show that under all scenarios, Li flux variations cannot drive the observed seawater δ^7Li increase, reinforcing our original conclusions.

In the model, F_{riv_other} was set as constant, consistent with the assumption that the global weathering flux remained approximately steady during the Neogene (Caves Rugenstein et al., 2019).

We further modified the discussion in Lines 300-317 as "It is essential to constrain both the Li flux and isotopic composition input from the Tibetan Plateau. Reconstructing past variations in riverine Li flux from the Tibetan Plateau remains highly challenging due to the absence of direct reconstructions of ancient river discharge and Li concentrations. We first quantified the modern riverine Li fluxes across the plateau through statistical analyses (Table 1). The results indicate that major rivers originating from the Tibetan Plateau collectively export a Li flux of approximately ~1.5×10^9 mol/yr to the ocean (accounting for ~18% of global river input) (Table 1). We then employed four scenarios to constrain the Li flux evolution of the Tibetan Plateau over the past 15 Myr (see Methods). First, the past Li flux varied proportionally with variations in the Neogene erosion flux of the northern and southern plateau (Fig. 1B) (*63*). Second, the atmospheric CO_2 consumption flux by silicate weathering of the Tibetan Plateau (*79*) constrain the dissolved Li flux based on the C-Li coupling assumption that Li is congruently released, similar to the way base cations (Na, K, Ca and Mg) are depleted during silicate weathering (13) (Fig. 5C). Third, the Li flux through time is constrained by the empirically-derived relationship with δ^7Li, the weathering-to-denudation (W/D) ratio from modern rivers (*64*), and our measured δ^7Li signatures (Fig. 5D). Fourth, temporal changes in Li flux is linked to the constant Tibetan Plateau erosion flux based on based on constant exhumation and erosion rates (*47, 58, 65*) (Fig. 5E). Subsequently, we tested the effect of these four different scenarios separately in our Li mass balance model and these four scenarios

essentially encompass the current understanding of the weathering-induced lithium flux on the Tibetan Plateau during the Neogene period."

Other comments:

Lines 90–91, references 29–30 only discuss the influence of diagenetic processes on carbonate minerals. Please add appropriate references that address silicate minerals.

Reply: Thank you for pointing it out. We have added the references about silicate minerals in Lines 93-94 as "Diagenetic processes can modify the original Li isotopic signature of carbonate and clay minerals in sediments (*26-28*)."

C. Yang, N. Vigier, S. Yang, M. Revel, L. Bi, Clay Li and Nd isotopes response to hydroclimate changes in the Changjiang (Yangtze) basin over the past 14,000 years. Earth and Planetary Science Letters 561, 116793 (2021).

Line 132, the scale in Figure S4 is too large to discern details of the Mg/Ca variations.

Reply: The scale of Figure S4 has been adjusted to better display the detailed variations in Mg/Ca ratios.

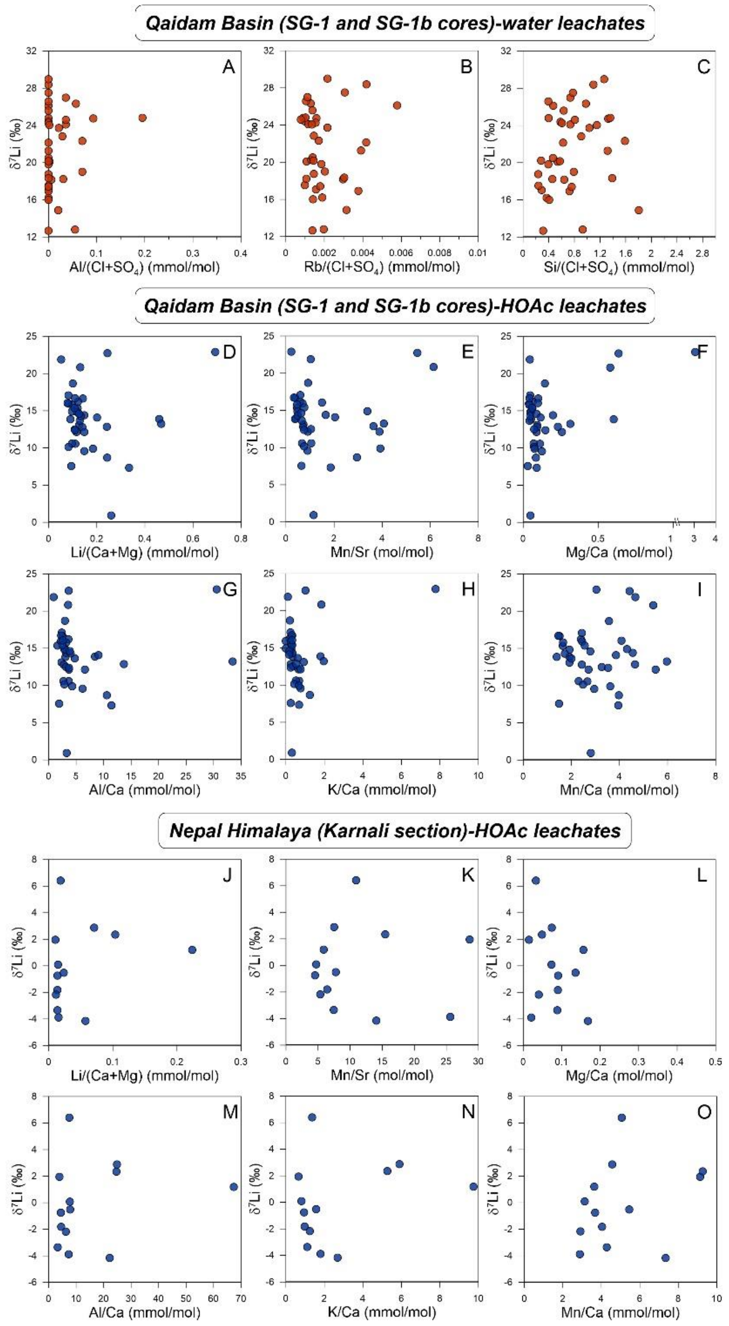

Line 134, please carefully verify the fractionation factor of "-8.5±2‰ for inorganic calcite". This value is not commonly used in the literature.

Reply: Thank you for raising this point. The fractionation factor of –8.5±2‰ is based on Day et al. (2021), who experimentally determined this value for terrestrial carbonates. We believe this value is more appropriate for our lacustrine carbonate record than marine calcite fractionation factors. Additionally, this fractionation factor has also been applied by Chen et al. (2023).

Line 140, during halite precipitation, light 6Li is preferentially incorporated into the crystal structure. However, the majority of Li in halite is stored in non-fractionated fluid inclusions,

which is the rationale for effectively setting the fractionation factor to 0‰. The authors should clarify this point to avoid confusion with the general statement in Line 205 that "light Li isotopes (6Li) preferentially partition into precipitating carbonate and evaporite minerals."

Reply: We thank the reviewer's advice. We added the explanation to avoid confusion in Lines 143-145 as "we adopt an average fractionation factor between soluble salt and water, defined as $\Delta^7Li_{salt-water}$ ($\delta^7Li_{salt}-\delta^7Li_{water}$), of ~0±1‰ (*40-42*). This low fractionation factor is likely related to the majority of Li in halite being stored in non-fractionated fluid inclusions."

Line 152, the 87Sr/86Sr ratios of the carbonate phase were not measured. Could the authors explain why? This data seems important for a comprehensive interpretation. Please seperatively display the 87Sr/86Sr ratios somewhere as what you did in Figure S7.

Reply: Thank you for the valuable comment. Previous studies on lacustrine sediments have shown strong 1:1 correlation between $^{87}Sr/^{86}Sr$ ratios in carbonates and those in evaporites (Liu et al., 2023, 2024). Therefore, analyzing a single phase is generally sufficient for reconstructing the paleo-lake $^{87}Sr/^{86}Sr$ composition. Since the primary focus of this study is Li isotopes, we did not perform additional Sr isotope measurements on the carbonate fraction. After incorporating new Li isotope data from the southern Tibetan Plateau, we have removed the Sr isotope results from the revised manuscript and now concentrate on interpreting the two Li isotope profiles from the northern and southern Tibetan Plateau.

Line 156, please consider adding relevant references that discuss Li isotopes in clay-sized fractions to support the methodology or interpretation here.

Reply: Thank you for the suggestion. We have added relevant references that use Li isotopes in clay-sized fractions to trace weathering processes, including Yang et al. (2021) and Chen et al. (2023) in Lines 157-158 as "Weathering alteration products (i.e., clay minerals) provide an independent perspective on catchment weathering processes (*28-30*)."

C. Yang, N. Vigier, S. Yang, M. Revel, L. Bi, Clay Li and Nd isotopes response to hydroclimate changes in the Changjiang (Yangtze) basin over the past 14,000 years. Earth Planet. Sci. Lett. 561, 116793 (2021).

Z. Chen, Z. Ding, S. Yang, J. Sun, M. Zhu, Y. Xiao, F. Tong, Y. Liang, Strong Coupling Between Carbon Cycle, Climate, and Weathering During the Paleocene-Eocene Thermal Maximum. Geophys. Res. Lett. 50, e2023GL102897 (2023).

Line 171, the claim that "the impact of recycled sedimentary materials is minor" is unclear. In Figure S10A, the data points for clay-sized samples predominantly fall within the range of the shale endmember, which seems to suggest a significant influence. Could this be explained further?

Reply: Thank you for your insightful comment. After adding new Li isotope data from the southern Plateau and redrawing the figure, we now observe that samples from both profiles do not plot near

the shale endmember. Because our samples are clay-sized fractions, enrichment in weathering products is expected. Shales represent lithified ancient weathering residues, which can lead to partial overlap in isotopic composition.

We have revised the text to clarify this reasoning in Lines 161-166 as "Second, we use the relationship between the Weathering Index of Parker (WIP) and the Chemical Index of Alteration (CIA), which serves as a useful proxy for tracing shale recycling (*45*), to evaluate the potential contribution of shale and the lithological characteristics of the source area. Shales represent lithified ancient weathering residues, which can lead to partial overlap in isotopic composition. The WIP-CIA relationship suggests that the impact of recycled sedimentary materials is minor (Fig. S8)."

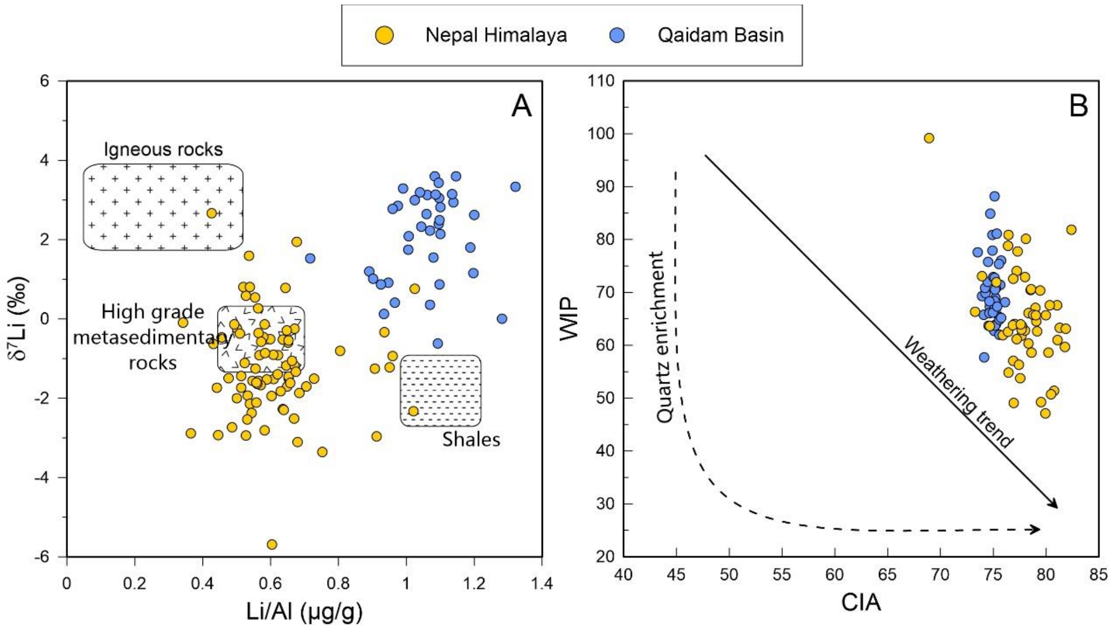

Line 192, please provide a brief explanation of why these specified proxies are effective for distinguishing between different sources. Is the distinction primarily based on grain size, geochemical composition, or other factors?

Reply: Thank you for your advice. We added the explanation in Lines 183-184 as "These elements are largely unaffected by chemical weathering and thus preserve the geochemical signatures of the source rocks (46)."

Line 203, this section requires clarification. First, I agree with the statement that "light Li isotopes (6Li) preferentially partition into precipitating carbonate and evaporite minerals" (Line 205). However, carbonate and evaporite are not Li-rich minerals. The resulting flux is likely negligible compared to the total Li reservoir in the lake, making an observable increase in the water's δ7Li value improbable. Therefore, it seems unrealistic that 90% of Li could be incorporated into authigenic minerals if these are carbonate and evaporite. Second, the choice of fractionation factors (0.999, 0.997, 0.995, 0.992) needs justification. Which specific minerals

do these factors represent? Third, the salt layer is noted above ~414 m (~1.2 Ma), yet 0.5 Ma is used as a boundary. What is the reason for this discrepancy? Could the anomalous values be related to gypsum? Generally, δ7Li values of gypsum are higher than contemporaneous halite.

Reply: We thank the reviewer for these insightful comments. We agree that during most stages of lake evolution, the Li flux incorporated into carbonate and evaporite minerals is very small because these minerals are not Li-rich. However, the Qaidam paleolake ultimately evolved into a desiccated saline pan. In this terminal stage, essentially all dissolved Li must be incorporated into newly formed minerals—carbonate, evaporite, and any potential authigenic clays. This interpretation is supported by the marked increase in Li concentrations observed in these minerals during the final stage. Because neither our XRD analyses nor petrographic observations reveal the appearance or increase of authigenic clay minerals, we infer that carbonates and evaporites are the primary Li hosts during this interval. This is the basis for evaluating the scenario in which ~90% of Li becomes incorporated into authigenic phases. Considering that the fractionation factor for halite is nearly 0‰, this may highlight the predominant role of carbonate and gypsum precipitation. In terms of Li content, the Li concentration in carbonates from the Qaidam borehole is one order of magnitude higher than that in water-soluble salts, further supporting this point.

We acknowledge the reviewer's concerns and hence modified the discussion in Lines 195-223 as "Fractionation associated with the precipitation of authigenic minerals has the potential to modify the isotopic evolution of Li in lake water, particularly under conditions of extensive mineral crystallization (*51*). During mineral precipitation, light Li isotopes (^{6}Li) preferentially partition into solid phases such as carbonates and evaporitic minerals, leading to an enrichment of ^{7}Li in the residual water. This effect can be quantified using mineral–fluid fractionation factors ($\Delta^7Li_{carb-fluid}$ and $\Delta^7Li_{salt-fluid}$). To assess the maximum possible influence of crystallization-driven fractionation on lake-water δ^7Li, we applied a Rayleigh fractionation model to simulate δ^7Li evolution under a range of fractionation factors. Model results indicate that if up to ~90% of dissolved Li is removed from the lake water and incorporated into authigenic minerals, representing the end-member scenario characteristic of a terminal, desiccating saline pan, the δ^7Li of residual water would increase by 2.3‰ (α = 0.999), 6.9‰ (α = 0.997), 11.5‰ (α = 0.995), and 18.4‰ (α = 0.992, Fig. S11). Such extensive Li removal is unlikely during most stages of lake evolution because carbonates and evaporites are generally not Li-rich phases. However, in the final stage of paleolake desiccation, essentially all dissolved Li must ultimately be incorporated into newly precipitated minerals, making this scenario plausible for the late Quaternary (the last 0.5 Ma) evolution of the Qaidam Basin. The sedimentary facies of SG-1 and SG-1b cores support this interpretation. No substantial evaporite deposits are observed in either the Karnali section or the SG-1b core, suggesting that crystallization-driven Li isotope fractionation played a negligible role during earlier periods. In contrast, thick salt layers occur in the uppermost part of the SG-1 core (Fig. S2), marking the onset of extensive evaporite formation. Correspondingly, Li concentrations in both water leachates and 1 M HOAc leachates increase sharply after ~0.5 Ma, indicating a substantial transfer of Li from lake water into newly precipitated authigenic minerals (Fig. S11). Most samples show no positive correlation

between δ^7Li and Li/Na (for soluble salts) or Li/Ca (for carbonates), except for the upper part of the SG-1 core younger than ~0.5 Ma, where such correlations emerge. Although the Li isotope fractionation factor of halite is close to 0‰, gypsum and carbonate maybe still as the dominant driver of isotopic fractionation during this interval. Li concentrations in carbonates from the Qaidam record are an order of magnitude higher than those in water-soluble salts, supporting the interpretation that both are major hosts. Together, these lines of evidence suggest that crystallization-driven fractionation associated primarily with carbonate precipitation could have exerted a first-order control on paleowater δ^7Li during the terminal stage of lake desiccation since ~0.5 Ma, while having little influence during earlier phases of lake evolution."

Regarding the choice of fractionation factors (0.999, 0.997, 0.995, 0.992), these values represent the upper and lower bounds of Li isotope fractionation observed in previous experimental studies. They do not correspond to a specific mineral species; rather, they reflect the range of effective fractionation documented for complex precipitation systems comparable to our study setting.

We selected 0.5 Ma as the boundary because this is when Li concentrations in both soluble salts and carbonates begin to increase. This timing is later than the first appearance of the salt layer (~1.2 Ma) because Li precipitation lags behind the initial formation of Na- and Cl-dominated evaporites. The anomalous δ^7Li values may indeed be related to the presence of gypsum, as gypsum typically exhibits higher δ^7Li than contemporaneous halite, and its influence becomes more pronounced during intervals of intensified evaporite formation.

Line 384: The description "the fine-grained lacustrine sediments… were selected" is ambiguous. Does this indicate that bulk sediments were manually chosen based on their fine-grained nature, or were they mechanically sieved to a specific grain size range? Please clarify the selection process.

Reply: Thank you for pointing this out. The original statement was intended to indicate that we avoided samples with clearly coarse sand-sized particles during selection in order to minimize potential hydrodynamic influences. We did not perform any physical sieving.

To avoid misunderstanding, we have revised the wording to: "The sediments from the Karnali section and SG-1 and SG-1 b cores were used for multiphase extraction and elemental, mineralogical, and isotopic analyses." in Lines 375-376.

Line 433: The reported δ7Li value for the BHVO-2 reference material appears lower than the commonly accepted consensus value in the literature. Please verify this measurement or provide a justification for the discrepancy.

Reply: Indeed, the values I obtained for the BHVO-2 standard sample are slightly lower than those reported in previous literature. We are not entirely sure what has caused this discrepancy. However,

our triplicate results, which include analyses from two different laboratories, are highly consistent. This should demonstrate the accuracy of our operational and analytical procedures.

Response to Reviewer #3:

Reviewer #3 (Remarks to the Author):

Yang et al present a new d7Li record from the late Neogene Qaidam Basin that shows declining d7Li at the same time at the marine record of d7Li increases slightly or is stable. They use scaling relationships to scale this record to the entire Himalayan-Tibetan Plateau system and argue that the declining d7Li indicates a decrease in weathering from tectonically active areas and that therefore these areas could not have driven Cenozoic cooling.

This was a fascinating paper to read, and I think the presentation of the paleo-d7Li record is outstanding. The authors do a very thorough job of ruling out diagenesis and contamination, and I'm impressed that this decline in d7Li is visible in two different archives—salt and carbonates. This makes this record especially robust.

Reply: We sincerely thank the reviewer for their thoughtful evaluation of our work and for the positive remarks on the quality and robustness of our reconstructed paleo-δ^7Li records. We greatly appreciate the recognition of our efforts in ruling out diagenetic alteration and in presenting consistent trends across both carbonate and salt archives.

With that said, I don't think these findings are as exciting as the authors claim. I'm very skeptical of the estimates that scale this result to the entire Tibetan Plateau-Himalayan system, and the decline in d7Li is the expected response for tectonically active areas that are driving cooling in response to a driver such as uplift. So, to me, there is no need to rethink anything…this is simply confirming the response on a local-scale. I don't know if this is a fixable problem, and I leave it to the editor to determine if this is major revisions or simply a rejection of the paper.

Reply: We fully understand the reviewer's concern regarding the extrapolation of a record from the arid northern Tibetan Plateau to the broader Tibetan Plateau. In response, we have substantially expanded our dataset in the revised manuscript by incorporating new Li-isotope records from the monsoonal southern Tibetan Plateau (Nepal Himalaya). These additions allow us, for the first time, to jointly analyze continuous paleo-water δ^7Li records from both northern and southern regions in terms of erosion and climate regimes, providing a more comprehensive spatial perspective on Tibetan Plateau weathering evolution. In addition, following the reviewer's suggestions, we have also implemented more refined constraints on Li flux evolution and tested multiple flux scenarios within our model framework. We hope that these new data and improved flux treatments address the reviewer's concerns and help clarify the broader implications of our findings.

The scaling of this finding to the entire Tibetan Plateau-Himalayan system is fairly suspect. The Qaidam Basin has been a closed basin for much of the Neogene (if not longer; Carroll et al. (2010)) and certainly for the entire length of the core presented. Almost by definition, a closed basin receives little moisture; in contrast, most of the weathering-action in the Himalaya-Tibetan Plateau (HTP) system takes place in areas that are today some of the wettest places on

Earth and are influenced by the two big Asian monsoonal systems. Thus, climatically, the Qaidam Basin is not at all representative of much of the Himalayan-Tibetan Plateau system. How then do these very different climate regimes influence the d7Li signature of the various rivers draining the HTP? There is very little evidence presented to suggest that the Qaidam Basin should even serve as a representative of the rest of the HTP. While the record that Yang et al. present is undoubtedly very interesting, it seems unlikely to be anything more than a local record in a fairly arid location.

Reply: We appreciate the reviewer's comment. Indeed, we fully agree that relying solely on a record from the arid northern Tibetan Plateau is insufficient to convincingly represent weathering conditions across the entire Tibetan Plateau. To address this limitation, we have incorporated newly obtained Neogene Li-isotope records from the monsoonal southern Tibetan Plateau into the revised manuscript.

Specifically, we conducted systematic δ^7Li analyses of both carbonate and clay fractions from well-dated Siwalik Group sediments in the Nepal Himalaya, and established continuous paleowater and clay δ^7Li records for 5–15 Ma based on a robust magnetostratigraphic framework. Our results show that, in contrast to the northern arid region, the southern monsoonal domain is characterized by persistently low paleowater δ^7Li values and weak isotope fractionation. We interpret this pattern as the consequence of intense and rapid erosion in the southern Himalaya, which shortens water–rock interaction times and limits clay formation, thereby producing more congruent weathering. Importantly, this low and weakly fractionated δ^7Li signal does not continue to decrease with further increases in erosion, suggesting the existence of a threshold erosion rate beyond which δ^7Li remains relatively invariant.

By integrating the new southern monsoonal records with our existing northern Tibetan Plateau record, the revised manuscript now provides a more representative and spatially comprehensive picture of weathering evolution across the Tibetan Plateau. Moreover, the contrasting behaviors revealed between the two regions offer new and unexpected insights into how distinct climatic and geomorphic regimes influence Li-isotope signatures and silicate weathering.

We further redraw a new figure 2 and elaborate this issue in Lines 69-82 and as "To constrain the contribution of riverine Li input from the Tibetan Plateau to the ocean over tectonic timescales, we established Neogene paleowater δ^7Li records from both the southern and northern parts of the Tibetan Plateau, given the distinct climate and denudation regimes within the plateau. These records are based on a 3500-m-thick fluvial sequence (15–5 Ma) from the Siwalik foreland basin in the foothills of the Nepal Himalaya and a 1700-m-deep drill core (7.3–0.1 Ma) from the Qaidam Basin on the northern Tibetan Plateau (Figs. S1 and S2). The Siwalik Basin and Qaidam Basin lie within the southern Asian monsoon humid region and the mid-latitude westerlies arid region, respectively, and both are bordered by mountain ranges that experienced intensified tectonic uplift and climatic cooling during the Neogene (*23-25*). Sediments in these basins are therefore ideal archives that typify change in silicate weathering associated with the Neogene uplift of the Tibetan Plateau. We provide the first systematic reconstruction of Li isotopic variations across the northern and southern

plateaus during the Neogene. It establishes a critical basis for evaluating how erosion and climate change jointly influence continental weathering and Li isotope behaviour, and provides key constraints on the contribution of continental weathering to the rise of seawater δ^7Li."

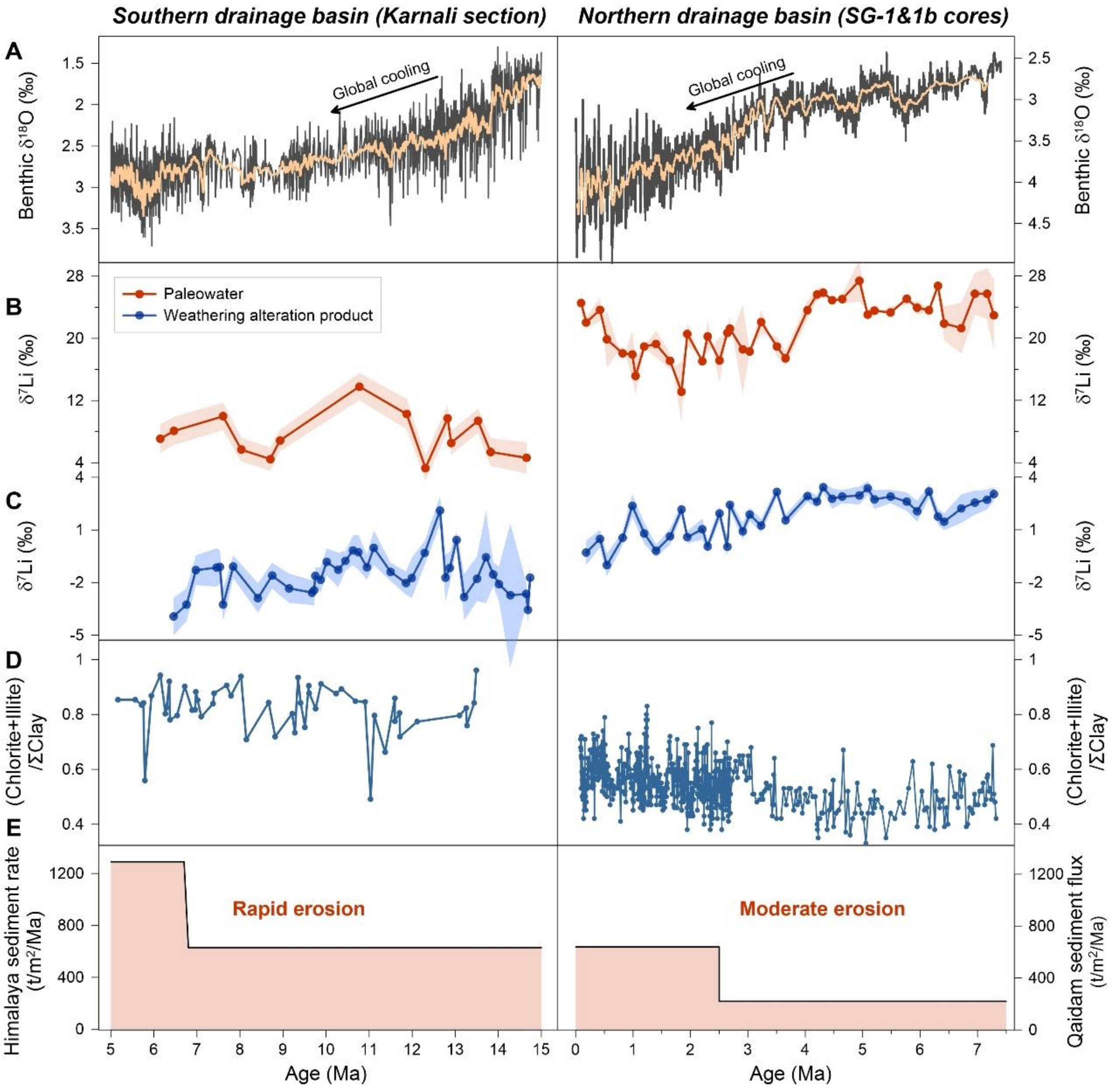

Fig. 2. Multiphase Li isotope records of the southern and northern Tibetan Plateau compared with other climate, weathering and denudation records. (A) Global marine benthic foraminiferal δ^{18}O record (52), (B) paleowater δ^7Li records based on carbonate and soluble salt fraction from the Nepal Himalaya and Qaidam Basin (pink shading represents the error of the reconstructed values), (C) weathering alteration product δ^7Li record corrected from clay fraction from the Nepal Himalaya and Qaidam Basin, (D) (chlorite+illite)/Σclay ratio of SG-1 and SG-1b cores as well as Bengal Fan sediment (94-96), (E) sediment accumulation rates of Himalaya region and Qaidam Basin (modified from refs. *79-80*).

In Lines 228-268 as "The δ^7Li of both paleowater and weathering alteration products is primarily controlled by the interplay between primary mineral dissolution and secondary mineral formation (*6, 54-56*). Given the established relationship between dissolved δ^7Li and silicate weathering intensity (W/D) in modern rivers (*6*), the observed trends in paleowater δ^7Li values can

be attributed to reduced weathering intensity under low to middle W/D ranges (Fig. 3; Figs. S12 and S13). In the northern Tibetan Plateau, global cooling and regional aridification have led to medium-to-low silicate weathering intensity. This interpretation is supported by sediment geochemistry and clay mineral assemblages from the region (Figs. S12 and S13). Regional weathering trends in the Qaidam Basin inferred from δ^7Li records, together with elemental and mineralogical proxies, exhibit a consistent temporal pattern with global (*52-53*) and regional (*25*) climate indicators during 7.3–0.5 Ma (Fig. S13 and S14). This synchronicity suggests that progressive regional aridification, ultimately driven by global cooling, was the dominant factor controlling Li isotope variations. The Qaidam Basin has always been outside the influence of the East Asian monsoon (*25*) and global cooling, which reduced moisture transport by mid-latitude westerlies, likely suppressed the formation of secondary minerals such as clays and consequently diminished Li isotope fractionation. The decreasing isotopic offset between lake water and weathering products further reflects diminished fractionation under decreasing catchment weathering intensity (Fig. S13).

In the Nepal Himalaya, although the regional climate is much humid under the influence of the South Asian monsoon, the Li isotope record shows little response to these climatic variations (Fig. 2). Instead, the persistently low and stable Li isotopic composition in both paleowater and secondary minerals indicates stable weathering conditions and low weathering intensity during the Neogene. This likely reflects the sustained high erosion rates in the Himalayan region since the Neogene. Intense tectonic uplift combined with heavy monsoon rainfall has maintained rapid surface erosion, resulting in extremely short water–rock interaction times (Fig. S15). This sustained rapid erosion regime during the Neogene is witnessed by exhumation records derived from ^{10}Be, Sr-Nd isotope and apatite fission-track lag-times during the Neogene (*57-59*), as well as much clay mineralogy with a much higher content of illite and chlorite than those in the Qaidam Basin (Fig. 2). Such conditions promote primary mineral dissolution but limit the formation of secondary clays, thereby producing long-term low δ^7Li values in river water. The reconstructed paleowater δ^7Li values are broadly consistent with those of modern Himalayan rivers (*15*), suggesting that this erosional regime has persisted for at least 15 million years. In addition, δ^7Li of bulk sediments shows a stable and nearly ~0 value, which further confirms this (*60*).

Although the Li isotope records from the southern and northern Tibetan Plateau show distinct long-term patterns, they can both be interpreted within the framework of silicate weathering intensity (W/D) and its associated processes of primary mineral dissolution and secondary mineral formation (Fig. 3). The apparent differences primarily reflect variations in regional erosion rates, which place their weathering conditions in different regimes. In the northern plateau, despite relatively active tectonics, the overall erosion flux remains moderate (Fig. 3). As a result, the effect of climatic cooling is amplified. In contrast, the Himalayas experience extremely high erosion fluxes. When denudation (D) is extremely high, climate fluctuations become insufficient to drive detectable changes in the W/D ratio. The obtained records from the southern and northern Tibetan Plateau capture variations in paleowater δ^7Li within the drainage areas of mountain ranges, reflecting the combined effect of Neogene tectonic uplift and global cooling."

Further, the authors present this record as a surprising result that should cause us to question the role of tectonically active ranges in the global weathering engine and in global cooling. I agree with the authors that a decrease in d7Li indicates a decline in weathering intensity, but this does not mean a decrease in the weathering flux as the authors often imply. There isn't any way that the authors can deduce this except through other methods not presented in the paper by, for example, using Be isotopes or estimating erosion rates to back-out the weathering flux implied by the weathering intensity. Instead, the decline in weathering intensity (and decrease in d7Li, given the position of these catchments relative to the Dellinger et al. (2015) curve (Figure 3)) is what is predicted from a global carbon cycle-climate driven by an increase in erosion. That is, the global weathering flux is determined by mass balance to equal the volcanic flux. As long as this isn't changing—and if erosion/uplift is increasing—then weathering intensity has to go done. How much it goes down (and if it goes down at all) in tectonically active areas is probably a function of lithology, rate of uplift increase, climate, etc., but this is poorly known. Regardless, all else being equal, most places on the planet will likely see a decrease in weathering intensity. So, while this is a very neat and robust record, I'm not convinced that it upends our understanding of the role of mountain ranges in weathering and global cooling.

References cited in review

Carroll, A.R., Graham, S.A., Smith, M.E., 2010. Walled sedimentary basins of China. Basin Research 22, 17–32. https://doi.org/10.1111/j.1365-2117.2009.00458.x

Dellinger, M., Gaillardet, J., Bouchez, J., Calmels, D., Louvat, P., Dosseto, A., Gorge, C., Alanoca, L., Maurice, L., 2015. Riverine Li isotope fractionation in the Amazon River basin controlled by the weathering regimes. Geochimica et Cosmochimica Acta 164, 71–93. https://doi.org/10.1016/j.gca.2015.04.042

Reply: We fully agree with these insightful comments that the continental weathering flux is effectively balanced by the degassing flux within the framework of the Cenozoic carbon cycle, while tectonic uplift may primarily modify surface weatherability rather than the net carbon flux (e.g., Caves et al., 2016). In fact, the aim of our study is not to argue that silicate weathering in the Tibetan Plateau cannot influence Earth's carbon cycle. Rather, we try to demonstrate that the Li flux supplied by uplift-derived silicate weathering may be insufficient to significantly alter the seawater Li cycle. This is because the marine Li cycle is likely more strongly controlled by inputs from seafloor hydrothermal systems and reverse weathering processes (Weldeghebriel et al., 2023 SA; Cai et al., 2023 GCA), whereas the contribution from silicate weathering of tectonically active regions is comparatively smaller. In addition, continental Li inputs may be substantially modulated by non-weathering processes, such as hydrological conditions and aridity (Zhang et al., 2022 NC; Xu et al., 2022 Geology), as well as by geothermal waters (e.g., the Yarlung Zangbo River; Zhang et al., 2022 EPSL).

At present, an increasing number of studies directly interpret seawater Li isotopic records as indicators of silicate weathering in deep time, which may lead to biased conclusions. Using the Cenozoic uplift of the Tibetan Plateau as a case study for assessing the potential influence of continental weathering on the marine Li cycle, our work applies multiple flux-constrained upscaling scenarios. These include estimation of the dissolved Li flux derived from weathering based on actual carbon consumption, as suggested by Reviewer #1. Our results indicate that Li fluxes driven by silicate weathering of the Tibetan Plateau may be insufficient to drive the observed evolution of seawater Li isotopes. This finding has important implications for interpreting Li isotope records in Earth history in terms of silicate weathering and associated carbon consumption processes.

In the revised manuscript, to better assess the potential impact of Li flux variations, we employed four complementary approaches: 1) Erosion-based scaling: We assumed that past Li export varied proportionally with Neogene changes in plateau erosion flux (Métivier et al., 1999) (Fig. 5B). 2) Silicate weathering reconstruction: We coupled carbon consumption fluxes with major cation and lithium loss to constrain Li flux based on reconstructed silicate weathering rates (Clift et al., 2024) (Fig. 5C). 3) W/D–Li flux relationship: We applied empirically derived relationships among Li flux, δ^7Li, and the weathering-to-denudation (W/D) ratio from modern river systems (Pogge von Strandmann et al., 2021) to infer past Li flux from isotopic signatures (Fig. 5D). 4) Constant weathering scenario: We incorporated constant weathering fluxes constrained by Be isotope records (Willenbring & von Blanckenburg, 2010) (Fig. 5E).

We tested these four scenarios independently. Collectively, they encompass the full range of current scientific understanding regarding Neogene weathering-driven Li fluxes on the Tibetan Plateau. Our model results show that, regardless of whether the riverine Li flux from the Tibetan Plateau increased or remained constant over the past 15 Myr, the resulting plateau-derived Li input to the ocean produces only a relatively flat trend in seawater δ^7Li (Fig. 5). These findings indicate that riverine inputs from the Tibetan Plateau—despite rapid erosion and global cooling—are unlikely to account for the observed increase in seawater δ^7Li over at least the past 15 Myr. Our results show that the sustained low δ^7Li values observed in high-erosion monsoonal regions (southern HTP) and the long-term decrease in δ^7Li in arid northern basins cannot be fully explained by erosion-driven intensity decline alone. These contrasting spatial and temporal patterns highlight that lithology, hydrology, climate regime, and erosion thresholds jointly shape the weathering system. By integrating continuous records from both the northern and southern Tibetan Plateau, our study provides broader regional constraints and reveals several unexpected features, including the persistence of extremely low δ^7Li values once an erosion threshold is exceeded. We believe these findings offer important nuances to our understanding of how tectonically active ranges modulate weathering processes, even if they do not overturn the global framework of carbon cycle mass balance.

To avoid any misunderstanding on the part of the readers, we further elaborated on our aforementioned understanding in Lines 300-317 as "It is essential to constrain both the Li flux and isotopic composition input from the Tibetan Plateau. Reconstructing past variations in riverine Li

flux from the Tibetan Plateau remains highly challenging due to the absence of direct reconstructions of ancient river discharge and Li concentrations. We first quantified the modern riverine Li fluxes across the plateau through statistical analyses (Table 1). The results indicate that major rivers originating from the Tibetan Plateau collectively export a Li flux of approximately ~1.5×10^9 mol/yr to the ocean (accounting for ~18% of global river input) (Table 1). We then employed four scenarios to constrain the Li flux evolution of the Tibetan Plateau over the past 15 Myr (see Methods). First, the past Li flux varied proportionally with variations in the Neogene erosion flux of the northern and southern plateau (Fig. 1B) (63). Second, the atmospheric CO_2 consumption flux by silicate weathering of the Tibetan Plateau (*79*) constrain the dissolved Li flux based on the C-Li coupling assumption that Li is congruently released, similar to the way base cations (Na, K, Ca and Mg) are depleted during silicate weathering (*13*) (Fig. 5C). Third, the Li flux through time is constrained by the empirically-derived relationship with δ^7Li, the weathering-to-denudation (W/D) ratio from modern rivers (64), and our measured δ^7Li signatures (Fig. 5D). Fourth, temporal changes in Li flux is linked to the constant Tibetan Plateau erosion flux based on based on constant exhumation and erosion rates (*47, 58, 65*) (Fig. 5E). Subsequently, we tested the effect of these four different scenarios separately in our Li mass balance model and these four scenarios essentially encompass the current understanding of the weathering-induced lithium flux on the Tibetan Plateau during the Neogene period.

And in Liners 327-350 as "Our model results suggest that, regardless of increasing or constant riverine Li flux from the Tibetan Plateau over the past 15 Myr, the estimated Li weathering flux from the Tibetan Plateau can only cause the tendency of seawater δ^7Li to be relatively flat (Fig. 5). These calculations demonstrate that riverine input from the Tibetan Plateau, driven by rapid erosion and global cooling, is unlikely to account for the observed rise in seawater δ^7Li at least over the past 15 Myr.

Collectively, the results of the modelling that takes into account our records suggest that Neogene seawater δ^7Li records are unlikely to reflect enhanced silicate weathering driven by mountain uplift, due to the mixing of other source and sink fluxes. Our results do not imply that weathering of the Tibetan Plateau is incapable of influencing Earth's carbon cycle. Rather, they indicate that the Li flux supplied by silicate weathering of tectonically active regions alone may be insufficient to measurably alter the marine Li cycle. This implies that changes in the alteration of the oceanic crust, uptake of Li into marine sediments and continental weathering outside active mountain belts have to be taken into account to explain the δ^7Li seawater record in the last 15 Ma. Indeed, the oceanic Li budget is strongly modulated by seafloor hydrothermal inputs and submarine reverse weathering (*18-19*), which may outweigh the continental contribution. In addition, Li export from continents can be substantially influenced by non-weathering processes, including hydrological variability and aridity (*16-17*), as well as inputs from geothermal waters (*83*). By examining the Cenozoic uplift of the Tibetan Plateau as a natural experiment, our study demonstrates the limitations of using marine Li isotopes alone to infer long-term weathering-driven CO_2 consumption. We therefore emphasize the importance of reconstructing silicate weathering

histories in major orogenic regions, such as the Tibetan Plateau and the Andes, which provide the most direct geological archives of tectonically driven carbon cycling. Our Li isotope records and modelled results underscore that the Li cycle associated with hydrothermal systems and submarine reverse weathering is needed to explain the observed Neogene seawater δ^7Li records, and that it is important to better constrain these complex marine source-sink processes."

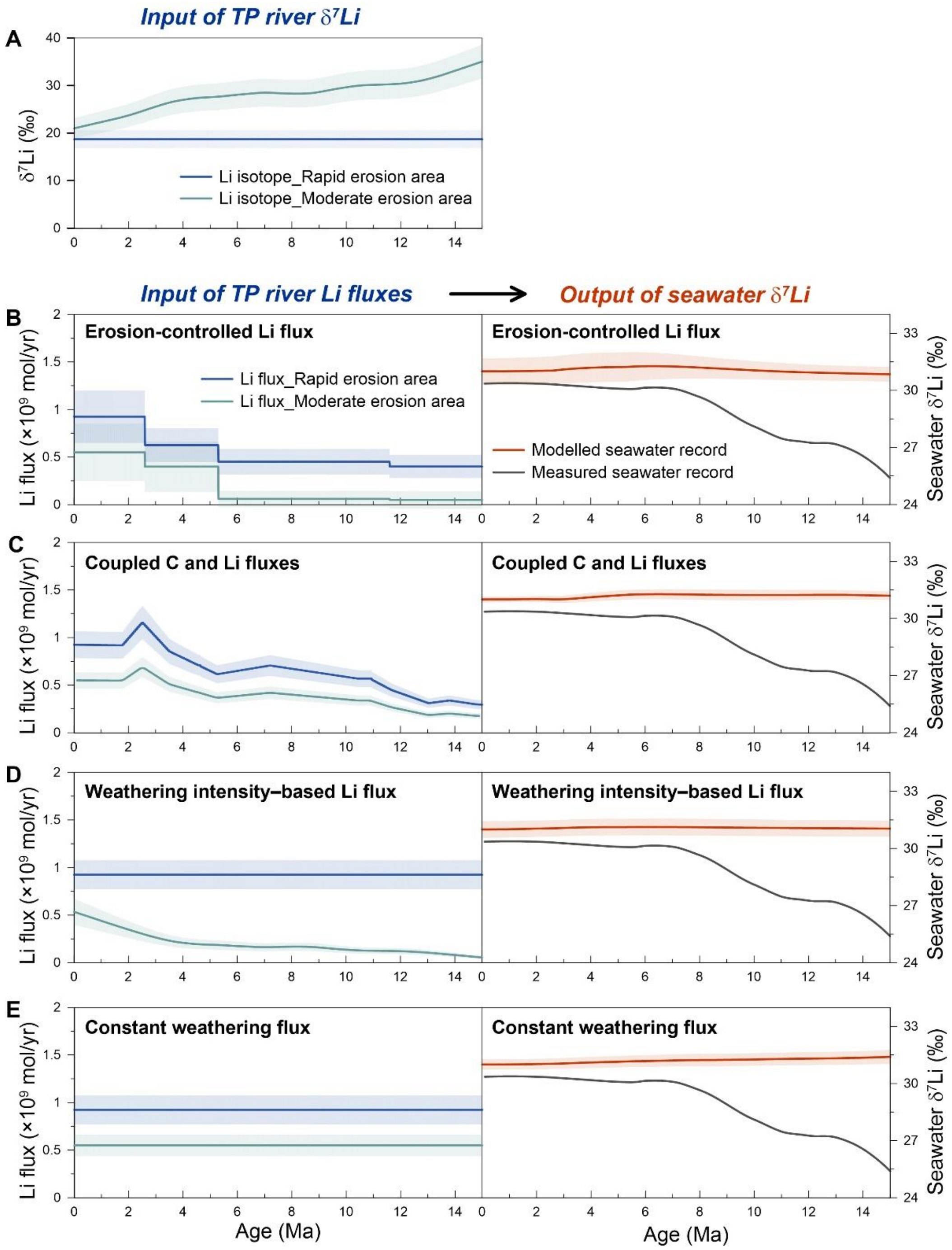

Fig. 5. Dynamic simulation of seawater Li isotope changes under reconstructed Li fluxes and δ^7Li values from the Tibetan Plateau (TP). (A) The evolution of δ^7Li_{riv} of the rapid erosion area (blue line) and moderate erosion area (green line) of the Tibetan Plateau. (B–E) The evolution of input river Li fluxes and model result of seawater δ^7Li, (B) based on sediment accumulation rates of

surrounding basins (63) (Scenario 1), (C) coupled with C and Li fluxes (63) (Scenario 2), (D) based on correlation between Li yield, δ^7Li, and W/D (*64*) (Scenario 3), (E) based on constant weathering flux (based on Be isotope records) (*65*) (Scenario 4). The measured seawater δ^7Li curve is from ref. (*7*). See methods for details of the four scenarios. None of the four scenarios can reproduce the rise in seawater δ^7Li since 15 Ma.

We would like to express our sincere gratitude to the editor and three reviewers for their recognition of our new data and efforts in revising the manuscript. Below is our response to the reviewers' comments. The comments are in black and our replies are in blue. Line numbers refer to **the tracked manuscript**. The underlined text refers to that in the Main text.

Response to Reviewer #1:

Reviewer #1 (Remarks to the Author):

The authors have undertaken a monumental revision. Even the title and the author list have changed. Entire sections of the original text were removed and replaced with a new analysis, particularly regarding the modeling component. I must admit, I am somewhat perplexed; the revision is so extensive that we are essentially looking at a different paper. I would jokingly suggest that this level of rewriting almost warrants including the reviewers in the author list. All kidding aside, it does raise questions about the value of the initially submitted study, since—data aside—nearly everything else was incorrect.

That said, in their response to the reviewers, the authors mention they will be using the lithium-carbon coupled model from Vigier and Goddéris (2014). The primary contribution of V&G was the inclusion of the carbon cycle. I should note that the authors addressed my comments on this modeling point by integrating a discussion on the coupling of Li cycles and its isotopes with the carbon cycle. Overall, the manuscript has been significantly improved. I have no specific further comments; I believe this work, based on high-quality data and a simple yet coherent modeling approach, deserves publication. However, the complete overhaul of the text remains striking. I naturally leave the final decision to the Editor.

Reply: We greatly appreciate the reviewer's acknowledgment of the significant improvements made to the manuscript, particularly regarding the integration of the lithium-carbon coupled model and the enhanced discussion of Li cycles and isotopes in relation to the carbon cycle. We understand the reviewer's concern regarding the extensive nature of the revisions. While the changes were substantial, they were undertaken with the goal of strengthening the scientific rigor and coherence of the manuscript, and we are pleased that the reviewer recognizes the overall improvement. We also wish to emphasize that it was precisely the reviewer's insightful comments—combined with the acquisition of new Siwalik data—that prompted us to undertake this major revision, including substantial updates to both the modeling and data analysis. Thank you again for the valuable feedback and for considering our manuscript for publication.

Response to Reviewer #2:

Reviewer #2 (Remarks to the Author):

This revised manuscript has undergone substantial modifications that have effectively addressed my concerns. I think one additional minor issue is still the modeling.

Reply: We thank the reviewer for the positive feedback and for acknowledging that the substantial modifications have effectively addressed their concerns.

For Scenarios 1 (erosion flux-based Li flux) and 3 (weathering intensity-based Li flux), the authors should consider whether the drainage patterns of the northern and southern Tibetan Plateau remained consistent throughout the Neogene. Any changes in these patterns could alter the division between the northern and southern regions. To my knowledge, significant drainage reorganizations occurred during this period. For instance, the Yellow River formed relatively recently (e.g., ~1.25 Ma; Wang et al., 2022), and the modern Irrawaddy River originated in the late Miocene – Pliocene (~7 Ma; Jonell et al., 2022).

Reply: We appreciate the reviewer's valuable comments. Indeed, significant drainage reorganizations occurred during this period and would influence the division. However, both the Yellow River and Irrawaddy River drainages lie entirely within the high erosion area and moderate erosion area delineated in our study. Therefore, while these changes are significant at the river system scale, they do not alter the total sediment and lithium fluxes between the northern and southern Tibetan Plateau as a whole. We have added a clarification in the revised manuscript to address this point in Lines 389-393 as "Although there might be cases of river basin reorganization during this period, the large-scale drainage reorganization that have been reported, such as the Yellow River and Irrawaddy River (68-69), are completely occurred within the high and moderate erosion regions delineated in our study. These drainage reorganizations would not alter the total sediment and Li fluxes of the high and moderate erosion regions"

The modern riverine Li flux has been updated recently. That means the value of 23‰ in Table 2 is no longer accurate. Please refer to the references Läuchli et al. (2025) and Yang et al. (2025). Also, please correct the typo in Table 2 (3.65 × 16 mol for seawater Li reservoir)

Reply: We thank the reviewer for pointing out the updated values in Table 2. We have now updated these values and re-generated the model results. This adjustment made our parameters more accurate without affecting the conclusion.

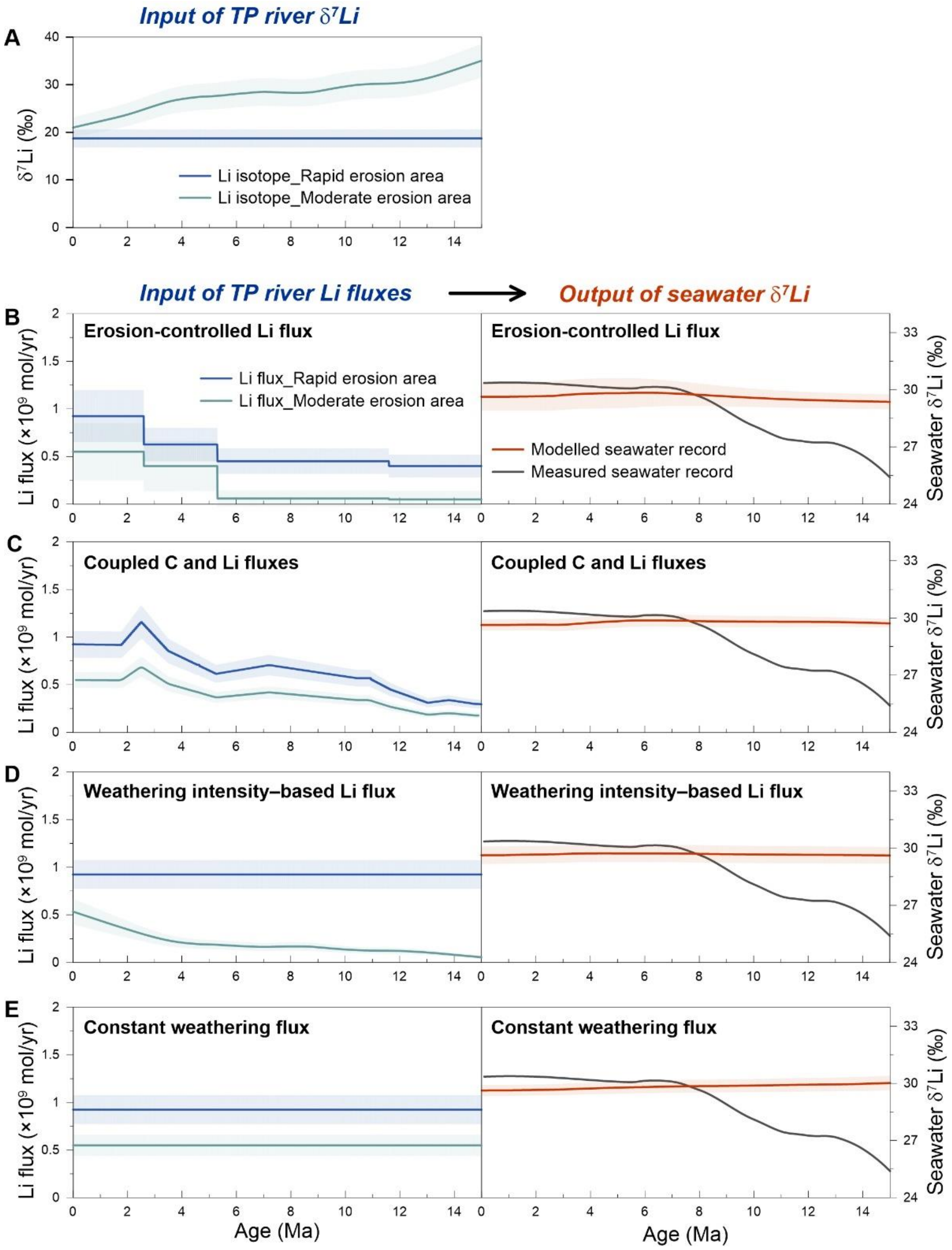

We appreciate the reviewer's careful reading. The “3.65 × 16 mol” for the seawater Li reservoir has been corrected to 3.65×10^{16} mol in the revised Table 2.

We thank the reviewer again for these constructive suggestions, which have helped improve the accuracy and rigor of our manuscript.

Response to Reviewer #3:

Reviewer #3 (Remarks to the Author):

In this revised manuscript, Yang and co-authors add an additional record from the Siwaliks to estimate d7Li across a longer timeframe. They find that the Siwalik record does not change much

over the Neogene (or, more precisely, there is a fair amount of variability, but no directional change) and the reconstructed river values are themselves fairly low. They conclude that weathering in the Himalayas cannot be a source of rising seawater d7Li and that other fluxes (either from the seafloor or from other continental areas) must be responsible for the rise of seawater d7Li.

I found this version of the manuscript to be substantially better than the previous version and addresses the two central critiques of my previous review. First, I was skeptical that the d7Li record from the Qaidam Basin could be scaled to the entire Himalaya-Tibetan Plateau system. In some sense, this concern was correct, but the new record from the Siwaliks is fascinating and also novel. I know of few other types of these records anyway, particularly from such an active orogen.

My second critique regarded the authors interpretation of how declining d7Li in the Qaidam Basin should be interpreted in terms of weathering flux and weathering intensity. Again, here, the authors have substantially revised their interpretation and now focus their interpretation on what these two records mean for weathering intensity in the Tibetan Plateau-Himalaya region and how this impacts the seawater d7Li budget. I think this is a reasonable way of interpreting the data, and it certainly sets up a number of questions that other researchers can follow up on.

Reply: We sincerely thank the reviewer for the positive and thoughtful evaluation of our revised manuscript. We are grateful that the reviewer finds this version substantially improved and acknowledges that our revisions have addressed the two central critiques raised in the previous review. In particular, we appreciate the reviewer’s recognition of the novelty of the Siwalik record and the reasonableness of our revised interpretation regarding weathering intensity and its implications for the seawater Li isotope budget.

My only comment regards the discussion of floodplain d7Li. The authors have put together an interesting compilation of floodplain d7Li, but then argue that, though d7Li tends to increase across floodplains, this cannot, in a sense, cancel out the signal of declining or constant d7Li that they find in their sections. I disagree, and I think it’s hard for the authors to know whether this 6-10‰ increase in d7Li downstream has been constant or has, actually, increased through time. If the relationship of d7Li downstream has changed through time, then it could be the changing role of floodplain weathering that partially mutes any decreasing (or constant) d7Li signal recorded in these sections.

Reply: We would thank the reviewer's comment on floodplain δ^7Li, we fully agree that this is an important and complex issue. We acknowledge that the relationship between δ^7Li and floodplain processes may not be straightforward, and as highlighted by recent work such as Hu et al. (2026, GCA), there are scenarios where δ^7Li could remain constant or even decrease downstream. Nevertheless, our current compilation, based on major world rivers, provides a representative overview of general trends. We also recognize that uncertainties remain regarding weathering processes and lithium isotope fractionation in floodplain environments, and we agree that this warrants further investigation. In response to the reviewer's insightful comment, we have added a discussion in the revised manuscript to explicitly address these uncertainties and complexities, noting that the role of floodplain weathering through time remains an open question that merits

future research in Lines 352-357 as "Although δ^7Li tendency in floodplains can be complex (e.g., plateauing or even decreasing downstream in some modern cases (*63*) or probably varying influences of floodplains in the past periods), our compilation, based on data from major world rivers, provides a representative overview of the general trends. These results suggest that paleowater δ^7Li records from mountain ranges (e.g., the Tibetan Plateau) can still capture the stable or decreasing trends of riverine δ^7Li exported from tectonically active areas to the oceans"

We thank the reviewer again for the constructive feedback, which has helped us strengthen the manuscript.
